# Biochemical and Comparative Transcriptomic Analyses Identify Candidate Genes Related to Variegation Formation in *Paeonia rockii*

**DOI:** 10.3390/molecules22081364

**Published:** 2017-08-17

**Authors:** Qianqian Shi, Long Li, Xiaoxiao Zhang, Jianrang Luo, Xiang Li, Lijuan Zhai, Lixia He, Yanlong Zhang

**Affiliations:** 1 College of Landscape Architecture and Art, Northwest A&F University, Yangling 712100, Shaanxi, China; shiqianqian2005@163.com (Q.S.); zhangbinxiao0505@126.com (X.Z.); luojianrang@163.com (J.L.); 15991722712m0@sina.com (X.L.); 18792499294@163.com (L.Z.); 2 National Engineering Research Center for Oil Peony, Yangling 712100, Shaanxi, China; 3 College of Forestry, Northwest A&F University, Yangling 712100, Shaanxi, China; lilong1949@126.com; 4 Gansu Engineering Research Center for Tree Peony, Lanzhou 730046, Gansu, China; helixia_lz@sina.com; 5 Gansu Forestry Science and Technology Extend Station, Lanzhou 730046, Gansu, China

**Keywords:** anthocyanin biosynthesis, comparative transcriptomic, R2R3-MYB, *P. rockii*, variegation formation

## Abstract

*Paeonia rockii* is a wild tree peony species with large and dark purple variegations at the base of its petals. It is the genetic resource for various variegation patterns in tree peony cultivars, which is in contrast to the pure white petals of *Paeonia ostii*. However, the molecular mechanism underlying the formation of variegation in this plant is still unknown. Here, we conducted Illumina transcriptome sequencing for *P. rockii*, *P. ostii* (with pure white petals) and their F1 individuals (with purple-red variegation). A total of 181,866 unigenes were generated, including a variety of unigenes involved in anthocyanin biosynthesis and sequestration and the regulation of anthocyanin biosynthesis. The dark purple or purple-red variegation patterns mainly occurred due to the proportions of cyanidin (Cy)- and peonidin (Pn)-based anthocyanins. The variegations of *P. rockii* exhibited a “Cy > Pn” phenotype, whereas the F1 progeny showed a “Pn > Cy” phenotype. The *CHS*, *DFR*, *ANS*, and *GST* genes might play key roles in variegation pigmentation in *P. rockii* according to gene expression and interaction network analysis. Two R2R3-MYB transcription factors (c131300.graph_c0 and c133735.graph_c0) regulated variegation formation by controlling *CHS*, *ANS* and *GST* genes. Our results indicated that the various variegation patterns were caused by transcriptional regulation of anthocyanin biosynthesis genes, and the transcription profiles of the R2R3-MYBs provided clues to elucidate the mechanisms underlying this trait. The petal transcriptome data produced in this study will provide a valuable resource for future association investigations of the genetic regulation of various variegation patterns in tree peonies.

## 1. Introduction

Tree peony is a woody shrub of the *Moutan* section of the genus *Paeonia*, family Paeoniaceae. It is an important traditional ornamental plant with large, attractive and distinct petal colors. There are ten wild species of tree peony: *P. suffruticosa*, *P. ostii*, *P. rockii*, *P. jishanensis*, *P. qiui*, and *P. decomposita* belonging to subsect Vaginatae and *P. delavayi*, *P. lutea*, *P. potanini* and *P. ludlowii* belonging to subsect Delavayanae [1]. Interestingly, *P. rockii* has distinct black variegation at the base of white petals, whereas *P. ostii* lacks petal variegation. Flower color is a commercially important characteristic of tree peonies, and there is much interest in cultivars that bear flowers with unique colors. To date, about 1500 cultivars with a wide variation in color hues and patterns have been produced by conventional breeding in China; some of these cultivars exhibit variegation at the base of each petal or two distinct colors on individual petals. Most cultivars with variegation are most likely to be the progeny of *P. rockii* [2]. Because the variegation on the petals enhances the ornamental value of tree peony, clarifying the mechanism underlying variegation formation in *P. rockii* has great significance.

In plant science, anthocyanins are the most studied and best understood compounds, and their metabolic pathways have been extensively described [3,4]. The activity of anthocyanin biosynthesis enzymes is primarily regulated by transcription factors, including R2R3-MYB, bHLH and WD40 [5]. Variegation formation on the petals is due to the spatially and temporally restricted deposition of anthocyanin pigments, which is controlled by the spatial and temporal expression of anthocyanin-related genes [4]. Cyanidin (Cy)-based glycosides, which accumulate abundantly at the basal petal, result in blotch formation in the Xibei tree peony [6]. Co-expression of *PsCHS*, *PsF3’H*, *PsDFR* and *PsANS* has been reported to be responsible for purple spot pigmentation in the tree peony [7]. The spot pattern is associated with the differential expression of *DFR2* in the red-purple spot in *Clarkia gracilis* [8].

The regulation mechanisms responsible for the restricted pigment deposition have been revealed in some model plants [9,10]. The R2R3-MYB transcription factors are predominantly responsible for the variegation patterns. In snapdragons, *Rosea1* and *Rosea2* determine whether the petals are fully pink, whereas *Venosa* regulates venation [9,10]. In *C. gracilis*, *CgMYB1* activates spot formation; different *CgMYB1* alleles expressed in different domains, leading to spot formation in different petal locations [11]. In *Phalaenopsis spp*, *PeMYB11* has been verified to regulate variegated pigmentation by activating the expression of anthocyanin biosynthetic genes *PeF3H5*, *PeDFR1* and *PeANS3* [12]. However, the mechanism on variegation formation in *P. rockii* has not been fully understood.

It is well known that tree peony has a huge genome (13–16 Gb) [13], which often exhibits high heterozygosity and gametophytic self-incompatibility, and a long life cycle (3–4 years from sowing to anthesis). In the absence of a complete genome sequence, RNA sequencing technology based on Illumina sequencing is the most effective and popular tool to obtain information on the expressed fraction of the genome. To date, several anthocyanin biosynthesis-related genes in tree peony have been identified [7,14,15,16,17,18,19,20]. However, the overall molecular mechanisms underlying variegation formation in *P. rockii* are not known.

In the present study, a comparative transcriptomics analysis was conducted using next-generation sequencing to examine the transcript profiles from the petals of *P. rockii* (PR), *P. ostii* (PO) and F1 progeny individuals (RO) with different pigmented patterns. Candidate genes involved in variegated pigmentation process were obtained by annotating and analyzing DEGs. The objective of this study is to reveal the mechanisms on complicated variegation traits. Understanding the molecular mechanisms of variegated pigmentation in *P. rockii* will advance the knowledge of the genetics underlying variegation formation in tree peony and provide a valuable resource for the identification of genes expressed in the two wild populations.

## 2 Results

### 2.1. F1 Progeny

**Figure 1:**
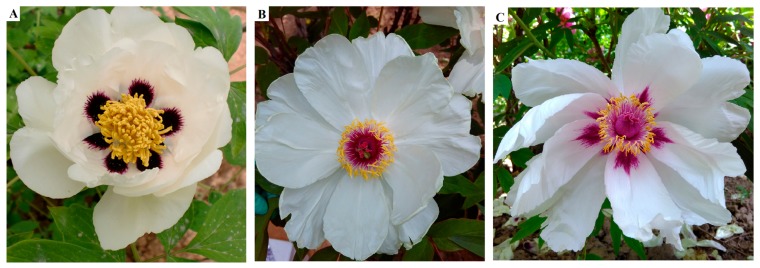
Fully open flowers of individuals selected for sequencing. (**A**) *Paeonia rockii*; (**B**) *Paeonia ostii*; (**C**) F1 individual derived from crossing between *P. rockii* and *P. ostii*.

To determine the genetic background of the variegation trait, F1 plants (RO) were derived from crossing between PO and PR. All of the 60 F1 plants exhibited variegation at the base of the petals (Figure 1). Therefore, the variegation of PR exhibited dominant inheritance.

### 2.2. Color Indices

According to the Royal Horticultural Society Colour Chart (RHSCC), the background color in the PR and RO flowers at stage 5 was the same white as the PO petals, whereas the colors of the variegations in the PR and RO flowers at stage 5 were difference. The color of the variegation was dark purple in PR and purple-red in RO (Table 1). The L*, a* and C* values of the variegation in the RO petals were higher than those in the PR petals. However, the b* value of the variegation was lower in the RO petals than that in PR. Conversely, the color indices of the background in the PR and RO progeny petals were similar to those in PO. These color indices showed that the color of the variegation in RO petals was lighter and more vivid than that in PR. Therefore, these analysis results were consistent with the visual results.

### 2.3. Spatial Location of the Pigments

**Figure 2:**
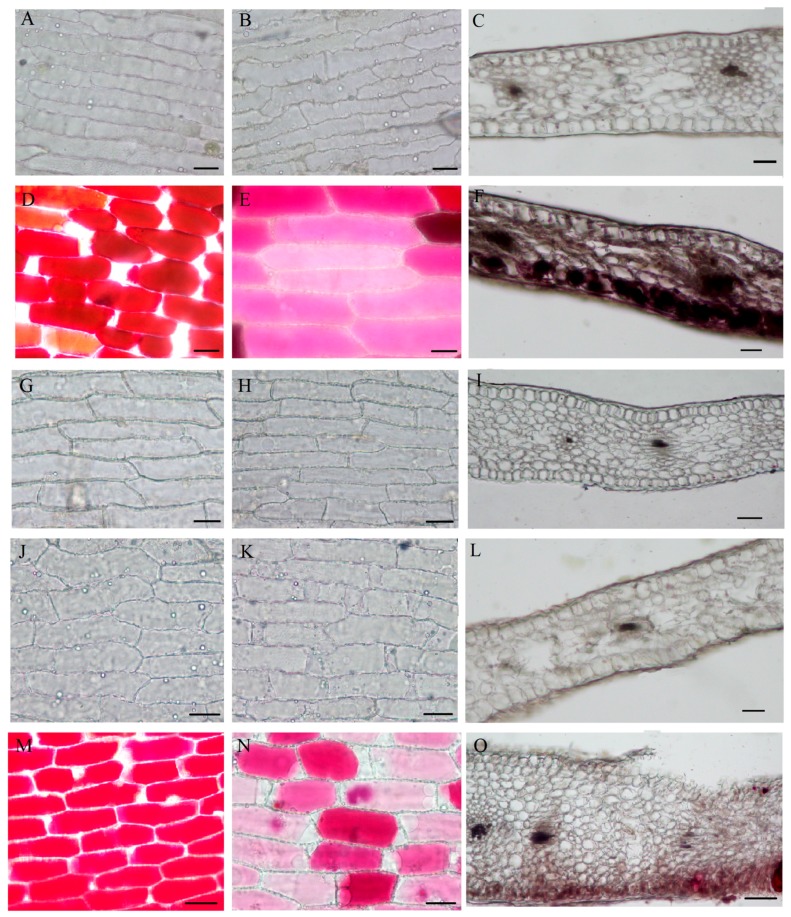
Cellular features of the flower materials. Bar = 100 mm. ((**A**)–(**C**), (**G**)–(**I**) and (**J**)–(**L**)) the abaxial, adaxial and cross section of the background petals of *P. rockii*, *P. ostii* and the F1 progeny, respectively; ((**D**)–(**F**) and (**M**)–(**O**)) the upper epidermis, lower epidermis and cross section of the background petals of *P. rockii* and the F1 progeny, respectively.

To elucidate the mechanism underlying variegation formation, the spatial location of the pigment was examined within the PR, PO and RO flower petals. As shown in Figure 2C,F,I,L,O, the cross sections of the petals showed a typical structure consisting of an upper epidermis, palisade mesophyll, spongy mesophyll, and lower epidermis. No pigmented cells were found in the non-variegated petals (Figure 2C,I,L). In contrast, the pigmented cells were primarily located in the upper and lower epidermises and the palisade mesophyll (Figure 2F,O).

The pigmented cells corresponding to variegation in PR were also located in the inner (adaxial) epidermis, which might contribute to the much deeper color of the variegation in PR (Figure 2F). The colored cells in the variegated petals were primarily located in the upper and lower epidermal layers, although the upper epidermal cells showed much deeper color than the lower epidermal cells (Figure 2D–N). In the non-variegated petals, the epidermal cells were colorless (Figure 2A,B,G–K). Therefore, the variegation was caused at least in part by differences in the spatial location of the colored cells within the tissues.

**Figure 3:**
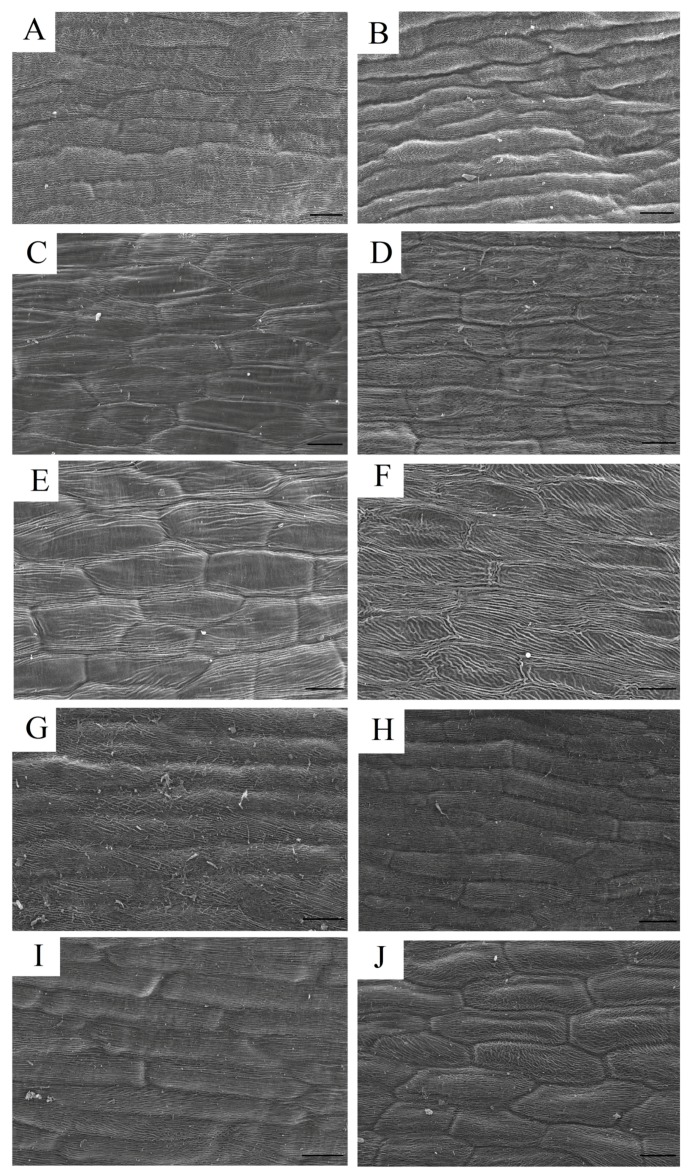
Scanning electron micrograph (SEM) of papillate cells from the outer epidermis of petals from the materials. Bar = 40 μm. ((**A**,**B**), (**E**,**F**) and (**G**,**H**)) the upper and lower epidermis of the background petals of *P. rockii*, *P. ostii* and the F1 progeny, respectively. ((**C**,**D**) and (**I**,**J**)) the upper and lower epidermis of the variegated petals of *P. rockii* and the F1 progeny, respectively.

To the best of our knowledge, the shape of the pigmented cells influences their optical properties and thus affects our sensation of color. To analyze interactions between color and surface geometry, the epidermal cell shapes in the variegated and non-variegated petals were examined by scanning electron microscopy (Figure 3).

The cell shapes between the variegated and non-variegated petals were similar. All epidermal cells were elongated and explanate (Figure 3). These results suggested that the variegation formation in tree peony was not associated with the epidermal cell shape.

### 2.4. Qualitative and Quantitative Analysis of Pigments

The HPLC analysis revealed that the pigments in the variegations in the *P. rockii* and F1 progeny petals contained four anthocyanins (Cy3G5G, Cy3G, peonidin (Pn) 3G5G and Pn3G) (Table 2). The variegation in PR showed a “Cy > Pn” phenotype, with Cy3G representing the most abundant anthocyanin (28.36 ± 0.063 mg/g), whereas RO had a “Pn > Cy” phenotype with high Pn3G5G (4.27 ± 0.046 mg/g) and Cy3G5G contents (2.51 ± 0.011 mg/g). No anthocyanins were detected in the background petals in RO and PR or the whole petals of PO.

Additionally, flavonoid, flavone and flavonol were detected in the variegations, including Qu 3,7-di-*O*-glucoside, Km 3,7-di-*O*-glucoside, Is 3,7-di-*O*-glucoside, Qu 3-*O*-glucoside, Lu 7-*O*-glucoside, Ap 7-*O*-glucoside, Is 3-*O*-glucoside, and Ap 7-*O*-neohesperidoside (Table 3). Tremendous differences in the compositions were found in different petals (Table 3). In the variegations of *PR* petals, the main component was Ap 7-*O*-glucoside (3.64 ± 0.062 mg/g), followed by Lu 7-*O*-glucoside (3.08 ± 0.010 mg/g), Km 3,7-di-*O*-glucoside (2.25 ± 0.034 mg/g), Qu 3-*O*-glucoside (2.16 ± 0.036 mg/g), Is 3,7-di-*O*-glucoside (1.69 ± 0.037 mg/g), Ap 7-*O*-neohesperidoside (1.58 ± 0.053 mg/g), Is 3-*O*-glucoside (1.01 ± 0.055 mg/g) and Qu 3,7-di-*O*-glucoside (0.95 ± 0.021 mg/g). However, the most abundant compound in the variegations of the F1 progeny was Is 3,7-di-*O*-glucoside (3.41 ± 0.055 mg/g), followed by Lu 7-*O*-glucoside (2.42 ± 0.085 mg/g), Km 3,7-di-*O*-glucoside (1.38 ± 0.031 mg/g) and Is 3,7-di-*O*-glucoside (1.26 ± 0.058 mg/g). In contrast, the main compositions detected in the *PO* petals and the non-variegated petals from PR and RO were Ap 7-*O*-neohesperidoside, Ap 7-*O*-glucoside, Km 3,7-di-*O*-glucoside and Lu 7-*O*-glucoside (Table 3).

TFs in the non-variegated areas were twice as much as the TFs in the variegated areas, and the TAs were four times higher in the variegations of PR than in RO. Furthermore, the CI was used to determine the co-pigmentation effect between anthocyanins and flavonoids. The CI value in the variegations of RO was higher than that in PR (Table 1). The petal color appeared to be strongly influenced by the variety and concentration of pigments, prompting us to further investigate the molecular processes at the transcriptome level.

### 2.5. Transcriptome Sequencing and Annotation

To identify the key genes involved in variegation formation in PR, we performed a comparative transcriptomic analysis among three different pigmentation patterns from PR, PO, and RO using the Illumina HiSeq^TM^ 2500 technique. A total of 181,866 unigenes from nine libraries were generated, of which 57,913 (31.84%), 43,307 (23.81%), 34,125 (18.76%), 18,986 (10.44%), 33,409 (18.37%), 24,154 (13.28%), and 47,410 (26.07%) unigenes were annotated to the NCBI Non-redundant Protein database (NR), Protein Family (Pfam), SwissProt protein database (SwissProt), Kyoto Encyclopedia of Genes and Genomes database (KEGG), Gene Ontology (GO) and Cluster of Orthologous Groups of Proteins database (COG) and euKaryotic orthologous groups (KOG) database, respectively (Table S1).

When compared with the KEGG database, 130 pathways were associated the unigenes (Table S2). These pathways represented metabolism, genetic information processing, organism systems and cellular processes. As far as we known, four secondary metabolic pathways were related to flower pigmentation: phenylpropanoid biosynthesis (250, 1.04%), flavonoid biosynthesis (64, 0.26%), anthocyanin biosynthesis (4, 0.02%), and flavone and flavonol and biosynthesis (6, 0.02%) (Table S2). Of these pathways, 44, six, five, two, five, two, three, four and four unigenes were annotated as PAL, CHS, CHI, F3H, F3’H, FLS, DFR, ANS and AOMT, respectively, three unigenes were annotated as 3GT, and one unigene was aligned to 5GT (Table S3). Additionally, 64 candidate unigenes were predicted to encode glutathione S-transferase (GST) proteins, which are required for transporting pigments to vacuoles (Table S4). In higher plants, the gene expression of anthocyanin biosynthesis pathway was regulated by a ternary complex consisting of the R2R3-MYB, bHLH, and WD40 transcription factors [21]. Accordingly, 88, 16 and 44 unigenes were predicted to encode R2R3-MYBs, bHLHs, and WD40s, respectively (Table S4).

### 2.6. Comparative Analysis of the P. rockii, P. ostii and F1 Progeny Transcriptomes

**Figure 4:**
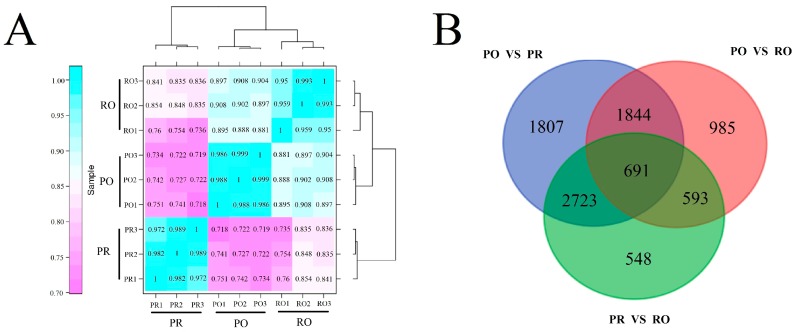
Gene expression profiles in three differentially colored tree peonies. (**A**) Cluster dendrogram of gene expression profiles between biological replicates and among different tree peonies. The color scale represents Pearson correlation coefficients among different samples. The higher the Pearson correlation coefficient, the closer the relationship between two libraries is. The blue represents a closer relationship, and pink represents a distant one transcriptome relationship between different samples; (**B**) Comparison in pairs of differentially expressed genes (with an FDR < 0.001 and a |log2 ratio| > 1) in different groups. The Venn diagram depicts the overlaps between each pairwise comparison. The Venn diagram was constructed online using Venny (http://bioinfogp.cnb.csic.es/tools/venny/).

The gene expression variations in the nine RNA-seq samples were divided into clustered into three groups with three replicate samples according to their special coloration patterns (Figure 4A). Additionally, the levels of gene expression in RO were more similar to those observed in PO than PR.

To identify candidate genes involved in variegation formation in *P. rockii*, a comparative analysis of the PR, PO and RO libraries was performed. Based on the threshold value of FDR < 0.001 and |log2 ratio| > 1, 7065 unigenes were identified as differentially expressed genes (DEGs) in the PO petals compared with the PR petals (Figure 4B), including 3147 up-regulated genes and 3918 down-regulated genes (Figure 4B), 4113 DEGs were identified in PO vs. RO, including 1925 up-regulated genes and 2188 down-regulated genes, and 4947 DEGs in PR vs. RO, including 2615 up-regulated genes and 2332 down-regulated genes (Figure 4B). Moreover, a total of 691 unigenes showed significantly different expression levels in all of the three groups (Figure 4B).

GO enrichment analysis was performed for the DEGs in the three groups. 9191 DEGs were categorized into 50 functional groups, including 20, 14 and 16 groups in the biological process, cellular component and molecular function categories, respectively. Metabolic process and cellular process were the most frequent terms in the biological process category, cell part and organelle accounted for the major proportions in the cellular component category, and catalytic activity and binding were the dominant functions in the molecular function category (Figure S1).

Furthermore, the DEGs were aligned to the KEGG database for a functional annotation analysis. As expected, in the PO vs. PR comparison, four pathways related to pigmentation were identified: phenylpropanoid biosynthesis (39 DEGs, ko00940), flavonoid biosynthesis (10 DEGs, ko00941), flavone and flavonol biosynthesis (2 DEGs, ko00944) and anthocyanin biosynthesis (1 DEG, ko00942) (Table S5). For the PR vs. RO comparison, three pathways involved in pigmentation were identified: phenylpropanoid biosynthesis (29 DEGs), flavonoid biosynthesis (four DEGs) and anthocyanin biosynthesis (1 DEG) (Table S6). In contrast, phenylpropanoid biosynthesis (25 DEGs), flavonoid biosynthesis (6 DEGs), flavone and flavonol biosynthesis (one DEG) and anthocyanin biosynthesis (one DEG) were identified in the PO vs. RO comparison (Table S7).

**Figure 5:**
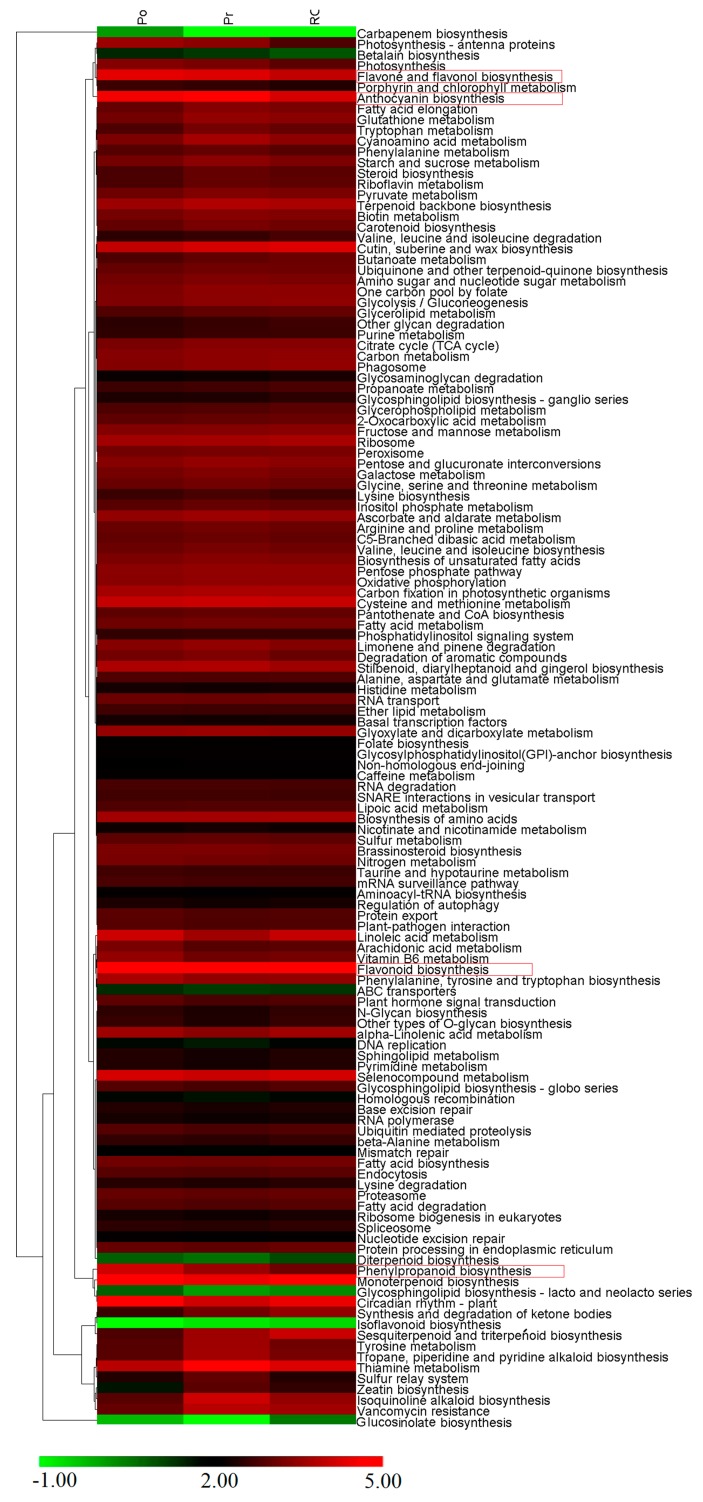
Expression profiles of 130 KEGG pathways. The heatmap is generated according to the average expression levels of the genes in each pathway based on log ratio FPKM data. The color scale represents log2 transformed FPKM values. Green indicates low expression, and red indicates high expression.

The expression profiles of the metabolic pathways identified in KEGG were clustered (Figure 5). Among the four pathways involved in pigmentation, phenylpropanoid biosynthesis and flavone and flavonol biosynthesis were abundant in PO, flavonoid biosynthesis showed a high abundance in PO and RO, and anthocyanin biosynthesis was relatively abundant in PR.

**Figure 6:**
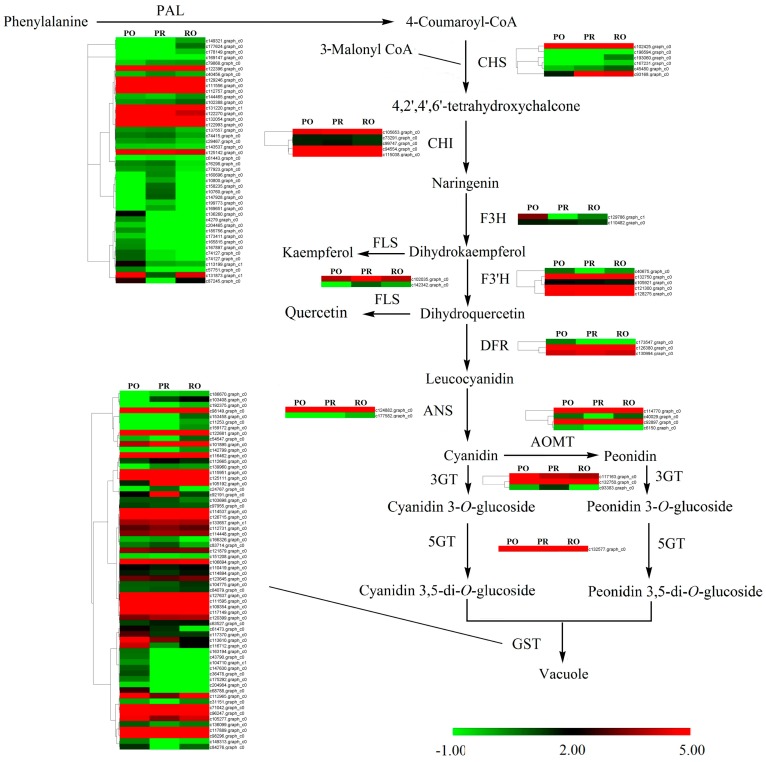
A detailed schematic of anthocyanin metabolism related to flower pigmentation in PO, PR and RO. Enzyme names and expression patterns are indicated beside each step. The expression pattern of each gene is shown in a heatmap. The color scale represents log2 transformed FPKM values. Green indicates low expression, and red indicates high expression.

Previous research has demonstrated that variegation formation in the tree peony is mainly due to the spatial biosynthesis of anthocyanins (Cy and Pn). Therefore, the expression patterns of core genes in the anthocyanin metabolic pathway were studied in detail (Figure 6). Many unigenes were expressed with significantly different levels. Most members of the *CHS*, *CHI*, *FLS*, *ANS* and *3GT* genes were up-regulated in PR or RO, whereas most members of *F3H*, *F3’H*, *AOMT* and *5GT* were up-regulated in PO (Figure 6). Among three DFR unigenes, two members were up-regulated in PO, while c126380.graph_c0 was abundant in PR and RO. Some members of the *PAL* genes were abundant in PR or RO, such as c149321.graph_c0, c79868.graph_c0 and c102388.graph_c0. As expected, the generally expression level of *GST* unigenes in PR was three times higher than that in PO and 1.5-fold of that in RO. But the changes in the expression levels of all *GST* unigenes were complicated, of which some members were significantly up-regulated in PR and RO, including c186670.graph_c0, c103408.graph_c0, c112665.graph_c0, c139960.graph_c0, c105192.graph_c0 and c114448.graph_c0 (Figure 6), and some were up-regulated in PO or RO, such as c112965.graph_c0, c105277.graph_c0, c71042.graph_c0, c117370.graph_c0, c113610.graph_c0, c96247.graph_c0, c98296.graph_c0, especially c113610.graph_c0 and c98296.graph_c0 were significantly abundant in PO.

**Figure 7:**
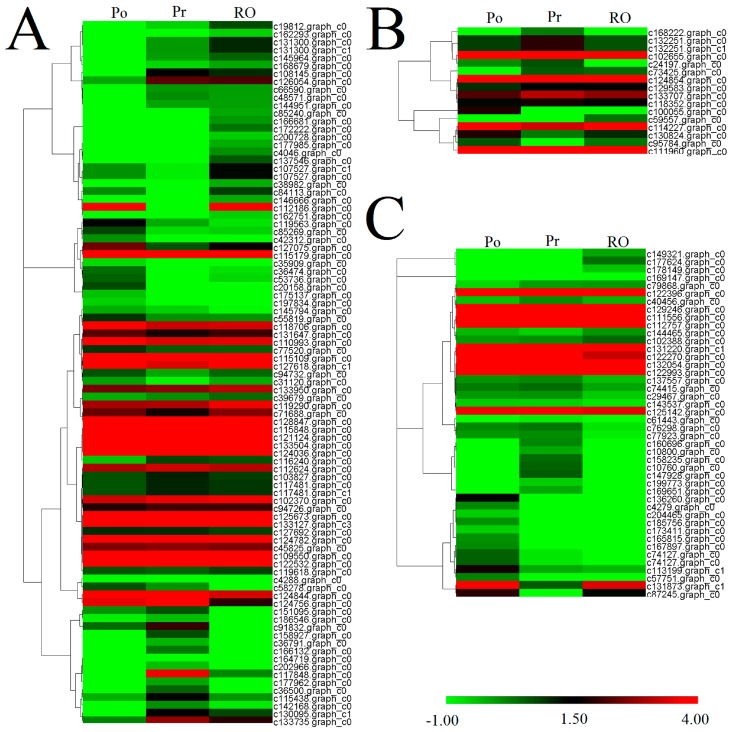
All unigenes encoding the R2R3-MYB (**A**), bHLH (**B**) and WD40 (**C**) transcription factors were hierarchically clustered and mapped using the FPKM values. Colors indicate log2 transformed FPKM values. Green indicates low expression, and red indicates high expression.

**Figure 8:**
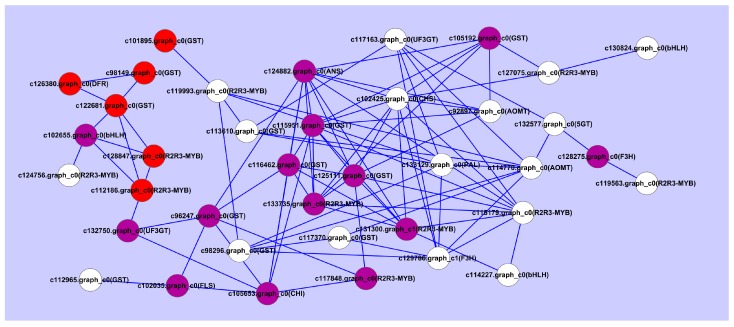
A co-expression network of the DEGs involved in pigmentation. The white, purple, and red circles represent the genes with the highest expression levels in the *P. ostii*, *P. rockii* and F1 progeny petals, respectively.

Moreover, analysis of the expression levels of the *R2R3-MYB*, *bHLH* and *WD40* unigenes indicated that some *R2R3-MYB*, *bHLH* and *WD40* genes were significantly up-regulated in PR or RO (Figure 7). A total of 28 DEGs encoding anthocyanin biosynthesis associated genes involved in variegation pigmentation were predicted (Table S8). Fourteen DEGs encoding R2R3-MYBs and 4 DEGs encoding bHLHs were also detected (Table S9). To analyze the interactions of phenylpropanoid biosynthesis, anthocyanin biosynthesis, flavonoid biosynthesis and flavone and flavonol biosynthesis with variegation formation in PR, a network comprised of differentially expressed genes was constructed (Figure 8). Obviously, the unigenes with the highest numbers of positive correlations were c124882.graph_c0 (*ANS*), c115951.graph_c0 (*GST*), c102425.graph_c0 (*CHS*), c125111.graph_c0 (*GST*), c131300.graph_c0 (*R2R3-MYB*) and c133735.graph_c0 (*R2R3-MYB*). Of these unigenes, c124882.graph_c0 (the late gene *ANS*) shared positive correlations with *CHS* (c102425.graph_c0), *AOMT* (c92897.graph_c0), five *GST* unigenes (c105192.graph_c0, c115951.graph_c0, c116462.graph_c0, c125111.graph_c0 and c96247.graph_c0) and three *R2R3-MYB* unigenes (c133735.graph_c0, c131300.graph_c0 and c115179.graph_c0).

Meanwhile, c131300.graph_c0 (R2R3-MYB transcription factor), which was highly abundant in PR, shared positive correlations with ten unigenes, including two *R2R3-MYB* unigenes, three *GST* unigenes, one *ANS* unigene, one *CHS* unigene, one *AOMT* unigene and one *bHLH* unigene. Moreover, five GST genes that exhibited much higher expression levels in PR than in PO and RO all belonged to the large hubs. So c131300.graph_c0 and c133735.graph_c0 may influence the variegation formation of *P. rockii* by regulating *CHS*, *ANS*, *AOMT*, *GST* genes.

### 2.7. Validation of the Expression Levels of Differentially Expressed Genes at Different Stages

**Figure 9:**
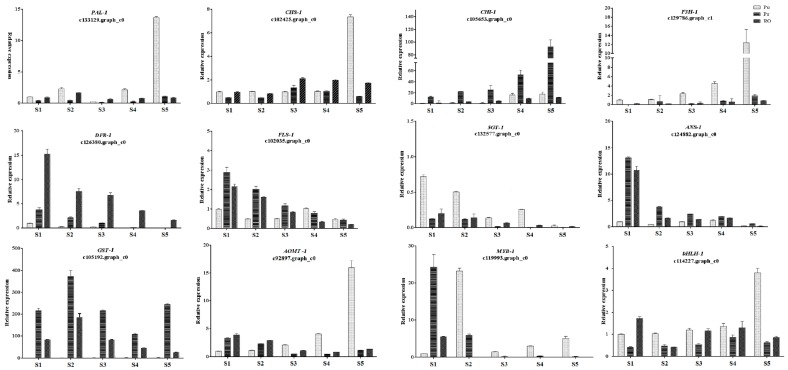
qRT-PCR analysis of twelve pigmentation-related candidate unigenes in the materials. The qRT-PCR analysis was performed using RNA from the petals at each floral developmental stages (S1–S5).

To validate the expression profiles obtained from the transcriptome data, twelve pigmentation-related DEGs were selected for qRT-PCR analysis to evaluate their expression patterns in the PO, PR and RO petals during flower development. As shown in Figure 9, *PAL-1* (c133129.graph_c0), *CHS-1* (c102425.graph_c0), *F3H-1* (c129786.graph_c1), *5GT-1* (c132577.graph_c0), *AOMT-1* (c92897.graph_c0), *MYB-1* (c119993.graph_c0) and *bHLH-1* (c114227.graph_c0) showed higher expression levels in PO than in PR and RO. Among these genes, the expression of *F3H-1* was extremely high in PO petals, conversely, it was barely detected in PR and RO petals throughout the entire flower pigmentation process. *MYB-1* (c119993.graph_c0) up-regulated at the stage 1 of PR and RO, and then down-regulated. Interestingly, *MYB-1* was significantly abundant in stage 2 of PO. Conversely, *CHI-1* (c105653.graph_c0), *DFR-1* (c126380.graph_c0), *FLS-1* (c102035.graph_c0), *ANS-1* (c124882.graph_c0), *GST-1* (c105192.graph_c0) showed significantly higher expression levels in the PR and RO petals than in the PO petals. In particular, *DFR-1*, *ANS-1* and *GST-1* were barely expressed in the PO petals during flower development. In terms of the overall trend, the expression levels of the upstream genes involved in anthocyanin biosynthesis (with the exception of *FLS*) showed an uptrend during flower development, whereas the downstream genes *DFR-1*, *5GT-1* and *ANS-1* were continuously reduced with flower development. The *AOMT-1* transcript levels were complex, with an uptrend in PO and downward trend in PR and RO during flower development. These results demonstrated that the expression patterns of the twelve selected DEGs analyzed by qRT-PCR were in agreement with the expression patterns obtained by Illumina sequencing (Figure 9).

## 3. Discussion

*P. rockii* has large, deep purple variegations at the base of its petals and contributes to variegation patterns in tree peony cultivars, which exhibit very high ornamental and commercial values. However, all F1 progeny derived from crossing between *P. rockii* and the other tree peony wild species (*P. ostii*) with pure white petals exhibited variegation at the base of their petals, suggesting that the variegation of *P. rockii* exhibited dominant inheritance.

A comparative transcriptomic analysis among the petals of PR, PO and RO individuals was conducted using Illumina sequencing. A total of 181,866 unigenes were assembled (Table S1), of which 70,944 unigenes were annotated in various databases. Pathway analysis of the DEGs indicated that the three comparisons shared 100 KEGG pathways, including phenylpropanoid biosynthesis, flavonoid biosynthesis and anthocyanin biosynthesis. The phenylpropanoid biosynthesis and flavone and flavonol biosynthesis pathways were abundant in PO, flavonoid biosynthesis showed a high abundance in PO and RO, and anthocyanin biosynthesis was relatively abundant in PR. These results validated the conclusion that anthocyanin accumulation was the main contributor to variegation in PR.

The spatial distribution of different pigment compositions is a primary cause of variegation formation on the petals [4]. Petal pigments usually are primarily distributed in the upper epidermal cells but are also found in the palisade tissue and the lower epidermis of the dark colored petals [22,23,24,25]. The various shapes of petal epidermal cells also have an important impact on flower coloration [26,27,28]. Therefore, the spatial location of the pigment within the petals and the shape of the pigmented cells were examined in the PR, PO and RO flowers. The colored cells in the variegated petals were primarily located in the upper and lower epidermal layers. However, the upper epidermal cells showed much deeper color than the lower epidermal cells (Figure 2). Moreover, all of the epidermal cells between the variegated and non-variegated petals were elongated and explanate (Figure 3), suggesting that the variegation was caused by differences in the spatial location of the colored cells within the tissues.

Additionally, the pattern and color of the variegation in RO were triangular and vivid purple-red, whereas those in PR were circular and dark purple (Figure 1 and Table 1). The anthocyanin compositions were Cy3G, Cy3G5G, Pn3G and Pn3G5G in the variegation of the PR and RO petals (Table 2), which was consistent with the results reported by Zhang et al. [6]. Moreover, the variegation in PR was “Cy > Pn” phenotype, whereas the variegation in RO had the opposite phenotype (Table 2). The total anthocyanin in the variegation of PR was five times higher than that in RO, whereas the total flavonoid content was the highest in the background of PR (Table 1). These results indicated that Cy-based pigments resulted in the dark purple variegation formation in *P. rockii*, which was consistent with the results obtained by Zhang et al. [6]. These physiological results were in perfect agreement with the results obtained in the anthocyanin biosynthetic gene expression analysis. As shown in Figure 6, most members of *CHS*, *CHI*, *FLS*, *ANS*, and *3GT* genes involved in anthocyanin biosynthetic were up-regulated in PR or RO, whereas the members of *F3H*, *F3’H*, *AOMT* and *5GT* genes were up-regulated in PO. One of the three *DFR* unigenes was significantly abundant in PR and RO. Moreover, co-expression of *PsCHS*, *PsF3’H*, *PsDFR* and *PsANS* contributed to the abundant anthocyanin accumulation involved in spot formation in the tree peony ‘Jinrong’ [7]. Anthocyanins are transported to the vacuoles by GSTs, resulting in coloration [29,30]. Based on the DEG analysis, 28 DEGs were predicted to be the key genes involved in variegation formation (Table S9). Among them, *CHI-1* (c105653.graph_c0), *DFR-1* (c126380.graph_c0), *FLS-1* (c102035.graph_c1), *ANS-1* (c124882.graph_c0) and *GST-1* (c105192.graph_c0) showed significantly higher expression in PR and RO than in PO. Specifically, *DFR-1*, *ANS-1* and *GST-1* were barely expressed in PO during flower development (Figure 9). In the anthocyanin biosynthetic pathway, *CHI-1* catalyzes the isomerization of 4,2′,4′,6′-tetrahydroxychalcone to form naringenin, which is required in the metabolic branch pathways of flavone and anthocyanin synthesis. Dihydrokaempferol represents a branch point in anthocyanin biosynthesis in the production of both colorless kaempferol through *FLS-1* and cyanidin through co-expression of *DFR-1* and *ANS-1* (Figure 9). Interestingly, some members of AOMTs, which are responsible for the methylation of anthocyanins and contribute to the purple coloration in *Paeonia* plants, were expressed at higher levels in PR than in RO, whereas other members were abundantly expressed in both RO and PO (Figure 6). To the best of our knowledge, methylated peonidin, Pn3G and Pn3G5G were more abundant in PR than in RO, and some methylated colorless flavonoids, such as Is (isorhamnetin), accumulated more in PO and RO petals than in PR. Additionally, the interaction network analysis showed that *ANS* (c124882.graph_c0), *CHS* (c102425.graph_c0) and *GST* (c115951.graph_c0) exhibited the most positive correlations with other genes, including structural genes and transcription factors (Figure 8). Among them, *CHS* (c102425.graph_c0) was markedly up-regulated in all materials in the anthocyanin biosynthetic pathway and provided more substrates for anthocyanin and flavonoid biosynthesis. Therefore, we concluded that *CHS*, *DFR*, *ANS*, and *GST* were mainly responsible for variegation pigmentation in *P. rockii*.

Because the expression of the *CHS* (c102425.graph_c0), *DFR* (c126380.graph_c0), *ANS* (c124882.graph_c0) and *GST* (c115951.graph_c0) were coordinately up-regulated in PR and RO, the transcriptional regulation of these structural genes is most likely to influence the variegation formation. In higher plants, the R2R3-MYB, bHLH, and WD40 transcription factors, especially the R2R3-MYBs, are the primary regulatory genes that influence the anthocyanin biosynthesis intensity and patterns to form different variegation patterns [21]. *LhMYB12* is responsible for the coordinated expression of *LhCHSa, LhCHSb, LhF3H, LhF3’H, LhDFR* and *LhANS* and controls the splatter-type tepal spots in Asiatic hybrid lilies [4,31] and *PeMYB11* activates the expression of anthocyanin biosynthetic genes *PeF3H5*, *PeDFR1* and *PeANS3*, and results in the red spot in the callus in *Phalaenopsis* spp. [12]. Correspondingly, in the present work, we detected 14 DEGs encoding R2R3-MYBs and 4 DEGs encoding bHLHs (Table S9). Among them, c131300.graph_c0, c133735.graph_c0 and c115179.graph_c0 exhibited the most positive correlations with other genes, including other *R2R3-MYB* unigenes and *GST*, *ANS*, *CHS*, *AOMT* and *bHLH* unigenes (Figure 8). Moreover, c133735.graph_c0 and c131300.graph_c0 were both annotated as anthocyanin regulatory C1 protein [32,33] and showed significantly higher expression in PR and RO petals than in PO. However, another R2R3-MYB DEG (c119993.graph_c0), which was also annotated as anthocyanin regulatory protein and up-regulated in PO, interacted with c133128.graph_c0 (*PAL*), c114770.graph_c0 (*AOMT*) and other *GST* unigenes that up-regulated in PO. Thus, c131300, graph_c0 and c133735.graph_c0 were more likely to regulate the biosynthesis of anhocyanins in *P. rockii* petals. To sum up, based on our results we speculated that two R2R3-MYB genes (c131300.graph_c0 and c133735.graph_c0 ) acted as hub genes to regulate variegation formation by triggering or coordinating other coloration-associated genes, such as *CHS*, *ANS*, and *GST*, in PR. Hence, whether these two R2R3-MYB transcription factors function with bHLH transcription factors and how to influence the spatial formation of variegation in *P. rockii*, and the up- and down factors associated with these two R2R3-MYB transcription factor function should be further investigated. Fortunately, the large number of gene sequences produced by petal transcriptome generated by our work will accelerate such investigations.

## 4. Materials and Methods

### 4.1. Plant Material

*P. rockii* (PR) and *P. ostii* (PO) were grown in the germplasm repository of the College of Landscape Architecture and Art, Northwest A&F University, Shaanxi, China. To analyze the segregation of the variegation characteristic, PR was crossed with PO, and their F1 progeny (RO) were grown under the same conditions (Figure 1). All of the ground plants grew well with sufficient light and water supply. For the transcriptome sequencing, petal samples were separately collected at five different opening stages from the end of April to early May in 2015. The flower opening stages were described by Shi et al. [15] as follows: stage 1, unpigmented tight bud; stage 2, slightly pigmented soft bud; stage 3, initially opened flower; stage 4, half opened flower; and stage 5, fully opened and pigmented flower with exposed anthers. To measure the pigment contents, the variegation and background in each petal at stage 5 were separated and pooled. The samples were immediately frozen in liquid nitrogen and stored at −80°C prior to analysis. Additionally, the stage 5 petals were separately detached and used immediately for the morphological and anatomical observation.

### 4.2. Petal Color Variable Measurements

The variegation and background colors of all samples at stage 5 were compared with the Royal Horticultural Society Color Chart (RHSCC) and then measured with a chroma meter (CR-400, Konica Minolta Sensing, Inc., Osaka, Japan) using three color parameters (the L*, a* and b* values). In these measurements, L* represents lightness ranging from black (L* = 0) to white (L* = 100), a* represents the balance between red and green, b* represents the ratio of yellow and blue, and C* represents the saturation of the color [6]. Five replicates were recorded [22,34].

### 4.3. Microscopic Observation of the Epidermal Cells and Transverse Sections

Fresh petals with variegation and background from all samples at stage 5 were cross-sectioned. The upper and lower epidermal layers were peeled off using a razor blade. The layers were placed onto a glass slide with a drop of water and then immediately observed under a light microscope (BX43, Olympus, Tokyo, Japan) equipped with a DS cooled camera head with the FNIS-Elements image processing software.

### 4.4. Scanning Electron Microscopy (SEM)

To identify the cell shapes associated with upper and lower variegation and background in the petals from all samples, SEM was performed according to the method of Qi et al. [22].

### 4.5. Pigment Measurement

Pigment extraction was conducted according to the method of Hashimoto et al. [35]. The extracts were filtered through a membrane filter (0.45 μm) for HPLC analysis. The pigment composition was determined using HPLC-ESI-MSn (LCQ Deca XP MAX, Thermo Scientific, Waltham, MA, USA) according to the method of Zhang et al. [6]. The anthocyanin and flavonoid contents were measured semi-quantitatively based on simple linear regression using malvidin-3, 5-di-oglucoside (Mv3G5G) and rutin as the standards at 520 nm and 350 nm, respectively. The anthocyanin and flavonoid contents were calculated in milligrams per gram fresh weight (as a quantity of Mv3G5G in mg/g and a quantity of rutin in mg/g, respectively). The mean values and standard deviations (SDs) were gained from three biological replicates. The CI (co-pigmentation index) was calculated by dividing TF (total flavone and flavonol in mg/100 mg dry petals) by TA (total anthocyanidin mg/100 mg dry petals) (i.e., CI = TF/TA) [6].

### 4.6. RNA Extraction, Library Construction, and RNA-seq

For each biological replicate of PR, PO and PR, an equal amount of petals at five different opening stages was pooled, respectively. Total RNA was extracted from 1-mg pooled samples of petals from PR, PO and PR obtained by homogenizer (TissueLyzer; Qiagen, Valencia, CA, USA) using the Quick RNA Isolation Kit (Bioteke Corporation, Beijing, China) according to the manufacturer’s protocols. The RNA purity was examined by using the NanoDrop 2000 spectrophotometer (Thermo Scientific, Waltham, MA, USA) and 1% agarose gel electrophoresis. The RNA integrity was checked using an Agilent 2100 Bioanalyzer (Santa Clara, CA, USA) with an RIN (RNA integrity number) > 8.0. The Bioanalyzer results of all RNA samples were listed in Table S10. 6-μg total RNA of three biological replicates from PR, PO and PR were used for library construction. The library construction and the RNA-seq analysis were performed by the Biomarker Biotechnology Corporation (Beijing, China) using an Illumina HiSeq^TM^ 2500 platform. All sequencing data were deposited in the National Center for Biotechnology Information (NCBI) Sequence Read Archive with accession number SRP109687.

### 4.7. De Novo Assembly and Annotation

After excluding poor quality reads with adaptors, ambiguous nucleotides and low quality, clean reads were obtained from the raw reads and then assembled *de novo* using the Trinity software (Windows v3.0) (http://trinityrnaseq.sourceforge.net/) with K-mer = 25 and group pairs distance = 300 [36]. For the functional annotation, the resulting unigenes were aligned to a series of public databases, including such as NCBI NR, Pfam, SwissProt database, KEGG database, GO and COG and KOG databases using Blastx (*e*-value < 0.00001).

### 4.8. Differential Gene Expression Analysis

The expression levels of unigenes were calculated using the FPKM (fragment per kilobase per million mapped reads) method [37]. Then, edgeR (Windows v3.0) (http://bioconductor.org/packages/release/bioc/html/edgeR.html) was used to normalize the expression level of each unigene in the three samples to identify the differentially expressed genes by pairwise comparisons. The false discovery rate (FDR) control and the ratio of FPKM values from two samples were used to compute significant differences in the gene expression levels. Here, thresholds of an FDR ≤ 0.001 and an absolute value of log2 ratio ≥ 2 were used to judge the significance of the observed differential gene expression. Additionally, the DEGs were annotated and categorized automatically using the GO and KEGG databases to screen for DEGs involved in petal variegation formation. To analyze variegated pigment interactions, a network of all differentially expressed pigmentation-related genes was constructed using the R package “weighted correlation network analysis” [38] according to the protocol [39]. Two nodes were determined to be connected if the absolute value of the Pearson correlation coefficient >0.93 [40]. Then the networks were visualized using Cytoscape (v.3.1.0) [41].

### 4.9. qRT-PCR Analysis

Twelve variegation-related genes were subjected to qRT-PCR on the ABI 7500 Sequence Detector (Applied Biosystems, Foster, CA, USA) using a SYBR Premix Ex Taq Kit (Takara; Otsu, Japan) according to the manufacturer’s protocol. Specific primers were designed using the Primer Premier software (Table S11). The cDNA synthesis and qRT-PCR were performed as described previously [17]. The relative expression levels of genes in the petals at the different opening stages were normalized to the *TUB* gene expression level in the same sample and calibrated to the transcription level in the PR petals at stage 1. All reactions were conducted in three biological replicates and three technical replicates. A two-sided *t* test was used to compare the expression levels.

## Figures and Tables

**Table 1:** The Flower color, TA, TF and CI of the background and variegation of materials at fully open stage.

Materials	Parts	Color Scheme	CIE L* a* b* Color Coordinates				TA ^b^	TF ^b^	CI ^b^
			L* ^a^	a* ^a^	b* ^a^	C* ^a^			
PO ^c^	B ^d^	White	77.51 ± 0.988	−8.18 ± 0.933	6.45 ± 1.001	10.25 ± 0.114	0	44.41 ± 1.002	∞
PR ^c^	V ^d^	Dark purple	19.37 ± 0.546	−1.35 ± 0.221	0.96 ± 0.116	1.65 ± 0.065	43.9 ± 0.532	16.36 ± 0.423	0.37
	B	White	81.2 ± 0.541	−9 ± 0.311	7.8 ± 0.135	11.91 ± 0.214	0	31.66 ± 0.358	∞
RO ^c^	V	Purple-red	33.53 ± 0.621	35.03 ± 0.081	−7.1 ± 0.952	35.75 ± 1.021	8.36 ± 0.975	12.83 ± 0.896	1.53
	B	White	82.84 ± 0.465	−9.68 ± 0.389	8.76 ± 0.552	13.17 ± 0.485	0	28.86 ± 0.493	∞

^a^ L*, lightness; a*, b*: chromatic components; C*, chroma (brightness). ^b^ TF: Total anthocyanins (mg/100 mg dry petals); TF: Total flavones and flavonols (mg/100 mg dry petals); CI, copigment index = TF/TA.

**Table 2:** The contents of anthocyanins in variegation of *P. rockii* and F1 progeny at fully open stage.

Materials	Cy3G5G ^a^ (mg/g)	Cy3G ^a^ (mg/g)	Pn3G5G ^a^ (mg/g)	Pn3G ^a^ (mg/g)
Variegation of PR	6.51 ± 0.050	28.36 ± 0.063	5.21 ± 0.115	3.82 ± 0.125
Variegation of RO	2.51 ± 0.011	0.72 ± 0.024	4.27 ± 0.046	0.87 ± 0.098

^a^ Cy3G, cyanidin 3-*O*-glucoside; Cy3G5G, cyaniding 3,5-di-*O*-glucoside; Pn3G, peonidin 3-*O*-glucoside; Pn3G5G, peonidin 3,5-di-*O*-glucoside.

**Table 3:** The contents of glycosides of flavone and flavonol in the backgroud and variegation of materials at fully open stage.

Compound (mg/g)	B ^b^ of PO	V ^b^ of PR	B of PR	V of RO	B of RO
Qu ^a^ 3,7-di-*O*-glucoside	-	0.95 ± 0.021	-	1.16 ± 0.035	2.39 ± 0.039
Km ^a^ 3,7-di-*O*-glucoside	8.51 ± 0.110	2.25 ± 0.034	5.52 ± 0.10	1.38 ± 0.031	4.33 ± 0.097
Is ^a^ 3,7-di-*O*-glucoside	12.11 ± 0.089	1.69 ± 0.037	0.0 ± 0.012	3.41 ± 0.055	4.54 ± 0.072
Qu ^a^ 3-*O*-glucoside	-	2.16 ± 0.036	-	2.1 ± 0.026	0.31 ± 0.011
Lu ^a^ 7-*O*-glucoside	0.05 ± 0.008	3.08 ± 0.010	0.24 ± 0.009	2.42 ± 0.085	6.01 ± 0.154
Ap ^a^ 7-*O*-glucoside	9.34 ± 0.132	3.64 ± 0.062	15.01 ± 0.135	0.35 ± 0.065	4.18 ± 0.426
Is ^a^ 3-*O*-glucoside	3.45 ± 0.054	1.01 ± 0.055	-	1.26 ± 0.058	0.14 ± 0.035
Ap ^a^ 7-*O*-neohesperidoside	10.95 ± 0.113	1.58 ± 0.053	10.82 ± 0.110	0.75 ± 0.031	6.96 ± 0.082

^a^ Qu, quercetin; Km, kaempferol; Is, isorhamnetin; Lu, luteolin; Ap, apigenin. ^b^ B, the background part of petals; V, the variegation part of petals.

## Supplementary Materials

Supplementary Materials are available online.

## Abbreviations

3GT	anthocyanin 3-*O*-glucosyltransferase
5GT	anthocyanin 5-*O*-glucosyltransferase
a*, b*	chromatic components
ANS	anthocyanin synthase
AOMT	anthocyanin *O*-methyltransferase
Ap	apigenin
bHLH	basic helix-loop-helix
C*	chroma (brightness)
CHI	chalcone isomerase
CHS	chalcone synthase
CI	Co-pigment index
COG	Cluster of orthologous groups of proteins database
Cy3G	cyanidin 3-*O*-glucoside
Cy3G5G	cyaniding 3,5-di-*O*-glucoside
DEGs	differentially expressed genes
DFR	dihydroflavonol 4-reductase
F3’H	flavonoid 3’-hydroxylase
F3H	flavanone 3-hydroxylase
FDR	false discovery rates
FLS	flavonol synthase
FPKM	fragments per kilobase per million mapped reads
GO	gene ontology
GST	glutathione S-transferase
HPLC	hogh-performance liquid chromatography
Is	isorhamnetin
KEGG	Kyoto encyclopedia of genes and genomes database
Km	kaempferol
KOG	euKaryotic orthologous groups
L*	lightness
Lu	luteolin
Nr	Non-resundant protein database
PAL	phenylalanine ammonia-lyase
Pfam	protein family
Pn3G	peonidin 3-*O*-glucoside
Pn3G5G	peonidin 3,5-di-*O*-glucoside
qRT-PCR	quantitative reverse transcription PCR
Qu	quercetin
R	pearson’s correlation coefficient
RHSCC	Royal horticultural society colour chart
SwissProt	SwissProt protein database
TA	total anthocyanin
TF	total flavone and flavonol in mg/100 mg dry petals.
