# Supplementary material for: Biochemical and Comparative Transcriptomic Analyses Identify Candidate Genes Related to Variegation Formation in Paeonia rockii
Source: Molecules. 2017 Aug 17;22(8):1364. doi: 10.3390/molecules22081364 (PMC6152351; doi:10.3390/molecules22081364)
Supplement: Supplementary file 1 [file molecules-22-01364-s001.pdf]

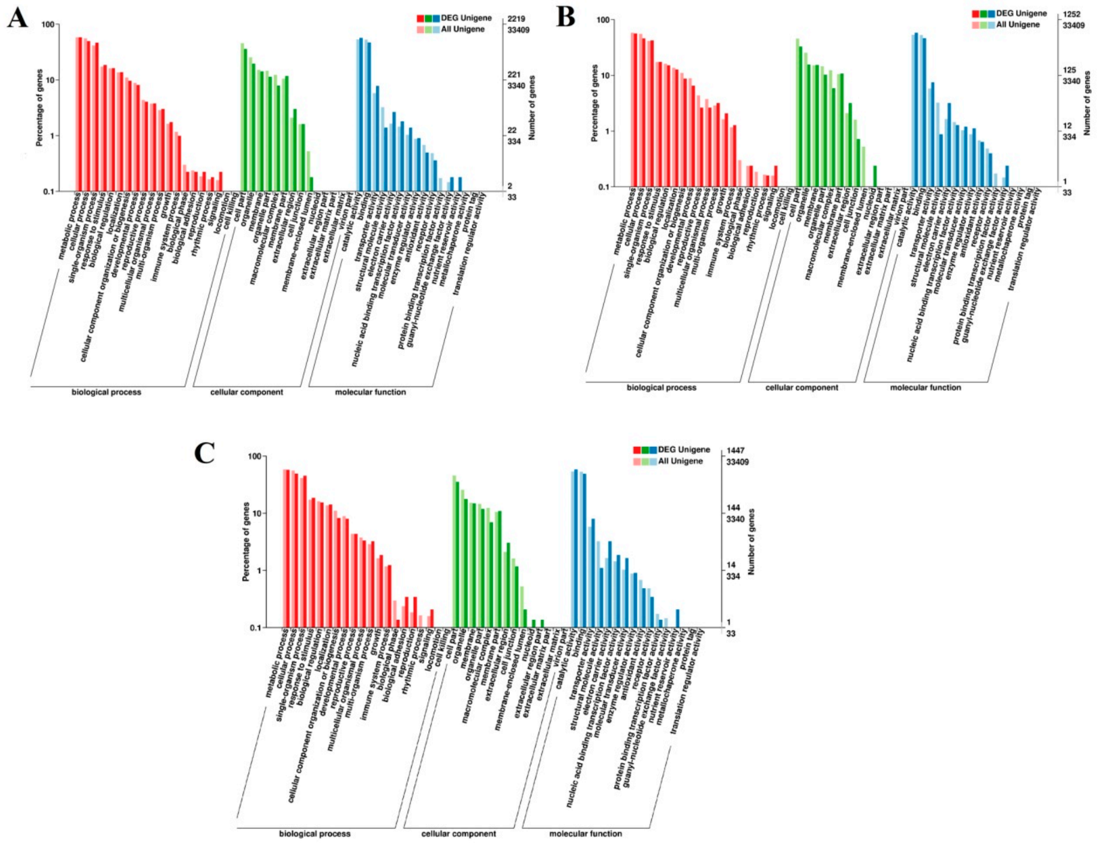

Figure S1. GO enrichment analysis of DEGs in the three groups. (A) GO enrichment analysis of DEGs for the PO and PR comparison; (B) GO enrichment analysis of DEGs for the PR and RO comparison; (C) GO enrichment analysis of DEGs for the PO and RO comparison.

Table S1. Statistics of functional annotation.

Annotated Database	Annotated_Number	300<=length<1000	length>=1000
nr Annotation	57913 (31.84%)	23699 (33.41%)	16502 (23.26%)
Pfam Annotation	43307 (23.81%)	18082 (25.49%)	14968 (21.10%)
Swissprot Annotation	34125 (18.76%)	13910 (19.61%)	11720 (16.52%）
COG Annotation	18986 (10.44%)	7386 (10.41%)	6848（9.65%）
GO Annotation	33409 (18.37%)	13150 (18.54%)	10150 （14.31%）
KEGG Annotation	24154 (13.28%)	10433 (14.71%)	7102 （10.01%）
KOG Annotation	47410 (26.07%)	20239 (28.53%)	11436 （16.12%）
All Annotated	70944 (39.01%)	29535 (41.63%)	17361 （24.47%）
All Unigenes	181866 (100.00%）		

Table S2. KEGG pathway classification of Unigenes.

#Pathway	ko_ID	Unigene	gene	Unigene_all	gene_all		
#KEGG_n	715	KEGG_N	13897				
Starch and sucrose metabolism	ko00500	51	469	715	13897	c105513.graph_c1;c106004.graph_c0;c108042.graph_c0;c108790.graph_c0;c109140.graph_c0;c109671.graph_c0;c113248.graph_c0;c115504.graph_c0;c115711.graph_c0;c116417.graph_c0;c117822.graph_c0;c117851.graph_c0;c118665.graph_c0;c118816.graph_c0;c119325.graph_c0;c120249.graph_c0;c120576.graph_c0;c120604.graph_c0;c121742.graph_c0;c121995.graph_c0;c122721.graph_c0;c123210.graph_c0;c123220.graph_c0;c124410.graph_c0;c125155.graph_c1;c125781.graph_c0;c126509.graph_c1;c126580.graph_c0;c126605.graph_c0;c126811.graph_c0;c126934.graph_c0;c127309.graph_c0;c127792.graph_c0;c128185.graph_c0;c128547.graph_c0;c128547.graph_c1;c129333.graph_c0;c129750.graph_c0;c130060.graph_c0;c130068.graph_c0;c130187.graph_c0;c130477.graph_c0;c130684.graph_c0;c131646.graph_c1;c131799.graph_c0;c132238.graph_c0;c132359.graph_c1;c47395.graph_c0;c71123.graph_c0;c88845.graph_c0;c89200.graph_c0	K01188+K01188+K18447+K01188+K00844+K05350+K00012+K01177+K01176+K01051+K01051+K01051+K00847+K01188+K01184+K00844+K01051+K01188+K01051+K01188+K01835+K05350+K01188+K01051+K01176+K01051+K00012+K01177+K00975+K01193+K01187+K05349+K01188+K00705+K01188+K01188+K01193+K01051+K01087+K00688+K00847+K00688+K01193+K00688+K01176+K00696+K01051+K00847+K01051+K05350+K01188

Plant hormone signal transduction	ko04075	43	403	715	13897	c102167.graph_c0;c104874.graph_c0;c105109.graph_c0;c105815.graph_c0;c106087.graph_c0;c107593.graph_c0;c108427.graph_c0;c109272.graph_c0;c110148.graph_c0;c112138.graph_c0;c112179.graph_c0;c112371.graph_c0;c112578.graph_c0;c113017.graph_c0;c114656.graph_c0;c116573.graph_c0;c117033.graph_c0;c117096.graph_c0;c119187.graph_c0;c120105.graph_c1;c120546.graph_c0;c121149.graph_c0;c121691.graph_c0;c121961.graph_c0;c122051.graph_c0;c122394.graph_c0;c122713.graph_c0;c123048.graph_c0;c124285.graph_c0;c124691.graph_c0;c124750.graph_c1;c124826.graph_c0;c127036.graph_c1;c127278.graph_c0;c127580.graph_c0;c127695.graph_c0;c128432.graph_c0;c130168.graph_c0;c131976.graph_c0;c132781.graph_c0;c132990.graph_c0;c88326.graph_c0;c90004.graph_c0	K14488+K14488+K14497+K14505+K14490+K14492+K14490+K14492+K14490+K13464+K14488+K14488+K14492+K14484+K13946+K14505+K14484+K14496+K13946+K14488+K14488+K14488+K14497+K14490+K13464+K14484+K14492+K14505+K14491+K14509+K14490+K14512+K14491+K14489+K14488+K14505+K14494+K14509+K14515+K14492+K14509+K14488+K14496
Phenylpropanoid biosynthesis	ko00940	39	250	715	13897	c101742.graph_c0;c101742.graph_c1;c105069.graph_c0;c105513.graph_c1;c106004.graph_c0;c107475.graph_c0;c108790.graph_c0;c109671.graph_c0;c111410.graph_c0;c111764.graph_c0;c114770.graph_c0;c116105.graph_c0;c116628.graph_c0;c117640.graph_c0;c118247.graph_c0;c118816.graph_c0;c119082.graph_c0;c120064.graph_c0;c120064.graph_c1;c120604.graph	K00083+K00083+K00430+K01188+K01188+K00430+K01188+K05350+K13065+K11188+K13066+K13065+K00430+K12356+K00430+K01188+K01904+K13066+K13066+K01188+K00430+K01188+K05350+K01188+K01904+K00430+K05349+K01188+K00083+K01188+K01188+K00430+K10775+K

						_c0;c121941.graph_c0;c121995.graph_c0;c123210.graph_c0;c123220.graph_c0;c125412.graph_c0;c125906.graph_c0;c127309.graph_c0;c127792.graph_c0;c128306.graph_c0;c128547.graph_c0;c128547.graph_c1;c130735.graph_c0;c133129.graph_c0;c133930.graph_c0;c50245.graph_c0;c86972.graph_c0;c88845.graph_c0;c89173.graph_c0;c89200.graph_c0	00430+K00083+K11188+K05350+K09755+K01188
Carbon metabolism	ko01200	37	955	715	13897	c100613.graph_c0;c102566.graph_c0;c105373.graph_c0;c106243.graph_c0;c109140.graph_c0;c110055.graph_c0;c110315.graph_c0;c112914.graph_c0;c114199.graph_c0;c118522.graph_c0;c118802.graph_c0;c118938.graph_c0;c119204.graph_c0;c119582.graph_c1;c120124.graph_c0;c120249.graph_c0;c120317.graph_c0;c124334.graph_c0;c124752.graph_c0;c126079.graph_c0;c126578.graph_c0;c128281.graph_c0;c128366.graph_c0;c129490.graph_c0;c130262.graph_c0;c132024.graph_c0;c132202.graph_c0;c134376.graph_c0;c134598.graph_c0;c134680.graph_c0;c50718.graph_c0;c51688.graph_c0;c85077.graph_c0;c88872.graph_c0;c88948.graph_c0;c90012.graph_c0;c91675.graph_c0	K00025+K00122+K00161+K00134+K00844+K01602+K00830+K00134+K01100+K00382+K00134+K05605+K05298+K01057+K01057+K00844+K01057+K01738+K00164+K00873+K00121+K01610+K01595+K00026+K13034+K01738+K02160+K01963+K01738+K00161+K01057+K01057+K00627+K01679+K13034+K01962+K00161
Ribosome	ko03010	36	996	715	13897	c100604.graph_c0;c102269.graph_c0;c102533.graph_c0;c106833.graph_c0;c107031.graph_c0;c108526.graph_c1;c109164.graph_c0;c110436	K02977+K02876+K02937+K02976+K02930+K02979+K02873+K02870+K02909+K02922+K02942+K02879+K02

						.graph_c0;c113079.graph_c0;c114175.graph_c0;c117479.graph_c0;c118513.graph_c0;c119820.graph_c0;c120768.graph_c0;c123949.graph_c0;c124097.graph_c0;c124830.graph_c0;c125171.graph_c0;c125852.graph_c0;c125958.graph_c0;c129136.graph_c0;c130486.graph_c0;c131743.graph_c0;c132081.graph_c0;c132355.graph_c0;c133030.graph_c0;c133694.graph_c0;c133984.graph_c0;c134599.graph_c0;c49761.graph_c0;c55594.graph_c0;c74451.graph_c0;c77479.graph_c0;c86145.graph_c0;c90571.graph_c0;c92625.graph_c0	876+K02941+K02932+K02988+K02896+K02937+K02896+K02993+K02938+K02882+K02938+K02964+K02980+K02937+K02882+K02868+K02960+K02938+K02960+K02949+K02960+K02983+K02949+K02933
Protein processing in endoplasmic reticulum	ko04141	35	868	715	13897	c102059.graph_c0;c102180.graph_c0;c106546.graph_c0;c108317.graph_c0;c108531.graph_c0;c110519.graph_c0;c110723.graph_c0;c111425.graph_c0;c115661.graph_c0;c115728.graph_c0;c116596.graph_c0;c118015.graph_c0;c120059.graph_c0;c122224.graph_c0;c122836.graph_c0;c123030.graph_c0;c123347.graph_c0;c124083.graph_c0;c125391.graph_c1;c125404.graph_c0;c129495.graph_c0;c131510.graph_c0;c131834.graph_c0;c131854.graph_c1;c132610.graph_c0;c133097.graph_c0;c134065.graph_c0;c23599.graph_c0;c23697.graph_c0;c51615.graph_c0;c52034.graph_c0;c71721.graph_c0;c71733.	K13993+K03094+K07342+K03283+K03094+K03283+K13993+K13993+K13993+K03094+K13993+K13993+K03237+K13993+K13993+K09503+K03094+K09503+K13993+K10575+K08852+K03283+K03283+K03283+K09503+K13993+K16196+K03283+K03283+K03868+K13993+K13993+K04079+K13993+K03868

Plant-pathogen interaction	ko04626	30	373	715	13897	graph_c0;c90083.graph_c0;c97655.graph_c0 c100506.graph_c0;c108372.graph_c0;c108852.graph_c0;c112144.graph_c0;c114370.graph_c0;c115120.graph_c0;c118619.graph_c0;c118881.graph_c0;c119307.graph_c0;c119888.graph_c0;c121838.graph_c0;c122060.graph_c0;c125696.graph_c0;c125898.graph_c1;c127934.graph_c0;c128760.graph_c0;c129591.graph_c0;c130786.graph_c0;c131652.graph_c1;c132003.graph_c0;c132441.graph_c0;c132784.graph_c0;c132982.graph_c1;c133874.graph_c0;c133916.graph_c0;c134003.graph_c0;c134348.graph_c0;c50350.graph_c0;c71733.graph_c0;c91463.graph_c0	K13448+K13448+K13412+K02183+K04368+K13448+K13448+K13448+K13412+K13412+K13459+K05391+K13459+K13457+K13420+K13412+K05391+K13429+K13420+K18835+K13412+K13447+K13447+K13412+K13448+K05391+K13457+K13448+K04079+K12795
Biosynthesis of amino acids	ko01230	30	812	715	13897	c100821.graph_c0;c102878.graph_c0;c106243.graph_c0;c112408.graph_c0;c112914.graph_c0;c114056.graph_c0;c117589.graph_c0;c118697.graph_c0;c118802.graph_c0;c121031.graph_c0;c121090.graph_c0;c122143.graph_c0;c123498.graph_c0;c123819.graph_c0;c123986.graph_c0;c124334.graph_c0;c124635.graph_c0;c125956.graph_c0;c126079.graph_c0;c130262.graph_c0;c132013.graph_c0;c132024.graph_c0;c133161.graph_c0;c133226.graph_c0;c134598.graph_c0;c70627.graph_c0;c71676.graph_c0;c8894	K00891+K15227+K00134+K13832+K00134+K10206+K00620+K00611+K00134+K01817+K15227+K00052+K00215+K00826+K01626+K01738+K01915+K15227+K00873+K13034+K00815+K01738+K13832+K13832+K01738+K01778+K15227+K13034+K01704+K00891

						8.graph_c0;c93628.graph_c0;c95528.graph_c0	
Glycolysis / Gluconeogenesis	ko00010	27	448	715	13897	c102004.graph_c0;c105373.graph_c0;c106243.graph_c0;c108564.graph_c0;c109130.graph_c0;c109140.graph_c0;c109581.graph_c0;c111937.graph_c0;c112914.graph_c0;c118522.graph_c0;c118802.graph_c0;c120249.graph_c0;c122721.graph_c0;c122868.graph_c0;c126079.graph_c0;c126467.graph_c0;c126578.graph_c0;c128281.graph_c0;c128735.graph_c0;c130417.graph_c0;c130417.graph_c1;c132004.graph_c0;c132004.graph_c1;c134543.graph_c0;c134680.graph_c0;c85077.graph_c0;c91675.graph_c0	K18857+K00161+K00134+K00001+K01792+K00844+K18857+K01785+K00134+K00382+K00134+K00844+K01835+K00128+K00873+K18857+K00121+K01610+K00128+K00128+K00128+K01785+K01785+K01568+K00161+K00627+K00161
Pentose and glucuronate interconversions	ko00040	26	202	715	13897	c102361.graph_c0;c102552.graph_c0;c110588.graph_c0;c112781.graph_c0;c113248.graph_c0;c116417.graph_c0;c116832.graph_c0;c117822.graph_c0;c117851.graph_c0;c119325.graph_c0;c120576.graph_c0;c121742.graph_c0;c122856.graph_c0;c122868.graph_c0;c123853.graph_c0;c124410.graph_c0;c124721.graph_c0;c124896.graph_c0;c125781.graph_c0;c126509.graph_c1;c128735.graph_c0;c129750.graph_c0;c130417.graph_c0;c130417.graph_c1;c132359.graph_c1;c71123.graph_c0	K01728+K01728+K01728+K01728+K00012+K01051+K00008+K01051+K01051+K01184+K01051+K01051+K00011+K00128+K01728+K01051+K01728+K01728+K01051+K00012+K00128+K01051+K00128+K00128+K01051+K01051

Oxidative phosphorylation	ko00190	24	552	715	13897	c100128.graph_c0;c105010.graph_c0;c107179.graph_c0;c107242.graph_c0;c110866.graph_c0;c112664.graph_c0;c120026.graph_c0;c121921.graph_c0;c122261.graph_c0;c122529.graph_c0;c123754.graph_c0;c124122.graph_c0;c125492.graph_c0;c125750.graph_c0;c126976.graph_c0;c127162.graph_c0;c129853.graph_c0;c132160.graph_c0;c133150.graph_c1;c28152.graph_c0;c50116.graph_c0;c70250.graph_c0;c71577.graph_c0;c96666.graph_c0	K02115+K02152+K02115+K00411+K01535+K01535+K05577+K02150+K01535+K02109+K01535+K00417+K02109+K05573+K02113+K02256+K01507+K03881+K03935+K01535+K01535+K02267+K01535+K01507
Amino sugar and nucleotide sugar metabolism	ko00520	24	335	715	13897	c106554.graph_c0;c106993.graph_c0;c107495.graph_c0;c109140.graph_c0;c113248.graph_c0;c117108.graph_c0;c117257.graph_c0;c118665.graph_c0;c119330.graph_c0;c120249.graph_c0;c121484.graph_c1;c122721.graph_c0;c123062.graph_c0;c124235.graph_c0;c124607.graph_c0;c126509.graph_c1;c126605.graph_c0;c130187.graph_c0;c130464.graph_c0;c134297.graph_c0;c47395.graph_c0;c54775.graph_c0;c88590.graph_c0;c89832.graph_c0	K01183+K00326+K00621+K00844+K00012+K12448+K10046+K00847+K01183+K00844+K01836+K01835+K01784+K01784+K01209+K00012+K00975+K00847+K00966+K01183+K00847+K01784+K01784+K12449
Glutathione metabolism	ko00480	23	235	715	13897	c101895.graph_c0;c105192.graph_c0;c105273.graph_c0;c105277.graph_c0;c108187.graph_c0;c110950.graph_c0;c112220.graph_c0;c112965.graph_c0;c113610.graph_c0;c116462.graph_c0;c117370.graph_c0;c119814.graph_c0;c125111.graph_c0;c126983.graph_c0;c127844.graph_	K00799+K00799+K01919+K00799+K00432+K10808+K00799+K00799+K00799+K00799+K00799+K00799+K00799+K10807+K18592+K10807+K10808+K01581+K01919+K00799+K00799+K00799+K00799

						c0;c128678.graph_c0;c131878.graph_c0;c132914.graph_c0;c133973.graph_c0;c51066.graph_c0;c71042.graph_c0;c96247.graph_c0;c98149.graph_c0	
Purine metabolism	ko00230	21	563	715	13897	c108042.graph_c0;c110950.graph_c0;c112573.graph_c0;c115938.graph_c0;c119983.graph_c0;c119994.graph_c0;c120834.graph_c0;c121893.graph_c0;c122370.graph_c0;c122721.graph_c0;c124206.graph_c0;c126079.graph_c0;c126983.graph_c0;c128678.graph_c0;c129051.graph_c0;c130274.graph_c0;c130689.graph_c1;c131570.graph_c0;c131878.graph_c0;c134357.graph_c0;c99075.graph_c0	K18447+K10808+K03013+K03008+K03019+K03026+K01514+K01081+K00759+K01835+K03014+K00873+K10807+K10807+K02325+K01427+K00939+K01923+K10808+K02320+K00088
Spliceosome	ko03040	20	868	715	13897	c100425.graph_c0;c102019.graph_c0;c104836.graph_c0;c108164.graph_c0;c108317.graph_c0;c110519.graph_c0;c118765.graph_c0;c120921.graph_c0;c125321.graph_c0;c130121.graph_c0;c131510.graph_c0;c131834.graph_c0;c131854.graph_c1;c132365.graph_c0;c133691.graph_c0;c134621.graph_c0;c23599.graph_c0;c23697.graph_c0;c71357.graph_c0;c98951.graph_c0	K12874+K12882+K12830+K12874+K03283+K03283+K12662+K12874+K12858+K12830+K03283+K03283+K03283+K11097+K12833+K12882+K03283+K03283+K12833+K12813
Galactose metabolism	ko00052	19	166	715	13897	c102094.graph_c0;c109140.graph_c0;c111937.graph_c0;c120249.graph_c0;c121255.graph_c1;c122721.graph_c0;c122856.graph_c0;c123062.graph_c0;c124235.graph_c0;c125626.graph_c0;c126811.graph_c0;c126934.graph_c0;c12933	K18819+K00844+K01785+K00844+K06617+K01835+K00011+K01784+K01784+K07407+K01193+K01187+K01193+K01193+K01785+K01785+K06617+K01784+K01784

						3.graph_c0;c130684.graph_c0;c132004.graph_c0;c132004.graph_c1;c49856.graph_c0;c54775.graph_c0;c88590.graph_c0	
Cyanoamino acid metabolism	ko00460	18	114	715	13897	c105513.graph_c1;c106004.graph_c0;c108790.graph_c0;c109671.graph_c0;c118816.graph_c0;c120604.graph_c0;c121995.graph_c0;c123210.graph_c0;c123220.graph_c0;c127309.graph_c0;c127792.graph_c0;c127844.graph_c0;c128547.graph_c0;c128547.graph_c1;c130262.graph_c0;c88845.graph_c0;c88948.graph_c0;c89200.graph_c0	K01188+K01188+K01188+K05350+K01188+K01188+K01188+K05350+K01188+K05349+K01188+K18592+K01188+K01188+K13034+K05350+K13034+K01188
alpha-Linolenic acid metabolism	ko00592	18	130	715	13897	c102004.graph_c0;c102687.graph_c0;c109303.graph_c0;c109581.graph_c0;c115680.graph_c0;c115772.graph_c0;c118459.graph_c0;c120300.graph_c0;c121567.graph_c0;c123665.graph_c0;c124313.graph_c0;c125727.graph_c0;c126467.graph_c0;c128357.graph_c0;c129473.graph_c0;c134411.graph_c0;c134639.graph_c0;c23669.graph_c0	K18857+K00454+K08241+K18857+K10525+K01723+K01723+K05894+K01723+K10525+K10526+K08241+K18857+K10526+K00454+K00454+K00454+K00454
Phenylalanine metabolism	ko00360	18	171	715	13897	c105069.graph_c0;c107475.graph_c0;c110715.graph_c0;c111764.graph_c0;c116628.graph_c0;c118247.graph_c0;c118423.graph_c0;c119082.graph_c0;c119172.graph_c0;c121941.graph_c0;c122229.graph_c0;c125412.graph_c0;c12590	K00430+K00430+K07253+K11188+K00430+K00430+K00276+K01904+K00457+K00430+K07253+K01904+K00430+K00430+K00815+K10775+K00430+K11188

						6.graph_c0;c130735.graph_c0;c132013.graph_c0;c133129.graph_c0;c133930.graph_c0;c86972.graph_c0	
Pyruvate metabolism	ko00620	18	357	715	13897	c100613.graph_c0;c105373.graph_c0;c118522.graph_c0;c122868.graph_c0;c126079.graph_c0;c128281.graph_c0;c128366.graph_c0;c128735.graph_c0;c129490.graph_c0;c130417.graph_c0;c130417.graph_c1;c132202.graph_c0;c134376.graph_c0;c134680.graph_c0;c85077.graph_c0;c88872.graph_c0;c90012.graph_c0;c91675.graph_c0	K00025+K00161+K00382+K00128+K00873+K01610+K01595+K00128+K00026+K00128+K00128+K02160+K01963+K00161+K00627+K01679+K01962+K00161
Ubiquitin mediated proteolysis	ko04120	17	468	715	13897	c102180.graph_c0;c105963.graph_c0;c108531.graph_c0;c108920.graph_c0;c115011.graph_c0;c115728.graph_c0;c115856.graph_c0;c119129.graph_c0;c119687.graph_c0;c123347.graph_c0;c125404.graph_c0;c128987.graph_c0;c130696.graph_c0;c133559.graph_c0;c23919.graph_c0;c51615.graph_c0;c97655.graph_c0	K03094+K10144+K03094+K10144+K10144+K03094+K06688+K10583+K04506+K03094+K10575+K10581+K10581+K10144+K10573+K03868+K03868
Pyrimidine metabolism	ko00240	17	456	715	13897	c102867.graph_c0;c110950.graph_c0;c112573.graph_c0;c114780.graph_c0;c115938.graph_c0;c119983.graph_c0;c119994.graph_c0;c119999.graph_c0;c121893.graph_c0;c123592.graph_c1;c124206.graph_c0;c126983.graph_c0;c128678.graph_c0;c129051.graph_c0;c131878.graph_c0;c134357.graph_c0;c93528.graph_c0	K00857+K10808+K03013+K00876+K03008+K03019+K03026+K00761+K01081+K00857+K03014+K10807+K10807+K02325+K10808+K02320+K01520

Cysteine and methionine metabolism	ko00270	16	289	715	13897	c100613.graph_c0;c108515.graph_c0;c120855.graph_c0;c121636.graph_c0;c123728.graph_c0;c124334.graph_c0;c125581.graph_c0;c125674.graph_c0;c125789.graph_c0;c129490.graph_c0;c130262.graph_c0;c130924.graph_c0;c132013.graph_c0;c132024.graph_c0;c134598.graph_c0;c88948.graph_c0	K00025+K05933+K01761+K08963+K01762+K01738+K00547+K00547+K05933+K00026+K13034+K00558+K00815+K01738+K01738+K13034
Fatty acid metabolism	ko01212	16	268	715	13897	c100226.graph_c0;c108824.graph_c0;c111947.graph_c0;c115084.graph_c0;c117490.graph_c0;c120878.graph_c0;c121978.graph_c0;c125610.graph_c0;c127942.graph_c0;c128312.graph_c0;c128974.graph_c0;c132202.graph_c0;c132570.graph_c0;c134376.graph_c0;c71372.graph_c0;c90012.graph_c0	K09458+K01074+K10256+K10251+K10255+K10703+K10257+K10781+K10703+K09458+K01897+K02160+K00059+K01963+K01897+K01962
mRNA surveillance pathway	ko03015	16	452	715	13897	c101917.graph_c0;c101974.graph_c0;c102019.graph_c0;c106824.graph_c0;c111578.graph_c0;c118482.graph_c0;c120023.graph_c0;c122748.graph_c0;c124542.graph_c0;c126315.graph_c0;c126899.graph_c0;c127188.graph_c0;c129634.graph_c0;c132292.graph_c2;c134621.graph_c0;c97867.graph_c0	K11583+K14376+K12882+K14376+K06269+K03456+K13126+K11584+K14400+K13126+K13126+K14400+K03265+K03456+K12882+K14324
Endocytosis	ko04144	16	472	715	13897	c108317.graph_c0;c110519.graph_c0;c111700.graph_c0;c111740.graph_c0;c117146.graph_c0;c119051.graph_c0;c127088.graph_c0;c128997.graph_c0;c130583.graph_c1;c131314.graph_c0;c131510.graph_c0;c131834.graph_c0;c13185	K03283+K03283+K01115+K07904+K04646+K07897+K00889+K01115+K01115+K12483+K03283+K03283+K03283+K01115+K03283+K03283

						4.graph_c1;c133450.graph_c0;c23599.graph_c0;c23697.graph_c0	
RNA transport	ko03013	16	630	715	13897	c102019.graph_c0;c110008.graph_c0;c114119.graph_c0;c120023.graph_c0;c120059.graph_c0;c120893.graph_c0;c125991.graph_c0;c126315.graph_c0;c126899.graph_c0;c131315.graph_c0;c131752.graph_c0;c132224.graph_c2;c133101.graph_c0;c134621.graph_c0;c97352.graph_c0;c97867.graph_c0	K12882+K03251+K14298+K13126+K03237+K12160+K03231+K13126+K13126+K14312+K03250+K14288+K18213+K12882+K12160+K14324
Photosynthesis	ko00195	15	144	715	13897	c100128.graph_c0;c107088.graph_c0;c107179.graph_c0;c120141.graph_c0;c122529.graph_c0;c124921.graph_c0;c125492.graph_c0;c126976.graph_c0;c130722.graph_c0;c131924.graph_c1;c50756.graph_c0;c71236.graph_c0;c71464.graph_c0;c95758.graph_c0;c97392.graph_c0	K02115+K03542+K02115+K02707+K02109+K02694+K02109+K02113+K02710+K02693+K02721+K02695+K08905+K02636+K02717
DNA replication	ko03030	14	216	715	13897	c108872.graph_c0;c116129.graph_c0;c118291.graph_c0;c118790.graph_c0;c121683.graph_c0;c126496.graph_c0;c128517.graph_c0;c129051.graph_c0;c130511.graph_c1;c133355.graph_c0;c133907.graph_c0;c133947.graph_c0;c134357.graph_c0;c92680.graph_c0	K10745+K10755+K02541+K10740+K10745+K02212+K07466+K02325+K02542+K10747+K02210+K02209+K02320+K10740
Tyrosine metabolism	ko00350	14	121	715	13897	c102004.graph_c0;c108564.graph_c0;c109581.graph_c0;c110715.graph_c0;c118423.graph_c0;c119172.graph_c0;c122229.graph_c0;c126467.graph_c0;c126578.graph_c0;c130866.graph_c0;c131275.graph_c0;c132013.graph_c0;c23725	K18857+K00001+K18857+K07253+K00276+K00457+K07253+K18857+K00121+K01592+K00451+K00815+K01592+K01592

						.graph_c0;c23725.graph_c1	
Terpenoid backbone biosynthesis	ko00900	13	155	715	13897	c108010.graph_c1;c108150.graph_c0;c112284.graph_c0;c122587.graph_c0;c123354.graph_c0;c123699.graph_c0;c124462.graph_c0;c124505.graph_c0;c125410.graph_c0;c127716.graph_c0;c127762.graph_c1;c81933.graph_c0;c88832.graph_c0	K00787+K01662+K01641+K15889+K11778+K05955+K13789+K00869+K10960+K00587+K11778+K00021+K00021
RNA degradation	ko03018	13	419	715	13897	c109491.graph_c0;c116122.graph_c0;c116599.graph_c1;c116900.graph_c0;c120023.graph_c0;c122547.graph_c0;c126315.graph_c0;c126899.graph_c0;c127472.graph_c0;c128434.graph_c0;c130826.graph_c0;c131538.graph_c1;c134213.graph_c0	K04077+K12602+K12593+K12619+K13126+K14442+K13126+K13126+K04043+K12580+K12603+K12619+K04077
Glycerophospholipid metabolism	ko00564	12	243	715	13897	c105872.graph_c0;c111700.graph_c0;c120920.graph_c0;c121925.graph_c0;c124631.graph_c0;c127052.graph_c0;c128997.graph_c0;c130294.graph_c0;c130583.graph_c1;c133450.graph_c0;c70983.graph_c0;c93485.graph_c0	K06130+K01115+K08730+K06130+K06129+K13508+K01115+K13508+K01115+K01115+K13508+K06130
Cutin, suberine and wax biosynthesis	ko00073	12	60	715	13897	c100231.graph_c0;c115679.graph_c0;c119134.graph_c1;c124424.graph_c0;c124583.graph_c0;c125472.graph_c0;c127594.graph_c0;c131711.graph_c0;c86343.graph_c0;c88162.graph_c0;c97209.graph_c0;c99637.graph_c1	K13356+K17991+K15400+K15404+K15398+K15403+K17991+K15404+K15398+K13356+K13356+K13356
Fructose and mannose metabolism	ko00051	12	201	715	13897	c104104.graph_c0;c108042.graph_c0;c109140.graph_c0;c114913.graph_c0;c116832.graph_c0;c118665.graph_c0;c118742.graph_c0;c120249	K00895+K18447+K00844+K00895+K00008+K00847+K00895+K00844+K00011+K00847+K00966+K00847

						.graph_c0;c122856.graph_c0;c130187.graph_c0;c130464.graph_c0;c47395.graph_c0	
Arginine and proline metabolism	ko00330	12	287	715	13897	c117589.graph_c0;c118697.graph_c0;c119465.graph_c0;c122868.graph_c0;c124635.graph_c0;c126751.graph_c0;c128735.graph_c0;c129144.graph_c0;c130274.graph_c0;c130417.graph_c0;c130417.graph_c1;c132914.graph_c0	K00620+K00611+K00261+K00128+K01915+K00294+K00128+K01583+K01427+K00128+K00128+K01581
Phenylalanine, tyrosine and tryptophan biosynthesis	ko00400	12	117	715	13897	c100821.graph_c0;c102878.graph_c0;c112408.graph_c0;c121031.graph_c0;c121090.graph_c0;c123986.graph_c0;c125956.graph_c0;c132013.graph_c0;c133161.graph_c0;c133226.graph_c0;c71676.graph_c0;c95528.graph_c0	K00891+K15227+K13832+K01817+K15227+K01626+K15227+K00815+K13832+K13832+K15227+K00891
Citrate cycle (TCA cycle)	ko00020	11	304	715	13897	c100613.graph_c0;c105373.graph_c0;c118522.graph_c0;c120797.graph_c0;c124752.graph_c0;c128281.graph_c0;c129490.graph_c0;c134680.graph_c0;c85077.graph_c0;c88872.graph_c0;c91675.graph_c0	K00025+K00161+K00382+K01648+K00164+K01610+K00026+K00161+K00627+K01679+K00161
Peroxisome	ko04146	11	287	715	13897	c100231.graph_c0;c110315.graph_c0;c122610.graph_c0;c123153.graph_c0;c124505.graph_c0;c125133.graph_c0;c128974.graph_c0;c71372.graph_c0;c88162.graph_c0;c97209.graph_c0;c99637.graph_c1	K13356+K00830+K01640+K13346+K00869+K13348+K01897+K01897+K13356+K13356+K13356
Fatty acid degradation	ko00071	11	197	715	13897	c102004.graph_c0;c108564.graph_c0;c109581.graph_c0;c122868.graph_c0;c126467.graph_c0;c126578.graph_c0;c128735.graph_c0;c128974.graph_c0;c130417.graph_c0;c130417.graph_c	K18857+K00001+K18857+K00128+K18857+K00121+K00128+K01897+K00128+K00128+K01897

						1;c71372.graph_c0	
Photosynthesis - antenna proteins	ko00196	10	52	715	13897	c102634.graph_c0;c105640.graph_c0;c106848.graph_c0;c109210.graph_c0;c114287.graph_c0;c123544.graph_c0;c46372.graph_c0;c71681.graph_c0;c89853.graph_c0;c89965.graph_c0	K14172+K08917+K08909+K08912+K08908+K08912+K08913+K08913+K08908+K08913
Valine, leucine and isoleucine degradation	ko00280	10	223	715	13897	c112284.graph_c0;c118522.graph_c0;c118938.graph_c0;c122610.graph_c0;c122868.graph_c0;c123819.graph_c0;c128735.graph_c0;c130417.graph_c0;c130417.graph_c1;c97615.graph_c0	K01641+K00382+K05605+K01640+K00128+K00826+K00128+K00128+K00128+K00253
Flavonoid biosynthesis	ko00941	10	64	715	13897	c102035.graph_c0;c102425.graph_c0;c105653.graph_c0;c111410.graph_c0;c116105.graph_c0;c124882.graph_c0;c128275.graph_c0;c129786.graph_c1;c93168.graph_c0;c94554.graph_c0	K05278+K00660+K01859+K13065+K13065+K05277+K05280+K00475+K00660+K05278
Glycerolipid metabolism	ko00561	10	191	715	13897	c122856.graph_c0;c122868.graph_c0;c125264.graph_c0;c125626.graph_c0;c127052.graph_c0;c128735.graph_c0;c130294.graph_c0;c130417.graph_c0;c130417.graph_c1;c70983.graph_c0	K00011+K00128+K11155+K07407+K13508+K00128+K13508+K00128+K00128+K13508
Phagosome	ko04145	10	348	715	13897	c100589.graph_c0;c105010.graph_c0;c106546.graph_c0;c113632.graph_c0;c119051.graph_c0;c121921.graph_c0;c132440.graph_c0;c70889.graph_c0;c89047.graph_c0;c97672.graph_c0	K04392+K02152+K07342+K07375+K07897+K02150+K08492+K07374+K07374+K07375
Carbon fixation in photosynthetic organisms	ko00710	10	282	715	13897	c100613.graph_c0;c106243.graph_c0;c110055.graph_c0;c112914.graph_c0;c114199.graph_c0;c118802.graph_c0;c119204.graph_c0;c128281.graph_c0;c128366.graph_c0;c129490.graph_c0	K00025+K00134+K01602+K00134+K01100+K00134+K05298+K01610+K01595+K00026

Nucleotide excision repair	ko03420	9	225	715	13897	c116129.graph_c0;c118790.graph_c0;c128517.graph_c0;c129051.graph_c0;c133355.graph_c0;c134063.graph_c0;c51615.graph_c0;c92680.graph_c0;c97655.graph_c0	K10755+K10740+K07466+K02325+K10747+K10848+K03868+K10740+K03868
Fatty acid biosynthesis	ko00061	9	136	715	13897	c100226.graph_c0;c125610.graph_c0;c128312.graph_c0;c128974.graph_c0;c132202.graph_c0;c132570.graph_c0;c134376.graph_c0;c71372.graph_c0;c90012.graph_c0	K09458+K10781+K09458+K01897+K02160+K00059+K01963+K01897+K01962
Ascorbate and aldarate metabolism	ko00053	8	134	715	13897	c113248.graph_c0;c117257.graph_c0;c119013.graph_c0;c122868.graph_c0;c126509.graph_c1;c128735.graph_c0;c130417.graph_c0;c130417.graph_c1	K00012+K10046+K00469+K00128+K00012+K00128+K00128+K00128
Ribosome biogenesis in eukaryotes	ko03008	8	379	715	13897	c101856.graph_c0;c102431.graph_c0;c102485.graph_c0;c116289.graph_c0;c116900.graph_c0;c130536.graph_c0;c131538.graph_c1;c132640.graph_c1	K13288+K03115+K14568+K14521+K12619+K11131+K12619+K14567
Circadian rhythm - plant	ko04712	8	88	715	13897	c102425.graph_c0;c102431.graph_c0;c112186.graph_c0;c125387.graph_c0;c126452.graph_c1;c130328.graph_c0;c130482.graph_c0;c93168.graph_c0	K00660+K03115+K16166+K12127+K12130+K12129+K12118+K00660
Homologous recombination	ko03440	8	157	715	13897	c118790.graph_c0;c123433.graph_c0;c124099.graph_c0;c128517.graph_c0;c129332.graph_c0;c133309.graph_c0;c133809.graph_c0;c92680.graph_c0	K10740+K10875+K03553+K07466+K10879+K08775+K08775+K10740
Sulfur metabolism	ko00920	7	92	715	13897	c122665.graph_c0;c124334.graph_c0;c126398.graph_c0;c130262.graph_c0;c132024.graph_c0	K05907+K01738+K05907+K13034+K01738+K01738+K13034

						;c134598.graph_c0;c88948.graph_c0	
Nitrogen metabolism	ko00910	7	100	715	13897	c107127.graph_c0;c112691.graph_c0;c119465.graph_c0;c122414.graph_c0;c124635.graph_c0;c124742.graph_c0;c130648.graph_c0	K01674+K01725+K00261+K02575+K01915+K00366+K02575
Biosynthesis of unsaturated fatty acids	ko01040	7	111	715	13897	c111947.graph_c0;c115084.graph_c0;c117490.graph_c0;c120878.graph_c0;c121978.graph_c0;c127942.graph_c0;c132570.graph_c0	K10256+K10251+K10255+K10703+K10257+K10703+K00059
Fatty acid elongation	ko00062	7	86	715	13897	c108824.graph_c0;c115084.graph_c0;c120878.graph_c0;c123016.graph_c0;c126407.graph_c0;c127942.graph_c0;c70542.graph_c0	K01074+K10251+K10703+K15397+K15397+K10703+K15397
Tryptophan metabolism	ko00380	7	126	715	13897	c118570.graph_c0;c122868.graph_c0;c124752.graph_c0;c127788.graph_c0;c128735.graph_c0;c130417.graph_c0;c130417.graph_c1	K11816+K00128+K00164+K11816+K00128+K00128+K00128
Ubiquinone and other terpenoid-quinone biosynthesis	ko00130	7	74	715	13897	c110889.graph_c0;c118844.graph_c0;c119082.graph_c0;c119172.graph_c0;c123577.graph_c0;c125412.graph_c0;c132013.graph_c0	K03183+K14760+K01904+K00457+K02552+K01904+K00815
Glyoxylate and dicarboxylate metabolism	ko00630	7	252	715	13897	c100613.graph_c0;c102566.graph_c0;c110055.graph_c0;c110315.graph_c0;c117685.graph_c0;c124635.graph_c0;c129490.graph_c0	K00025+K00122+K01602+K00830+K15919+K01915+K00026
beta-Alanine metabolism	ko00410	7	176	715	13897	c118423.graph_c0;c118938.graph_c0;c122868.graph_c0;c128735.graph_c0;c129360.graph_c0;c130417.graph_c0;c130417.graph_c1	K00276+K05605+K00128+K00128+K01918+K00128+K00128
Pentose phosphate pathway	ko00030	6	196	715	13897	c119582.graph_c1;c120124.graph_c0;c120317.graph_c0;c122721.graph_c0;c50718.graph_c0;c51688.graph_c0	K01057+K01057+K01057+K01835+K01057+K01057

Lysine degradation	ko00310	6	136	715	13897	c109846.graph_c0;c122868.graph_c0;c124752.graph_c0;c128735.graph_c0;c130417.graph_c0;c130417.graph_c1	K14157+K00128+K00164+K00128+K00128+K00128
Mismatch repair	ko03430	6	132	715	13897	c116129.graph_c0;c118790.graph_c0;c128517.graph_c0;c132924.graph_c0;c133355.graph_c0;c92680.graph_c0	K10755+K10740+K07466+K10858+K10747+K10740
Porphyrin and chlorophyll metabolism	ko00860	6	131	715	13897	c123143.graph_c1;c125410.graph_c0;c127890.graph_c0;c127890.graph_c1;c130025.graph_c0;c98055.graph_c0	K03428+K10960+K03403+K03403+K13071+K03428
Thiamine metabolism	ko00730	6	45	715	13897	c108150.graph_c0;c112666.graph_c0;c117167.graph_c0;c121244.graph_c0;c127214.graph_c1;c127921.graph_c0	K01662+K03146+K04487+K03146+K04487+K00878
Proteasome	ko03050	5	264	715	13897	c116158.graph_c0;c118618.graph_c0;c121189.graph_c0;c130122.graph_c0;c88678.graph_c0	K02728+K02727+K03062+K03062+K02734
Linoleic acid metabolism	ko00591	5	39	715	13897	c102687.graph_c0;c129473.graph_c0;c134411.graph_c0;c134639.graph_c0;c23669.graph_c0	K00454+K00454+K00454+K00454+K00454
Glycine, serine and threonine metabolism	ko00260	5	226	715	13897	c110315.graph_c0;c116989.graph_c0;c117685.graph_c0;c118423.graph_c0;c118522.graph_c0	K00830+K00827+K15919+K00276+K00382
Alanine, aspartate and glutamate metabolism	ko00250	5	240	715	13897	c110315.graph_c0;c116989.graph_c0;c119465.graph_c0;c124635.graph_c0;c126751.graph_c0	K00830+K00827+K00261+K01915+K00294
Carotenoid biosynthesis	ko00906	5	64	715	13897	c116858.graph_c0;c121850.graph_c0;c122440.graph_c0;c91224.graph_c0;c95941.graph_c0	K02291+K02293+K09840+K09843+K09843
Brassinosteroid	ko00905	5	20	715	13897	c105790.graph_c0;c118316.graph_c0;c119899.	K12637+K09587+K12638+K15639+K

biosynthesis						graph_c0;c120722.graph_c0;c131529.graph_c0	15639
Isoquinoline alkaloid biosynthesis	ko00950	5	54	715	13897	c118423.graph_c0;c130866.graph_c0;c132013.graph_c0;c23725.graph_c0;c23725.graph_c1	K00276+K01592+K00815+K01592+K01592
Folate biosynthesis	ko00790	5	47	715	13897	c117198.graph_c0;c125242.graph_c0;c126629.graph_c0;c131644.graph_c0;c86038.graph_c0	K03635+K03639+K18482+K13950+K13941
Other glycan degradation	ko00511	5	81	715	13897	c104972.graph_c0;c123951.graph_c0;c129180.graph_c0;c133065.graph_c0;c94189.graph_c0	K15923+K01191+K01191+K01191+K01191
ABC transporters	ko02010	5	155	715	13897	c119157.graph_c0;c126614.graph_c0;c130859.graph_c0;c132418.graph_c0;c134352.graph_c0	K05658+K05658+K05658+K05658+K05658
RNA polymerase	ko03020	5	150	715	13897	c112573.graph_c0;c115938.graph_c0;c119983.graph_c0;c119994.graph_c0;c124206.graph_c0	K03013+K03008+K03019+K03026+K03014
Zeatin biosynthesis	ko00908	5	19	715	13897	c124810.graph_c0;c130317.graph_c0;c130652.graph_c1;c131011.graph_c0;c93206.graph_c0	K00279+K00279+K10717+K00279+K13495
Biotin metabolism	ko00780	5	39	715	13897	c100226.graph_c0;c111496.graph_c0;c124338.graph_c0;c128312.graph_c0;c132570.graph_c0	K09458+K01012+K00652+K09458+K00059
Steroid biosynthesis	ko00100	4	81	715	13897	c101929.graph_c0;c124637.graph_c0;c125112.graph_c0;c127277.graph_c0	K01824+K01853+K14423+K00511
Inositol phosphate metabolism	ko00562	4	196	715	13897	c106470.graph_c0;c119013.graph_c0;c120471.graph_c0;c127088.graph_c0	K05857+K00469+K00915+K00889
Phosphatidylinositol signaling system	ko04070	4	194	715	13897	c106470.graph_c0;c112144.graph_c0;c120471.graph_c0;c127088.graph_c0	K05857+K02183+K00915+K00889
2-Oxocarboxylic acid metabolism	ko01210	4	216	715	13897	c117589.graph_c0;c122143.graph_c0;c123819.graph_c0;c93628.graph_c0	K00620+K00052+K00826+K01704

Sulfur relay system	ko04122	4	37	715	13897	c117167.graph_c0;c117198.graph_c0;c125242.graph_c0;c127214.graph_c1	K04487+K03635+K03639+K04487
Sphingolipid metabolism	ko00600	4	111	715	13897	c116191.graph_c0;c125626.graph_c0;c132695.graph_c0;c133691.graph_c1	K00654+K07407+K00654+K04713
Ether lipid metabolism	ko00565	4	87	715	13897	c111700.graph_c0;c128997.graph_c0;c130583.graph_c1;c133450.graph_c0	K01115+K01115+K01115+K01115
Histidine metabolism	ko00340	4	81	715	13897	c122868.graph_c0;c128735.graph_c0;c130417.graph_c0;c130417.graph_c1	K00128+K00128+K00128+K00128
Riboflavin metabolism	ko00740	4	26	715	13897	c112285.graph_c0;c112874.graph_c0;c125704.graph_c0;c129304.graph_c0	K14652+K14652+K14379+K14652
Tropane, piperidine and pyridine alkaloid biosynthesis	ko00960	4	67	715	13897	c118423.graph_c0;c122496.graph_c0;c125213.graph_c1;c132013.graph_c0	K00276+K08081+K08081+K00815
Propanoate metabolism	ko00640	4	140	715	13897	c118938.graph_c0;c132202.graph_c0;c134376.graph_c0;c90012.graph_c0	K05605+K02160+K01963+K01962
SNARE interactions in vesicular transport	ko04130	4	99	715	13897	c113095.graph_c0;c116715.graph_c0;c122899.graph_c0;c132440.graph_c0	K08506+K08486+K08506+K08492
Base excision repair	ko03410	4	137	715	13897	c110971.graph_c0;c129051.graph_c0;c133355.graph_c0;c97798.graph_c0	K10798+K02325+K10747+K10798
Regulation of autophagy	ko04140	3	134	715	13897	c120184.graph_c0;c125459.graph_c0;c128260.graph_c0	K07198+K07198+K07198
Arachidonic	ko00590	3	51	715	13897	c108187.graph_c0;c118215.graph_c0;c127844.	K00432+K00079+K18592

acid metabolism						graph_c0	
Monoterpenoid biosynthesis	ko00902	3	6	715	13897	c102174.graph_c0;c119420.graph_c0;c122797.graph_c0	K15086+K15095+K15086
Aminoacyl-tRNA biosynthesis	ko00970	3	289	715	13897	c129854.graph_c0;c131414.graph_c0;c78250.graph_c0	K14164+K01866+K01893
Sesquiterpenoid and triterpenoid biosynthesis	ko00909	3	22	715	13897	c122391.graph_c0;c124467.graph_c0;c127277.graph_c0	K15813+K15803+K00511
Basal transcription factors	ko03022	3	135	715	13897	c114404.graph_c0;c124056.graph_c0;c133777.graph_c0	K03139+K03120+K03131
Lysine biosynthesis	ko00300	3	42	715	13897	c114056.graph_c0;c123498.graph_c0;c70627.graph_c0	K10206+K00215+K01778
Limonene and pinene degradation	ko00903	3	28	715	13897	c122868.graph_c0;c130417.graph_c0;c130417.graph_c1	K00128+K00128+K00128
Pantothenate and CoA biosynthesis	ko00770	3	80	715	13897	c114123.graph_c0;c123819.graph_c0;c129360.graph_c0	K02201+K00826+K01918
Valine, leucine and isoleucine biosynthesis	ko00290	3	58	715	13897	c122143.graph_c0;c123819.graph_c0;c93628.graph_c0	K00052+K00826+K01704
C5-Branched dibasic acid metabolism	ko00660	2	28	715	13897	c122143.graph_c0;c93628.graph_c0	K00052+K01704

Glycosylphosphatidylinositol(GPI)-anchor biosynthesis	ko00563	2	58	715	13897	c105535.graph_c0;c118365.graph_c0	K11001+K07541
Flavone and flavonol biosynthesis	ko00944	2	6	715	13897	c128275.graph_c0;c92897.graph_c0	K05280+K13272
Protein export	ko03060	2	165	715	13897	c106546.graph_c0;c129331.graph_c0	K07342+K12948
Degradation of aromatic compounds	ko01220	2	27	715	13897	c108564.graph_c0;c126578.graph_c0	K00001+K00121
Synthesis and degradation of ketone bodies	ko00072	2	27	715	13897	c112284.graph_c0;c122610.graph_c0	K01641+K01640
Butanoate metabolism	ko00650	2	85	715	13897	c112284.graph_c0;c122610.graph_c0	K01641+K01640
Diterpenoid biosynthesis	ko00904	2	25	715	13897	c113336.graph_c0;c130968.graph_c0	K04125+K04121
Stilbenoid, diarylheptanoid and gingerol biosynthesis	ko00945	2	34	715	13897	c111410.graph_c0;c116105.graph_c0	K13065+K13065
Taurine and hypotaurine metabolism	ko00430	2	26	715	13897	c121597.graph_c0;c127844.graph_c0	K10712+K18592

Vitamin B6 metabolism	ko00750	1	38	715	13897	c90381.graph_c0	K13248
Glycosphingolipid biosynthesis - globo series	ko00603	1	21	715	13897	c125626.graph_c0	K07407
Other types of O-glycan biosynthesis	ko00514	1	18	715	13897	c88959.graph_c0	K09667
Selenocompound metabolism	ko00450	1	89	715	13897	c120855.graph_c0	K01761
N-Glycan biosynthesis	ko00510	1	156	715	13897	c117730.graph_c0	K00737
Nicotinate and nicotinamide metabolism	ko00760	1	43	715	13897	c121893.graph_c0	K01081
Anthocyanin biosynthesis	ko00942	1	4	715	13897	c117163.graph_c0	K12338
Non-homologous end-joining	ko03450	1	24	715	13897	c109375.graph_c0	K10884
Vancomycin resistance	ko01502	1	2	715	13897	c108176.graph_c0	K02563

Table S3. Putative unigenes related to flower pigmentation.

Pathway	Gene	Enzyme	Unigene number
phenylpropanoid biosynthesis	*PAL*	Phenylalanine ammonia-lyase	44
Flavonoid biosynthesis	*CHS*	Chalcone synthase	6
	CHI	Chalcone isomerase	5
	F3H	Flavanone 3-hydroxylase	2
	F3'H	Flavanone 3'-hydroxylase	5
	FLS	Flavonol synthase	2
	DFR	Dihydroflavonol-4-reductase	3
	ANS	Anthocyanidin synthase	4
Anthocyanin biosynthesis	*AOMT*	Anthocyanin O-methyltransferase	4
	3GT	Anthocyanin 3-O-glycosyltransferase	3
	5GT	Anthocyanin 5-O-glycosyltransferase	1

Table S4. Putative unigenes related to transcriptional regulation and transferase activity.

Function	Gene	Enzyme	Unigene number
Transcriptional regulation	*MYB*	MYB transcription factor	88
	bHLH	bHLH transcription factor	16
	WD	WD-repeat protein	44
Transferase activity	*GST*	Glutathione S-transferase	64

Table S5. KEGG pathway classification of DEGs in PO vs PR comparison.

#Pathway	ko_ID	Unigene	gene	Unigene_all	gene_all		
#KEGG_n	477	KEGG_N	13897				
Starch and sucrose metabolism	ko00500	36	469	477	13897	c105513.graph_c1;c106004.graph_c0;c108042.graph_c0;c108790.graph_c0;c109671.graph_c0;c115711.graph_c0;c117587.graph_c0;c117822.graph_c0;c118113.graph_c0;c118816.graph_c0;c119141.graph_c0;c119325.graph_c0;c120576.graph_c0;c121742.graph_c0;c122721.graph_c0;c123210.graph_c0;c123220.graph_c0;c125155.graph_c1;c126580.graph_c0;c126811.graph_c0;c126934.graph_c0;c127107.graph_c0;c127792.graph_c0;c129616.graph_c0;c129750.graph_c0;c130158.graph_c0;c130187.graph_c0;c130727.graph_c1;c131002.graph_c1;c131646.graph_c1;c131799.graph_c0;c23686.graph_c0;c71123.graph_c0;c86535.graph_c0;c88845.graph_c0;c97687.graph_c0	K01188+K01188+K18447+K01188+K05350+K01176+K00695+K01051+K01188+K01188+K00696+K01184+K01051+K01051+K01835+K05350+K01188+K01176+K01177+K01193+K01187+K00847+K01188+K01051+K01051+K01087+K00847+K01176+K00696+K00688+K01176+K01177+K01051+K01188+K05350+K01188
Plant hormone signal transduction	ko04075	33	403	477	13897	c100991.graph_c0;c105815.graph_c0;c106087.graph_c0;c107593.graph_c0;c108427.graph_c0;c109272.graph_c0;c109646.graph_c0;c112138.graph_c0;c112179.graph_c0;c113017.graph_c0;c116573.graph_c0;c116665.graph_c0;c116968.grap	K14487+K14505+K14490+K14492+K14490+K14492+K14488+K13464+K14488+K14484+K14505+K14488+K14432+K14487+K14488+K14496+K14488+K14488+K14497+K14498+K13464+K14484+K14505+K14490+K14512+K14491+K14491+K14488+K14488+K14505+

						h_c0;c117784.graph_c0;c120105.graph_c1;c120345.graph_c0;c120847.graph_c0;c121149.graph_c0;c121691.graph_c0;c121767.graph_c0;c122051.graph_c0;c122394.graph_c0;c123048.graph_c0;c124750.graph_c1;c124826.graph_c0;c125541.graph_c0;c127036.graph_c1;c127324.graph_c0;c127580.graph_c0;c127695.graph_c0;c130168.graph_c0;c132781.graph_c0;c133064.graph_c0	K14509+K14492+K14491
Phenylpropanoid biosynthesis	ko00940	29	250	477	13897	c101742.graph_c0;c105513.graph_c1;c106004.graph_c0;c107475.graph_c0;c108790.graph_c0;c109671.graph_c0;c111428.graph_c0;c111764.graph_c0;c114770.graph_c0;c116628.graph_c0;c117640.graph_c0;c118113.graph_c0;c118247.graph_c0;c118816.graph_c0;c120064.graph_c0;c120064.graph_c1;c123210.graph_c0;c123220.graph_c0;c125412.graph_c0;c127792.graph_c0;c129902.graph_c0;c130735.graph_c0;c133930.graph_c0;c50245.graph_c0;c86535.graph_c0;c86972.graph_c0;c88845.graph_c0;c89173.graph_c0;c97687.graph_c0	K00083+K01188+K01188+K00430+K01188+K05350+K00430+K11188+K13066+K00430+K12356+K01188+K00430+K01188+K13066+K13066+K05350+K01188+K01904+K01188+K13066+K00430+K00430+K00083+K01188+K11188+K05350+K09755+K01188
Ribosome	ko03010	24	996	477	13897	c102478.graph_c0;c102533.graph_c0;c105246.graph_c0;c108452.graph_c1;c111	K02939+K02937+K02940+K02922+K02866+K02922+K02988+K02880+K02959+K02896+

						222.graph_c0;c114175.graph_c0;c118085.graph_c0;c119933.graph_c0;c122506.graph_c0;c124830.graph_c0;c125171.graph_c0;c125852.graph_c0;c125958.graph_c0;c129136.graph_c0;c130486.graph_c0;c132355.graph_c0;c132848.graph_c0;c133030.graph_c0;c133694.graph_c0;c133984.graph_c0;c134599.graph_c0;c49761.graph_c0;c90571.graph_c0;c95596.graph_c0	K02937+K02896+K02993+K02938+K02882+K02980+K02992+K02937+K02882+K02868+K02960+K02938+K02949+K02894
Biosynthesis of amino acids	ko01230	23	812	477	13897	c101979.graph_c0;c102878.graph_c0;c114056.graph_c0;c117589.graph_c0;c117781.graph_c1;c118697.graph_c0;c121090.graph_c0;c121536.graph_c0;c122230.graph_c0;c123819.graph_c0;c124334.graph_c0;c124635.graph_c0;c124834.graph_c0;c129927.graph_c0;c132024.graph_c0;c133226.graph_c0;c49644.graph_c0;c51012.graph_c0;c51284.graph_c0;c71676.graph_c0;c88948.graph_c0;c93628.graph_c0;c95528.graph_c0	K05359+K15227+K10206+K00620+K06001+K00611+K15227+K00815+K01695+K00826+K01738+K01915+K00615+K13832+K01738+K13832+K00030+K00789+K00600+K15227+K13034+K01704+K00891
Carbon metabolism	ko01200	22	955	477	13897	c100613.graph_c0;c102566.graph_c0;c110315.graph_c0;c118938.graph_c0;c120124.graph_c0;c123772.graph_c0;c124334.graph_c0;c124834.graph_c0;c126578.graph_c0;c128281.graph_c0;c128366.gr	K00025+K00122+K00830+K05605+K01057+K00855+K01738+K00615+K00121+K01610+K01595+K00026+K01738+K01963+K01610+K00030+K00600+K01895+K00627+K01679+K13034+K01962

						aph_c0;c129490.graph_c0;c132024.graph_c0;c134376.graph_c0;c134605.graph_c0;c49644.graph_c0;c51284.graph_c0;c62501.graph_c0;c85077.graph_c0;c88872.graph_c0;c88948.graph_c0;c90012.graph_c0	
Protein processing in endoplasmic reticulum	ko04141	22	868	477	13897	c102059.graph_c0;c108531.graph_c0;c109699.graph_c0;c115661.graph_c0;c117552.graph_c0;c118015.graph_c0;c118078.graph_c0;c120059.graph_c0;c121542.graph_c1;c122224.graph_c0;c122836.graph_c0;c123347.graph_c0;c124083.graph_c0;c125019.graph_c0;c125391.graph_c1;c129495.graph_c0;c131854.graph_c1;c23697.graph_c0;c71721.graph_c0;c90083.graph_c0;c97655.graph_c0;c97880.graph_c0	K13993+K03094+K13993+K13993+K03347+K13993+K13993+K03237+K13993+K13993+K13993+K03094+K09503+K13993+K13993+K08852+K03283+K03283+K13993+K13993+K03868+K06689
Purine metabolism	ko00230	19	563	477	13897	c108042.graph_c0;c110950.graph_c0;c111706.graph_c0;c112310.graph_c0;c112378.graph_c0;c117973.graph_c0;c120834.graph_c0;c122370.graph_c0;c122721.graph_c0;c124206.graph_c0;c125604.graph_c0;c128678.graph_c0;c129051.graph_c0;c130274.graph_c0;c130689.graph_c1;c131570.graph_c0;c131878.graph_c0;c64367.graph_c0;c99075.graph_c0	K18447+K10808+K00759+K11808+K00856+K06928+K01514+K00759+K01835+K03014+K03040+K10807+K02325+K01427+K00939+K01923+K10808+K02326+K00088

Plant-pathogen interaction	ko04626	19	373	477	13897	c112144.graph_c0;c112507.graph_c0;c115120.graph_c0;c119307.graph_c0;c119888.graph_c0;c121838.graph_c0;c122045.graph_c0;c125898.graph_c1;c129591.graph_c0;c130786.graph_c0;c131652.graph_c1;c132441.graph_c0;c132982.graph_c1;c133136.graph_c0;c134348.graph_c0;c134380.graph_c0;c50350.graph_c0;c88346.graph_c0;c91463.graph_c0	K02183+K13448+K13448+K13412+K13412+K13459+K18875+K13457+K05391+K13429+K13420+K13412+K13447+K13447+K13457+K13459+K13448+K13412+K12795
alpha-Linolenic acid metabolism	ko00592	18	130	477	13897	c102004.graph_c0;c102687.graph_c0;c109303.graph_c0;c109581.graph_c0;c115772.graph_c0;c117008.graph_c0;c118104.graph_c0;c118459.graph_c0;c120300.graph_c0;c121567.graph_c0;c123502.graph_c0;c123665.graph_c0;c125727.graph_c0;c133558.graph_c0;c134411.graph_c0;c134639.graph_c0;c23669.graph_c0;c98181.graph_c0	K18857+K00454+K08241+K18857+K01723+K10525+K18857+K01723+K05894+K01723+K00232+K10525+K08241+K00232+K00454+K00454+K00454+K01047
Photosynthesis	ko00195	17	144	477	13897	c100128.graph_c0;c101644.graph_c0;c104922.graph_c0;c105700.graph_c0;c108958.graph_c0;c109514.graph_c0;c109836.graph_c0;c111404.graph_c0;c120141.graph_c0;c124921.graph_c0;c125492.graph_c0;c126976.graph_c0;c128494.graph_c0;c130722.graph_c0;c132793.graph_c0;c95982.graph_c0;c97392.graph_c0	K02115+K02638+K02639+K08902+K02641+K02692+K02115+K14332+K02707+K02694+K02109+K02113+K02691+K02710+K02705+K02641+K02717

Oxidative phosphorylation	ko00190	15	552	477	13897	c100128.graph_c0;c105010.graph_c0;c109836.graph_c0;c112664.graph_c0;c120026.graph_c0;c121921.graph_c0;c125492.graph_c0;c126524.graph_c0;c126976.graph_c0;c129271.graph_c0;c132160.graph_c0;c132549.graph_c0;c133939.graph_c0;c28152.graph_c0;c71577.graph_c0	K02115+K02152+K02115+K01535+K05577+K02150+K02109+K05582+K02113+K03883+K03881+K02132+K05579+K01535+K01535
Cyanoamino acid metabolism	ko00460	15	114	477	13897	c105513.graph_c1;c106004.graph_c0;c108790.graph_c0;c109671.graph_c0;c118113.graph_c0;c118816.graph_c0;c123210.graph_c0;c123220.graph_c0;c127792.graph_c0;c127844.graph_c0;c51284.graph_c0;c86535.graph_c0;c88845.graph_c0;c88948.graph_c0;c97687.graph_c0	K01188+K01188+K01188+K05350+K01188+K01188+K05350+K01188+K01188+K18592+K00600+K01188+K05350+K13034+K01188
Glutathione metabolism	ko00480	14	235	477	13897	c110950.graph_c0;c111372.graph_c0;c112965.graph_c0;c117302.graph_c0;c122681.graph_c0;c125111.graph_c0;c125826.graph_c0;c127844.graph_c0;c128678.graph_c0;c131878.graph_c0;c51066.graph_c0;c71042.graph_c0;c96247.graph_c0;c98149.graph_c0	K10808+K00799+K00799+K00797+K00799+K00799+K00799+K18592+K10807+K10808+K00799+K00799+K00799+K00799
Phenylalanine metabolism	ko00360	14	171	477	13897	c107475.graph_c0;c110715.graph_c0;c111428.graph_c0;c111764.graph_c0;c116628.graph_c0;c118247.graph_c0;c118423.graph_c0;c119172.graph_c0;c121536.graph_c0;c125412.graph_c0;c130735.gr	K00430+K07253+K00430+K11188+K00430+K00430+K00276+K00457+K00815+K01904+K00430+K00276+K00430+K11188

						aph_c0;c131042.graph_c0;c133930.graph_c0;c86972.graph_c0	
Ubiquitin mediated proteolysis	ko04120	14	468	477	13897	c100543.graph_c0;c108531.graph_c0;c108920.graph_c0;c115011.graph_c0;c117552.graph_c0;c119129.graph_c0;c123174.graph_c0;c123347.graph_c0;c123525.graph_c0;c128987.graph_c0;c131884.graph_c0;c133559.graph_c0;c97655.graph_c0;c97880.graph_c0	K10579+K03094+K10144+K10144+K03347+K10583+K03354+K03094+K03363+K10581+K12456+K10144+K03868+K06689
Cysteine and methionine metabolism	ko00270	13	289	477	13897	c100613.graph_c0;c117302.graph_c0;c120405.graph_c1;c120855.graph_c0;c121536.graph_c0;c123728.graph_c0;c124334.graph_c0;c125674.graph_c0;c125789.graph_c0;c129490.graph_c0;c132024.graph_c0;c51012.graph_c0;c88948.graph_c0	K00025+K00797+K00016+K01761+K00815+K01762+K01738+K00547+K05933+K00026+K01738+K00789+K13034
Glycolysis / Gluconeogenesis	ko00010	13	448	477	13897	c102004.graph_c0;c109581.graph_c0;c111937.graph_c0;c118104.graph_c0;c120405.graph_c1;c122721.graph_c0;c126578.graph_c0;c128281.graph_c0;c128735.graph_c0;c134543.graph_c0;c134605.graph_c0;c62501.graph_c0;c85077.graph_c0	K18857+K18857+K01785+K18857+K00016+K01835+K00121+K01610+K00128+K01568+K01610+K01895+K00627
Pyruvate metabolism	ko00620	12	357	477	13897	c100613.graph_c0;c120405.graph_c1;c128281.graph_c0;c128366.graph_c0;c128735.graph_c0;c129490.graph_c0;c13437	K00025+K00016+K01610+K01595+K00128+K00026+K01963+K01610+K01895+K00627+K01679+K01962

						6.graph_c0;c134605.graph_c0;c62501.graph_c0;c85077.graph_c0;c88872.graph_c0;c90012.graph_c0	
Tyrosine metabolism	ko00350	12	121	477	13897	c102004.graph_c0;c109581.graph_c0;c110715.graph_c0;c118104.graph_c0;c118423.graph_c0;c119172.graph_c0;c121536.graph_c0;c126578.graph_c0;c130866.graph_c0;c131042.graph_c0;c23725.graph_c0;c23725.graph_c1	K18857+K18857+K07253+K18857+K00276+K00457+K00815+K00121+K01592+K00276+K01592+K01592
Endocytosis	ko04144	11	472	477	13897	c108935.graph_c0;c111700.graph_c0;c112162.graph_c0;c114229.graph_c0;c118376.graph_c0;c127088.graph_c0;c131641.graph_c0;c131641.graph_c1;c131854.graph_c1;c133450.graph_c0;c23697.graph_c0	K07904+K01115+K07904+K12489+K01115+K00889+K12489+K12489+K03283+K01115+K03283
Cutin, suberine and wax biosynthesis	ko00073	11	60	477	13897	c124424.graph_c0;c124583.graph_c0;c127594.graph_c0;c128040.graph_c0;c131711.graph_c0;c132698.graph_c0;c133529.graph_c0;c86343.graph_c0;c88162.graph_c0;c97209.graph_c0;c99637.graph_c1	K15404+K15398+K17991+K13356+K15404+K15398+K13407+K15398+K13356+K13356+K13356
Pentose and glucuronate interconversions	ko00040	11	202	477	13897	c117822.graph_c0;c119325.graph_c0;c120576.graph_c0;c121742.graph_c0;c122856.graph_c0;c124896.graph_c0;c128735.graph_c0;c129616.graph_c0;c129750.graph_c0;c23628.graph_c0;c71123.grap	K01051+K01184+K01051+K01051+K00011+K01728+K00128+K01051+K01051+K01728+K01051

						h_c0	
RNA degradation	ko03018	10	419	477	13897	c109491.graph_c0;c116941.graph_c0;c120290.graph_c0;c125901.graph_c0;c126315.graph_c0;c130826.graph_c0;c131538.graph_c1;c134322.graph_c0;c134441.graph_c0;c93753.graph_c0	K04077+K12606+K12608+K04077+K13126+K12603+K12619+K14442+K12619+K14442
Terpenoid backbone biosynthesis	ko00900	10	155	477	13897	c111454.graph_c0;c113927.graph_c0;c121595.graph_c0;c121595.graph_c1;c122587.graph_c0;c124462.graph_c0;c125410.graph_c0;c127762.graph_c1;c81933.graph_c0;c88832.graph_c0	K13789+K05356+K01662+K01662+K15889+K13789+K10960+K11778+K00021+K00021
Phenylalanine, tyrosine and tryptophan biosynthesis	ko00400	10	117	477	13897	c101979.graph_c0;c102878.graph_c0;c117781.graph_c1;c121090.graph_c0;c121536.graph_c0;c122230.graph_c0;c129927.graph_c0;c133226.graph_c0;c71676.graph_c0;c95528.graph_c0	K05359+K15227+K06001+K15227+K00815+K01695+K13832+K13832+K15227+K00891
Amino sugar and nucleotide sugar metabolism	ko00520	10	335	477	13897	c106554.graph_c0;c122721.graph_c0;c123063.graph_c0;c124607.graph_c0;c127107.graph_c0;c130187.graph_c0;c131811.graph_c0;c54775.graph_c0;c88590.graph_c0;c89832.graph_c0	K01183+K01835+K01183+K01209+K00847+K00847+K01183+K01784+K01784+K12449
Fatty acid metabolism	ko01212	10	268	477	13897	c100226.graph_c0;c108824.graph_c0;c115084.graph_c0;c121978.graph_c0;c123502.graph_c0;c124335.graph_c0;c132570.graph_c0;c133558.graph_c0;c134376.graph_c0;c90012.graph_c0	K09458+K01074+K10251+K10257+K00232+K10781+K00059+K00232+K01963+K01962

Pyrimidine metabolism	ko00240	10	456	477	13897	c102867.graph_c0;c110950.graph_c0;c114780.graph_c0;c124206.graph_c0;c125604.graph_c0;c128678.graph_c0;c129051.graph_c0;c131878.graph_c0;c64367.graph_c0;c93528.graph_c0	K00857+K10808+K00876+K03014+K03040+K10807+K02325+K10808+K02326+K01520
Glycerophospholipid metabolism	ko00564	9	243	477	13897	c105872.graph_c0;c111700.graph_c0;c118376.graph_c0;c124631.graph_c0;c127052.graph_c0;c128220.graph_c0;c130294.graph_c0;c133450.graph_c0;c98181.graph_c0	K06130+K01115+K01115+K06129+K13508+K13513+K13508+K01115+K01047
Galactose metabolism	ko00052	9	166	477	13897	c111937.graph_c0;c121255.graph_c1;c122721.graph_c0;c122856.graph_c0;c126811.graph_c0;c126934.graph_c0;c127648.graph_c0;c54775.graph_c0;c88590.graph_c0	K01785+K06617+K01835+K00011+K01193+K01187+K18819+K01784+K01784
Peroxisome	ko04146	9	287	477	13897	c110315.graph_c0;c123153.graph_c0;c123502.graph_c0;c128040.graph_c0;c129908.graph_c0;c133558.graph_c0;c88162.graph_c0;c97209.graph_c0;c99637.graph_c1	K00830+K13346+K00232+K13356+K11517+K00232+K13356+K13356+K13356
RNA transport	ko03013	8	630	477	13897	c102019.graph_c0;c116762.graph_c0;c117960.graph_c0;c120059.graph_c0;c125991.graph_c0;c126315.graph_c0;c130438.graph_c0;c131752.graph_c0	K12882+K03257+K03260+K03237+K03231+K13126+K14293+K03250
Thiamine metabolism	ko00730	8	45	477	13897	c112666.graph_c0;c117167.graph_c0;c117973.graph_c0;c121244.graph_c0;c121	K03146+K04487+K06928+K03146+K01662+K01662+K04487+K00878

						595.graph_c0;c121595.graph_c1;c127214.graph_c1;c127921.graph_c0	
Glyoxylate and dicarboxylate metabolism	ko00630	8	252	477	13897	c100613.graph_c0;c102566.graph_c0;c110315.graph_c0;c124635.graph_c0;c129490.graph_c0;c129908.graph_c0;c51284.graph_c0;c96010.graph_c0	K00025+K00122+K00830+K01915+K00026+K11517+K00600+K02437
Phagosome	ko04145	7	348	477	13897	c105010.graph_c0;c113632.graph_c0;c121921.graph_c0;c123483.graph_c0;c132440.graph_c0;c70889.graph_c0;c97672.graph_c0	K02152+K07375+K02150+K04392+K08492+K07374+K07375
mRNA surveillance pathway	ko03015	7	452	477	13897	c102019.graph_c0;c118482.graph_c0;c124542.graph_c0;c126315.graph_c0;c129634.graph_c0;c132292.graph_c2;c88662.graph_c0	K12882+K03456+K14400+K13126+K03265+K03456+K03265
Fructose and mannose metabolism	ko00051	7	201	477	13897	c108042.graph_c0;c114913.graph_c0;c118742.graph_c0;c122856.graph_c0;c127107.graph_c0;c130187.graph_c0;c130978.graph_c0	K18447+K00895+K00895+K00011+K00847+K00847+K00895
Zeatin biosynthesis	ko00908	7	19	477	13897	c124810.graph_c0;c125250.graph_c0;c129356.graph_c0;c130317.graph_c0;c130652.graph_c1;c131511.graph_c0;c132025.graph_c0	K00279+K10760+K13495+K00279+K10717+K00791+K00279
Fatty acid degradation	ko00071	7	197	477	13897	c102004.graph_c0;c109581.graph_c0;c118104.graph_c0;c123502.graph_c0;c126578.graph_c0;c128735.graph_c0;c133558.graph_c0	K18857+K18857+K18857+K00232+K00121+K00128+K00232

Glycine, serine and threonine metabolism	ko00260	7	226	477	13897	c110315.graph_c0;c117781.graph_c1;c118423.graph_c0;c122230.graph_c0;c131042.graph_c0;c51284.graph_c0;c96010.graph_c0	K00830+K06001+K00276+K01695+K00276+K00600+K02437
Citrate cycle (TCA cycle)	ko00020	7	304	477	13897	c100613.graph_c0;c128281.graph_c0;c129490.graph_c0;c134605.graph_c0;c49644.graph_c0;c85077.graph_c0;c88872.graph_c0	K00025+K01610+K00026+K01610+K00030+K00627+K01679
Carbon fixation in photosynthetic organisms	ko00710	7	282	477	13897	c100613.graph_c0;c123772.graph_c0;c124834.graph_c0;c128281.graph_c0;c128366.graph_c0;c129490.graph_c0;c134605.graph_c0	K00025+K00855+K00615+K01610+K01595+K00026+K01610
Isoquinoline alkaloid biosynthesis	ko00950	6	54	477	13897	c118423.graph_c0;c121536.graph_c0;c130866.graph_c0;c131042.graph_c0;c23725.graph_c0;c23725.graph_c1	K00276+K00815+K01592+K00276+K01592+K01592
Porphyrin and chlorophyll metabolism	ko00860	6	131	477	13897	c121882.graph_c0;c123884.graph_c0;c125410.graph_c0;c127890.graph_c0;c127890.graph_c1;c130025.graph_c0	K04035+K00218+K10960+K03403+K03403+K13071
ABC transporters	ko02010	6	155	477	13897	c119157.graph_c0;c126614.graph_c0;c130859.graph_c0;c131196.graph_c0;c132418.graph_c0;c134352.graph_c0	K05658+K05658+K05658+K05658+K05658+K05658
Base excision repair	ko03410	6	137	477	13897	c110971.graph_c0;c121800.graph_c0;c124043.graph_c0;c129051.graph_c0;c64367.graph_c0;c97798.graph_c0	K10798+K01142+K10798+K02325+K02326+K10798
Arginine and proline metabolism	ko00330	6	287	477	13897	c117302.graph_c0;c117589.graph_c0;c118697.graph_c0;c124635.graph_c0;c128	K00797+K00620+K00611+K01915+K00128+K01427

						735.graph_c0;c130274.graph_c0	
Photosynthesis - antenna proteins	ko00196	6	52	477	13897	c100527.graph_c0;c106848.graph_c0;c109381.graph_c0;c114287.graph_c0;c71681.graph_c0;c89965.graph_c0	K08910+K08909+K08916+K08908+K08913+K08913
Circadian rhythm - plant	ko04712	6	88	477	13897	c112186.graph_c0;c126452.graph_c1;c127832.graph_c0;c130328.graph_c0;c132912.graph_c0;c93168.graph_c0	K16166+K12130+K16241+K12129+K12129+K00660
Spliceosome	ko03040	6	868	477	13897	c102019.graph_c0;c120047.graph_c0;c131854.graph_c1;c23697.graph_c0;c71357.graph_c0;c91908.graph_c0	K12882+K12830+K03283+K03283+K12833+K12833
Brassinosteroid biosynthesis	ko00905	5	20	477	13897	c105790.graph_c0;c118316.graph_c0;c119899.graph_c0;c120722.graph_c0;c97361.graph_c0	K12637+K09587+K12638+K15639+K15639
Biosynthesis of unsaturated fatty acids	ko01040	5	111	477	13897	c115084.graph_c0;c121978.graph_c0;c123502.graph_c0;c132570.graph_c0;c133558.graph_c0	K10251+K10257+K00232+K00059+K00232
Linoleic acid metabolism	ko00591	5	39	477	13897	c102687.graph_c0;c134411.graph_c0;c134639.graph_c0;c23669.graph_c0;c98181.graph_c0	K00454+K00454+K00454+K00454+K01047
beta-Alanine metabolism	ko00410	5	176	477	13897	c117302.graph_c0;c118423.graph_c0;c118938.graph_c0;c128735.graph_c0;c131042.graph_c0	K00797+K00276+K05605+K00128+K00276
Propanoate metabolism	ko00640	5	140	477	13897	c118938.graph_c0;c120405.graph_c1;c134376.graph_c0;c62501.graph_c0;c90012.graph_c0	K05605+K00016+K01963+K01895+K01962
Glycerolipid	ko00561	5	191	477	13897	c122856.graph_c0;c127052.graph_c0;c1	K00011+K13508+K13513+K00128+K13508

metabolism						28220.graph_c0;c128735.graph_c0;c130294.graph_c0	
Nitrogen metabolism	ko00910	5	100	477	13897	c107127.graph_c0;c112691.graph_c0;c122099.graph_c0;c124635.graph_c0;c130648.graph_c0	K01674+K01725+K01673+K01915+K02575
Fatty acid biosynthesis	ko00061	5	136	477	13897	c100226.graph_c0;c124335.graph_c0;c132570.graph_c0;c134376.graph_c0;c90012.graph_c0	K09458+K10781+K00059+K01963+K01962
Fatty acid elongation	ko00062	4	86	477	13897	c101374.graph_c0;c108824.graph_c0;c115084.graph_c0;c70542.graph_c0	K15397+K01074+K10251+K15397
Ether lipid metabolism	ko00565	4	87	477	13897	c111700.graph_c0;c118376.graph_c0;c133450.graph_c0;c98181.graph_c0	K01115+K01115+K01115+K01047
Ubiquinone and other terpenoid-quinone biosynthesis	ko00130	4	74	477	13897	c119172.graph_c0;c121536.graph_c0;c123577.graph_c0;c125412.graph_c0	K00457+K00815+K02552+K01904
DNA replication	ko03030	4	216	477	13897	c118790.graph_c0;c121683.graph_c0;c129051.graph_c0;c64367.graph_c0	K10740+K10745+K02325+K02326
Nucleotide excision repair	ko03420	4	225	477	13897	c118790.graph_c0;c129051.graph_c0;c64367.graph_c0;c97655.graph_c0	K10740+K02325+K02326+K03868
Alanine, aspartate and glutamate metabolism	ko00250	4	240	477	13897	c110315.graph_c0;c120459.graph_c0;c124635.graph_c0;c130166.graph_c0	K00830+K00278+K01915+K01953
2-Oxocarboxylic acid metabolism	ko01210	4	216	477	13897	c117589.graph_c0;c123819.graph_c0;c49644.graph_c0;c93628.graph_c0	K00620+K00826+K00030+K01704
Ribosome	ko03008	4	379	477	13897	c116289.graph_c0;c122033.graph_c0;c1	K14521+K14538+K12619+K12619

biogenesis in eukaryotes						31538.graph_c1;c134441.graph_c0	
SNARE interactions in vesicular transport	ko04130	4	99	477	13897	c109420.graph_c0;c113095.graph_c0;c122232.graph_c1;c132440.graph_c0	K08486+K08506+K08486+K08492
Flavonoid biosynthesis	ko00941	4	64	477	13897	c105653.graph_c0;c126380.graph_c0;c93168.graph_c0;c94554.graph_c0	K01859+K13082+K00660+K05278
Tropane, piperidine and pyridine alkaloid biosynthesis	ko00960	4	67	477	13897	c118423.graph_c0;c121536.graph_c0;c122496.graph_c0;c131042.graph_c0	K00276+K00815+K08081+K00276
Pentose phosphate pathway	ko00030	3	196	477	13897	c120124.graph_c0;c122721.graph_c0;c124834.graph_c0	K01057+K01835+K00615
Valine, leucine and isoleucine degradation	ko00280	3	223	477	13897	c118938.graph_c0;c123819.graph_c0;c128735.graph_c0	K05605+K00826+K00128
Riboflavin metabolism	ko00740	3	26	477	13897	c112874.graph_c0;c125704.graph_c0;c129304.graph_c0	K14652+K14379+K14652
Sulfur relay system	ko04122	3	37	477	13897	c117167.graph_c0;c125242.graph_c0;c127214.graph_c1	K04487+K03639+K04487
Sulfur metabolism	ko00920	3	92	477	13897	c124334.graph_c0;c132024.graph_c0;c88948.graph_c0	K01738+K01738+K13034
Tryptophan metabolism	ko00380	3	126	477	13897	c125003.graph_c0;c127788.graph_c0;c128735.graph_c0	K07408+K11816+K00128
Diterpenoid biosynthesis	ko00904	3	25	477	13897	c113336.graph_c0;c130968.graph_c0;c89332.graph_c0	K04125+K04121+K17982

Folate biosynthesis	ko00790	3	47	477	13897	c125242.graph_c0;c126629.graph_c0;c86038.graph_c0	K03639+K18482+K13941
Steroid biosynthesis	ko00100	3	81	477	13897	c125112.graph_c0;c130196.graph_c0;c97018.graph_c0	K14423+K14423+K01853
Monoterpenoid biosynthesis	ko00902	2	6	477	13897	c102174.graph_c0;c122797.graph_c0	K15086+K15086
Valine, leucine and isoleucine biosynthesis	ko00290	2	58	477	13897	c123819.graph_c0;c93628.graph_c0	K00826+K01704
Ascorbate and aldarate metabolism	ko00053	2	134	477	13897	c119013.graph_c0;c128735.graph_c0	K00469+K00128
Pantothenate and CoA biosynthesis	ko00770	2	80	477	13897	c114123.graph_c0;c123819.graph_c0	K02201+K00826
Biotin metabolism	ko00780	2	39	477	13897	c100226.graph_c0;c132570.graph_c0	K09458+K00059
Inositol phosphate metabolism	ko00562	2	196	477	13897	c119013.graph_c0;c127088.graph_c0	K00469+K00889
Regulation of autophagy	ko04140	2	134	477	13897	c117024.graph_c0;c117516.graph_c0	K07198+K08269
One carbon pool by folate	ko00670	2	75	477	13897	c112704.graph_c0;c51284.graph_c0	K01934+K00600
Arachidonic acid metabolism	ko00590	2	51	477	13897	c127844.graph_c0;c98181.graph_c0	K18592+K01047
RNA polymerase	ko03020	2	150	477	13897	c124206.graph_c0;c125604.graph_c0	K03014+K03040
Sphingolipid metabolism	ko00600	2	111	477	13897	c116191.graph_c0;c133691.graph_c1	K00654+K04713

Carotenoid biosynthesis	ko00906	2	64	477	13897	c122440.graph_c0;c91224.graph_c0	K09840+K09843
Phosphatidylinositol signaling system	ko04070	2	194	477	13897	c112144.graph_c0;c127088.graph_c0	K02183+K00889
Lysine degradation	ko00310	2	136	477	13897	c109846.graph_c0;c128735.graph_c0	K14157+K00128
Proteasome	ko03050	2	264	477	13897	c121189.graph_c0;c88678.graph_c0	K03062+K02734
Taurine and hypotaurine metabolism	ko00430	1	26	477	13897	c127844.graph_c0	K18592
Non-homologous end-joining	ko03450	1	24	477	13897	c124068.graph_c0	K10885
Histidine metabolism	ko00340	1	81	477	13897	c128735.graph_c0	K00128
Degradation of aromatic compounds	ko01220	1	27	477	13897	c126578.graph_c0	K00121
Lysine biosynthesis	ko00300	1	42	477	13897	c114056.graph_c0	K10206
Glycosylphosphatidylinositol(GPI)-anchor biosynthesis	ko00563	1	58	477	13897	c117847.graph_c0	K05283
Homologous recombination	ko03440	1	157	477	13897	c118790.graph_c0	K10740
Mismatch repair	ko03430	1	132	477	13897	c118790.graph_c0	K10740

Other glycan degradation	ko00511	1	81	477	13897	c94189.graph_c0	K01191
Basal transcription factors	ko03022	1	135	477	13897	c133777.graph_c0	K03131
Aminoacyl-tRNA biosynthesis	ko00970	1	289	477	13897	c131414.graph_c0	K01866
Nicotinate and nicotinamide metabolism	ko00760	1	43	477	13897	c120459.graph_c0	K00278
Anthocyanin biosynthesis	ko00942	1	4	477	13897	c132750.graph_c0	K12930
C5-Branched dibasic acid metabolism	ko00660	1	28	477	13897	c93628.graph_c0	K01704
Selenocompound metabolism	ko00450	1	89	477	13897	c120855.graph_c0	K01761

Table S6. KEGG pathway classification of DEGs in PR vs RO comparison.

#Pathway	ko_ID	Unigene	gene	Unigene_all	gene_all		
#KEGG_n	477	KEGG_N	13897				
Starch and sucrose metabolism	ko00500	36	469	477	13897	c105513.graph_c1;c106004.graph_c0;c108042.graph_c0;c108790.graph_c0;c109671.graph_c0;c115711.graph_c0;c117587.graph_c0;c117822.graph_c0;c118113.graph_c0;c118816.graph_c0;c119141.graph_c0;c119325.graph_c0;c120576.graph_c0;c121742.graph_c0;c122721.graph_c0;c123210.graph_c0;c123220.graph_c0;c125155.graph_c1;c126580.graph_c0;c126811.graph_c0;c126934.graph_c0;c127107.graph_c0;c127792.graph_c0;c129616.graph_c0;c129750.graph_c0;c130158.graph_c0;c130187.graph_c0;c130727.graph_c1;c131002.graph_c1;c131646.graph_c1;c131799.graph_c0;c23686.graph_c0;c71123.graph_c0;c86535.graph_c0;c88845.graph_c0;c97687.graph_c0	K01188+K01188+K18447+K01188+K05350+K01176+K00695+K01051+K01188+K01188+K00696+K01184+K01051+K01051+K01835+K05350+K01188+K01176+K01177+K01193+K01187+K00847+K01188+K01051+K01051+K01087+K00847+K01176+K00696+K00688+K01176+K01177+K01051+K01188+K05350+K01188
Plant hormone signal transduction	ko04075	33	403	477	13897	c100991.graph_c0;c105815.graph_c0;c106087.graph_c0;c107593.graph_c0;c108427.graph_c0;c109272.graph_c0;c109646.graph_c0;c112138.graph_c0;c112179.graph_c0;c113017.graph_c0;c116573.graph_c0;c116665.graph_c0;c116968.graph_c0;c117784	K14487+K14505+K14490+K14492+K14490+K14492+K14488+K13464+K14488+K14484+K14505+K14488+K14432+K14487+K14488+K14496+K14488+K14488+K14497+K14498+K13464+K14484+K14505+K14490+K14512+K14491+K14491+K14

						.graph_c0;c120105.graph_c1;c120345.graph_c0;c120847.graph_c0;c121149.graph_c0;c121691.graph_c0;c121767.graph_c0;c122051.graph_c0;c122394.graph_c0;c123048.graph_c0;c124750.graph_c1;c124826.graph_c0;c125541.graph_c0;c127036.graph_c1;c127324.graph_c0;c127580.graph_c0;c127695.graph_c0;c130168.graph_c0;c132781.graph_c0;c133064.graph_c0	488+K14488+K14505+K14509+K14492+K14491
Phenylpropanoid biosynthesis	ko00940	29	250	477	13897	c101742.graph_c0;c105513.graph_c1;c106004.graph_c0;c107475.graph_c0;c108790.graph_c0;c109671.graph_c0;c111428.graph_c0;c111764.graph_c0;c114770.graph_c0;c116628.graph_c0;c117640.graph_c0;c118113.graph_c0;c118247.graph_c0;c118816.graph_c0;c120064.graph_c0;c120064.graph_c1;c123210.graph_c0;c123220.graph_c0;c125412.graph_c0;c127792.graph_c0;c129902.graph_c0;c130735.graph_c0;c133930.graph_c0;c50245.graph_c0;c86535.graph_c0;c86972.graph_c0;c88845.graph_c0;c89173.graph_c0;c97687.graph_c0	K00083+K01188+K01188+K00430+K01188+K05350+K00430+K11188+K13066+K00430+K12356+K01188+K00430+K01188+K13066+K13066+K05350+K01188+K01904+K01188+K13066+K00430+K00430+K00083+K01188+K11188+K05350+K09755+K01188
Ribosome	ko03010	24	996	477	13897	c102478.graph_c0;c102533.graph_c0;c105246.graph_c0;c108452.graph_c1;c111222.graph_c0;c114175.graph_c0;c118085.graph_c0;c119933.graph_c0;c122506.graph_c0	K02939+K02937+K02940+K02922+K02866+K02922+K02988+K02880+K02959+K02896+K02937+K02896+K02993+K02938+K02882+K02980+K02992+K02937+K0

						;c124830.graph_c0;c125171.graph_c0;c125852.graph_c0;c125958.graph_c0;c129136.graph_c0;c130486.graph_c0;c132355.graph_c0;c132848.graph_c0;c133030.graph_c0;c133694.graph_c0;c133984.graph_c0;c134599.graph_c0;c49761.graph_c0;c90571.graph_c0;c95596.graph_c0	2882+K02868+K02960+K02938+K02949+K02894
Biosynthesis of amino acids	ko01230	23	812	477	13897	c101979.graph_c0;c102878.graph_c0;c114056.graph_c0;c117589.graph_c0;c117781.graph_c1;c118697.graph_c0;c121090.graph_c0;c121536.graph_c0;c122230.graph_c0;c123819.graph_c0;c124334.graph_c0;c124635.graph_c0;c124834.graph_c0;c129927.graph_c0;c132024.graph_c0;c133226.graph_c0;c49644.graph_c0;c51012.graph_c0;c51284.graph_c0;c71676.graph_c0;c88948.graph_c0;c93628.graph_c0;c95528.graph_c0	K05359+K15227+K10206+K00620+K06001+K00611+K15227+K00815+K01695+K00826+K01738+K01915+K00615+K13832+K01738+K13832+K00030+K00789+K00600+K15227+K13034+K01704+K00891
Carbon metabolism	ko01200	22	955	477	13897	c100613.graph_c0;c102566.graph_c0;c110315.graph_c0;c118938.graph_c0;c120124.graph_c0;c123772.graph_c0;c124334.graph_c0;c124834.graph_c0;c126578.graph_c0;c128281.graph_c0;c128366.graph_c0;c129490.graph_c0;c132024.graph_c0;c134376.graph_c0;c134605.graph_c0;c49644.graph_c0;c51284.graph_c0;c62501.graph_c0;c	K00025+K00122+K00830+K05605+K01057+K00855+K01738+K00615+K00121+K01610+K01595+K00026+K01738+K01963+K01610+K00030+K00600+K01895+K00627+K01679+K13034+K01962

						85077.graph_c0;c88872.graph_c0;c88948.graph_c0;c90012.graph_c0	
Protein processing in endoplasmic reticulum	ko04141	22	868	477	13897	c102059.graph_c0;c108531.graph_c0;c109699.graph_c0;c115661.graph_c0;c117552.graph_c0;c118015.graph_c0;c118078.graph_c0;c120059.graph_c0;c121542.graph_c1;c122224.graph_c0;c122836.graph_c0;c123347.graph_c0;c124083.graph_c0;c125019.graph_c0;c125391.graph_c1;c129495.graph_c0;c131854.graph_c1;c23697.graph_c0;c71721.graph_c0;c90083.graph_c0;c97655.graph_c0;c97880.graph_c0	K13993+K03094+K13993+K13993+K03347+K13993+K13993+K03237+K13993+K13993+K13993+K03094+K09503+K13993+K13993+K08852+K03283+K03283+K13993+K13993+K03868+K06689
Purine metabolism	ko00230	19	563	477	13897	c108042.graph_c0;c110950.graph_c0;c111706.graph_c0;c112310.graph_c0;c112378.graph_c0;c117973.graph_c0;c120834.graph_c0;c122370.graph_c0;c122721.graph_c0;c124206.graph_c0;c125604.graph_c0;c128678.graph_c0;c129051.graph_c0;c130274.graph_c0;c130689.graph_c1;c131570.graph_c0;c131878.graph_c0;c64367.graph_c0;c99075.graph_c0	K18447+K10808+K00759+K11808+K00856+K06928+K01514+K00759+K01835+K03014+K03040+K10807+K02325+K01427+K00939+K01923+K10808+K02326+K00088
Plant-pathogen interaction	ko04626	19	373	477	13897	c112144.graph_c0;c112507.graph_c0;c115120.graph_c0;c119307.graph_c0;c119888.graph_c0;c121838.graph_c0;c122045.graph_c0;c125898.graph_c1;c129591.graph_c0;c130786.graph_c0;c131652.graph_c1;c13	K02183+K13448+K13448+K13412+K13412+K13459+K18875+K13457+K05391+K13429+K13420+K13412+K13447+K13447+K13457+K13459+K13448+K13412+K12795

						2441.graph_c0;c132982.graph_c1;c133136.graph_c0;c134348.graph_c0;c134380.graph_c0;c50350.graph_c0;c88346.graph_c0;c91463.graph_c0	
alpha-Linolenic acid metabolism	ko00592	18	130	477	13897	c102004.graph_c0;c102687.graph_c0;c109303.graph_c0;c109581.graph_c0;c115772.graph_c0;c117008.graph_c0;c118104.graph_c0;c118459.graph_c0;c120300.graph_c0;c121567.graph_c0;c123502.graph_c0;c123665.graph_c0;c125727.graph_c0;c133558.graph_c0;c134411.graph_c0;c134639.graph_c0;c23669.graph_c0;c98181.graph_c0	K18857+K00454+K08241+K18857+K01723+K10525+K18857+K01723+K05894+K01723+K00232+K10525+K08241+K00232+K00454+K00454+K00454+K01047
Photosynthesis	ko00195	17	144	477	13897	c100128.graph_c0;c101644.graph_c0;c104922.graph_c0;c105700.graph_c0;c108958.graph_c0;c109514.graph_c0;c109836.graph_c0;c111404.graph_c0;c120141.graph_c0;c124921.graph_c0;c125492.graph_c0;c126976.graph_c0;c128494.graph_c0;c130722.graph_c0;c132793.graph_c0;c95982.graph_c0;c97392.graph_c0	K02115+K02638+K02639+K08902+K02641+K02692+K02115+K14332+K02707+K02694+K02109+K02113+K02691+K02710+K02705+K02641+K02717
Oxidative phosphorylation	ko00190	15	552	477	13897	c100128.graph_c0;c105010.graph_c0;c109836.graph_c0;c112664.graph_c0;c120026.graph_c0;c121921.graph_c0;c125492.graph_c0;c126524.graph_c0;c126976.graph_c0	K02115+K02152+K02115+K01535+K05577+K02150+K02109+K05582+K02113+K03883+K03881+K02132+K05579+K01535+K01535

						;c129271.graph_c0;c132160.graph_c0;c132549.graph_c0;c133939.graph_c0;c28152.graph_c0;c71577.graph_c0	
Cyanoamino acid metabolism	ko00460	15	114	477	13897	c105513.graph_c1;c106004.graph_c0;c108790.graph_c0;c109671.graph_c0;c118113.graph_c0;c118816.graph_c0;c123210.graph_c0;c123220.graph_c0;c127792.graph_c0;c127844.graph_c0;c51284.graph_c0;c86535.graph_c0;c88845.graph_c0;c88948.graph_c0;c97687.graph_c0	K01188+K01188+K01188+K05350+K01188+K01188+K05350+K01188+K01188+K18592+K00600+K01188+K05350+K13034+K01188
Glutathione metabolism	ko00480	14	235	477	13897	c110950.graph_c0;c111372.graph_c0;c112965.graph_c0;c117302.graph_c0;c122681.graph_c0;c125111.graph_c0;c125826.graph_c0;c127844.graph_c0;c128678.graph_c0;c131878.graph_c0;c51066.graph_c0;c71042.graph_c0;c96247.graph_c0;c98149.graph_c0	K10808+K00799+K00799+K00797+K00799+K00799+K00799+K18592+K10807+K10808+K00799+K00799+K00799+K00799
Phenylalanine metabolism	ko00360	14	171	477	13897	c107475.graph_c0;c110715.graph_c0;c111428.graph_c0;c111764.graph_c0;c116628.graph_c0;c118247.graph_c0;c118423.graph_c0;c119172.graph_c0;c121536.graph_c0;c125412.graph_c0;c130735.graph_c0;c131042.graph_c0;c133930.graph_c0;c86972.	K00430+K07253+K00430+K11188+K00430+K00430+K00276+K00457+K00815+K01904+K00430+K00276+K00430+K11188

						graph_c0	
Ubiquitin mediated proteolysis	ko04120	14	468	477	13897	c100543.graph_c0;c108531.graph_c0;c108920.graph_c0;c115011.graph_c0;c117552.graph_c0;c119129.graph_c0;c123174.graph_c0;c123347.graph_c0;c123525.graph_c0;c128987.graph_c0;c131884.graph_c0;c133559.graph_c0;c97655.graph_c0;c97880.graph_c0	K10579+K03094+K10144+K10144+K03347+K10583+K03354+K03094+K03363+K10581+K12456+K10144+K03868+K06689
Cysteine and methionine metabolism	ko00270	13	289	477	13897	c100613.graph_c0;c117302.graph_c0;c120405.graph_c1;c120855.graph_c0;c121536.graph_c0;c123728.graph_c0;c124334.graph_c0;c125674.graph_c0;c125789.graph_c0;c129490.graph_c0;c132024.graph_c0;c51012.graph_c0;c88948.graph_c0	K00025+K00797+K00016+K01761+K00815+K01762+K01738+K00547+K05933+K00026+K01738+K00789+K13034
Glycolysis / Gluconeogenesis	ko00010	13	448	477	13897	c102004.graph_c0;c109581.graph_c0;c111937.graph_c0;c118104.graph_c0;c120405.graph_c1;c122721.graph_c0;c126578.graph_c0;c128281.graph_c0;c128735.graph_c0;c134543.graph_c0;c134605.graph_c0;c62501.graph_c0;c85077.graph_c0	K18857+K18857+K01785+K18857+K00016+K01835+K00121+K01610+K00128+K01568+K01610+K01895+K00627
Pyruvate metabolism	ko00620	12	357	477	13897	c100613.graph_c0;c120405.graph_c1;c128281.graph_c0;c128366.graph_c0;c128735.graph_c0;c129490.graph_c0;c134376.grap	K00025+K00016+K01610+K01595+K00128+K00026+K01963+K01610+K01895+K00627+K01679+K01962

						h_c0;c134605.graph_c0;c62501.graph_c0;c85077.graph_c0;c88872.graph_c0;c90012.graph_c0	
Tyrosine metabolism	ko00350	12	121	477	13897	c102004.graph_c0;c109581.graph_c0;c110715.graph_c0;c118104.graph_c0;c118423.graph_c0;c119172.graph_c0;c121536.graph_c0;c126578.graph_c0;c130866.graph_c0;c131042.graph_c0;c23725.graph_c0;c23725.graph_c1	K18857+K18857+K07253+K18857+K00276+K00457+K00815+K00121+K01592+K00276+K01592+K01592
Endocytosis	ko04144	11	472	477	13897	c108935.graph_c0;c111700.graph_c0;c112162.graph_c0;c114229.graph_c0;c118376.graph_c0;c127088.graph_c0;c131641.graph_c0;c131641.graph_c1;c131854.graph_c1;c133450.graph_c0;c23697.graph_c0	K07904+K01115+K07904+K12489+K01115+K00889+K12489+K12489+K03283+K01115+K03283
Cutin, suberine and wax biosynthesis	ko00073	11	60	477	13897	c124424.graph_c0;c124583.graph_c0;c127594.graph_c0;c128040.graph_c0;c131711.graph_c0;c132698.graph_c0;c133529.graph_c0;c86343.graph_c0;c88162.graph_c0;c97209.graph_c0;c99637.graph_c1	K15404+K15398+K17991+K13356+K15404+K15398+K13407+K15398+K13356+K13356+K13356
Pentose and glucuronate interconversions	ko00040	11	202	477	13897	c117822.graph_c0;c119325.graph_c0;c120576.graph_c0;c121742.graph_c0;c122856.graph_c0;c124896.graph_c0;c128735.graph_c0;c129616.graph_c0;c129750.graph_c0	K01051+K01184+K01051+K01051+K00011+K01728+K00128+K01051+K01051+K01728+K01051

						;c23628.graph_c0;c71123.graph_c0	
RNA degradation	ko03018	10	419	477	13897	c109491.graph_c0;c116941.graph_c0;c120290.graph_c0;c125901.graph_c0;c126315.graph_c0;c130826.graph_c0;c131538.graph_c1;c134322.graph_c0;c134441.graph_c0;c93753.graph_c0	K04077+K12606+K12608+K04077+K13126+K12603+K12619+K14442+K12619+K14442
Terpenoid backbone biosynthesis	ko00900	10	155	477	13897	c111454.graph_c0;c113927.graph_c0;c121595.graph_c0;c121595.graph_c1;c122587.graph_c0;c124462.graph_c0;c125410.graph_c0;c127762.graph_c1;c81933.graph_c0;c88832.graph_c0	K13789+K05356+K01662+K01662+K15889+K13789+K10960+K11778+K00021+K00021
Phenylalanine, tyrosine and tryptophan biosynthesis	ko00400	10	117	477	13897	c101979.graph_c0;c102878.graph_c0;c117781.graph_c1;c121090.graph_c0;c121536.graph_c0;c122230.graph_c0;c129927.graph_c0;c133226.graph_c0;c71676.graph_c0;c95528.graph_c0	K05359+K15227+K06001+K15227+K00815+K01695+K13832+K13832+K15227+K00891
Amino sugar and nucleotide sugar metabolism	ko00520	10	335	477	13897	c106554.graph_c0;c122721.graph_c0;c123063.graph_c0;c124607.graph_c0;c127107.graph_c0;c130187.graph_c0;c131811.graph_c0;c54775.graph_c0;c88590.graph_c0;c89832.graph_c0	K01183+K01835+K01183+K01209+K00847+K00847+K01183+K01784+K01784+K12449
Fatty acid metabolism	ko01212	10	268	477	13897	c100226.graph_c0;c108824.graph_c0;c115084.graph_c0;c121978.graph_c0;c123502.graph_c0;c124335.graph_c0;c132570.graph_c0;c133558.graph_c0;c134376.graph_c0;c90012.graph_c0	K09458+K01074+K10251+K10257+K00232+K10781+K00059+K00232+K01963+K01962

Pyrimidine metabolism	ko00240	10	456	477	13897	c102867.graph_c0;c110950.graph_c0;c114780.graph_c0;c124206.graph_c0;c125604.graph_c0;c128678.graph_c0;c129051.graph_c0;c131878.graph_c0;c64367.graph_c0;c93528.graph_c0	K00857+K10808+K00876+K03014+K03040+K10807+K02325+K10808+K02326+K01520
Glycerophospholipid metabolism	ko00564	9	243	477	13897	c105872.graph_c0;c111700.graph_c0;c118376.graph_c0;c124631.graph_c0;c127052.graph_c0;c128220.graph_c0;c130294.graph_c0;c133450.graph_c0;c98181.graph_c0	K06130+K01115+K01115+K06129+K13508+K13513+K13508+K01115+K01047
Galactose metabolism	ko00052	9	166	477	13897	c111937.graph_c0;c121255.graph_c1;c122721.graph_c0;c122856.graph_c0;c126811.graph_c0;c126934.graph_c0;c127648.graph_c0;c54775.graph_c0;c88590.graph_c0	K01785+K06617+K01835+K00011+K01193+K01187+K18819+K01784+K01784
Peroxisome	ko04146	9	287	477	13897	c110315.graph_c0;c123153.graph_c0;c123502.graph_c0;c128040.graph_c0;c129908.graph_c0;c133558.graph_c0;c88162.graph_c0;c97209.graph_c0;c99637.graph_c1	K00830+K13346+K00232+K13356+K11517+K00232+K13356+K13356+K13356
RNA transport	ko03013	8	630	477	13897	c102019.graph_c0;c116762.graph_c0;c117960.graph_c0;c120059.graph_c0;c125991.graph_c0;c126315.graph_c0;c130438.graph_c0;c131752.graph_c0	K12882+K03257+K03260+K03237+K03231+K13126+K14293+K03250
Thiamine metabolism	ko00730	8	45	477	13897	c112666.graph_c0;c117167.graph_c0;c117973.graph_c0;c121244.graph_c0;c121595.graph_c0;c121595.graph_c1;c127214.graph_c1;c127921.graph_c0	K03146+K04487+K06928+K03146+K01662+K01662+K04487+K00878
Glyoxylate and	ko00630	8	252	477	13897	c100613.graph_c0;c102566.graph_c0;c110	K00025+K00122+K00830+K01915+K000

dicarboxylate metabolism						315.graph_c0;c124635.graph_c0;c129490.graph_c0;c129908.graph_c0;c51284.graph_c0;c96010.graph_c0	26+K11517+K00600+K02437
Phagosome	ko04145	7	348	477	13897	c105010.graph_c0;c113632.graph_c0;c121921.graph_c0;c123483.graph_c0;c132440.graph_c0;c70889.graph_c0;c97672.graph_c0	K02152+K07375+K02150+K04392+K08492+K07374+K07375
mRNA surveillance pathway	ko03015	7	452	477	13897	c102019.graph_c0;c118482.graph_c0;c124542.graph_c0;c126315.graph_c0;c129634.graph_c0;c132292.graph_c2;c88662.graph_c0	K12882+K03456+K14400+K13126+K03265+K03456+K03265
Fructose and mannose metabolism	ko00051	7	201	477	13897	c108042.graph_c0;c114913.graph_c0;c118742.graph_c0;c122856.graph_c0;c127107.graph_c0;c130187.graph_c0;c130978.graph_c0	K18447+K00895+K00895+K00011+K00847+K00847+K00895
Zeatin biosynthesis	ko00908	7	19	477	13897	c124810.graph_c0;c125250.graph_c0;c129356.graph_c0;c130317.graph_c0;c130652.graph_c1;c131511.graph_c0;c132025.graph_c0	K00279+K10760+K13495+K00279+K10717+K00791+K00279
Fatty acid degradation	ko00071	7	197	477	13897	c102004.graph_c0;c109581.graph_c0;c118104.graph_c0;c123502.graph_c0;c126578.graph_c0;c128735.graph_c0;c133558.graph_c0	K18857+K18857+K18857+K00232+K00121+K00128+K00232
Glycine, serine and threonine metabolism	ko00260	7	226	477	13897	c110315.graph_c0;c117781.graph_c1;c118423.graph_c0;c122230.graph_c0;c131042.graph_c0;c51284.graph_c0;c96010.graph_	K00830+K06001+K00276+K01695+K00276+K00600+K02437

						c0	
Citrate cycle (TCA cycle)	ko00020	7	304	477	13897	c100613.graph_c0;c128281.graph_c0;c129490.graph_c0;c134605.graph_c0;c49644.graph_c0;c85077.graph_c0;c88872.graph_c0	K00025+K01610+K00026+K01610+K00030+K00627+K01679
Carbon fixation in photosynthetic organisms	ko00710	7	282	477	13897	c100613.graph_c0;c123772.graph_c0;c124834.graph_c0;c128281.graph_c0;c128366.graph_c0;c129490.graph_c0;c134605.graph_c0	K00025+K00855+K00615+K01610+K01595+K00026+K01610
Isoquinoline alkaloid biosynthesis	ko00950	6	54	477	13897	c118423.graph_c0;c121536.graph_c0;c130866.graph_c0;c131042.graph_c0;c23725.graph_c0;c23725.graph_c1	K00276+K00815+K01592+K00276+K01592+K01592
Porphyrin and chlorophyll metabolism	ko00860	6	131	477	13897	c121882.graph_c0;c123884.graph_c0;c125410.graph_c0;c127890.graph_c0;c127890.graph_c1;c130025.graph_c0	K04035+K00218+K10960+K03403+K03403+K13071
ABC transporters	ko02010	6	155	477	13897	c119157.graph_c0;c126614.graph_c0;c130859.graph_c0;c131196.graph_c0;c132418.graph_c0;c134352.graph_c0	K05658+K05658+K05658+K05658+K05658+K05658
Base excision repair	ko03410	6	137	477	13897	c110971.graph_c0;c121800.graph_c0;c124043.graph_c0;c129051.graph_c0;c64367.graph_c0;c97798.graph_c0	K10798+K01142+K10798+K02325+K02326+K10798
Arginine and proline metabolism	ko00330	6	287	477	13897	c117302.graph_c0;c117589.graph_c0;c118697.graph_c0;c124635.graph_c0;c128735.graph_c0;c130274.graph_c0	K00797+K00620+K00611+K01915+K00128+K01427
Photosynthesis - antenna proteins	ko00196	6	52	477	13897	c100527.graph_c0;c106848.graph_c0;c109381.graph_c0;c114287.graph_c0;c71681.g	K08910+K08909+K08916+K08908+K08913+K08913

						raph_c0;c89965.graph_c0	
Circadian rhythm - plant	ko04712	6	88	477	13897	c112186.graph_c0;c126452.graph_c1;c127832.graph_c0;c130328.graph_c0;c132912.graph_c0;c93168.graph_c0	K16166+K12130+K16241+K12129+K12129+K00660
Spliceosome	ko03040	6	868	477	13897	c102019.graph_c0;c120047.graph_c0;c131854.graph_c1;c23697.graph_c0;c71357.graph_c0;c91908.graph_c0	K12882+K12830+K03283+K03283+K12833+K12833
Brassinosteroid biosynthesis	ko00905	5	20	477	13897	c105790.graph_c0;c118316.graph_c0;c119899.graph_c0;c120722.graph_c0;c97361.graph_c0	K12637+K09587+K12638+K15639+K15639
Biosynthesis of unsaturated fatty acids	ko01040	5	111	477	13897	c115084.graph_c0;c121978.graph_c0;c123502.graph_c0;c132570.graph_c0;c133558.graph_c0	K10251+K10257+K00232+K00059+K00232
Linoleic acid metabolism	ko00591	5	39	477	13897	c102687.graph_c0;c134411.graph_c0;c134639.graph_c0;c23669.graph_c0;c98181.graph_c0	K00454+K00454+K00454+K00454+K01047
beta-Alanine metabolism	ko00410	5	176	477	13897	c117302.graph_c0;c118423.graph_c0;c118938.graph_c0;c128735.graph_c0;c131042.graph_c0	K00797+K00276+K05605+K00128+K00276
Propanoate metabolism	ko00640	5	140	477	13897	c118938.graph_c0;c120405.graph_c1;c134376.graph_c0;c62501.graph_c0;c90012.graph_c0	K05605+K00016+K01963+K01895+K01962
Glycerolipid metabolism	ko00561	5	191	477	13897	c122856.graph_c0;c127052.graph_c0;c128220.graph_c0;c128735.graph_c0;c130294.graph_c0	K00011+K13508+K13513+K00128+K13508
Nitrogen	ko00910	5	100	477	13897	c107127.graph_c0;c112691.graph_c0;c122	K01674+K01725+K01673+K01915+K025

metabolism						099.graph_c0;c124635.graph_c0;c130648.graph_c0	75
Fatty acid biosynthesis	ko00061	5	136	477	13897	c100226.graph_c0;c124335.graph_c0;c132570.graph_c0;c134376.graph_c0;c90012.graph_c0	K09458+K10781+K00059+K01963+K01962
Fatty acid elongation	ko00062	4	86	477	13897	c101374.graph_c0;c108824.graph_c0;c115084.graph_c0;c70542.graph_c0	K15397+K01074+K10251+K15397
Ether lipid metabolism	ko00565	4	87	477	13897	c111700.graph_c0;c118376.graph_c0;c133450.graph_c0;c98181.graph_c0	K01115+K01115+K01115+K01047
Ubiquinone and other terpenoid-quinone biosynthesis	ko00130	4	74	477	13897	c119172.graph_c0;c121536.graph_c0;c123577.graph_c0;c125412.graph_c0	K00457+K00815+K02552+K01904
DNA replication	ko03030	4	216	477	13897	c118790.graph_c0;c121683.graph_c0;c129051.graph_c0;c64367.graph_c0	K10740+K10745+K02325+K02326
Nucleotide excision repair	ko03420	4	225	477	13897	c118790.graph_c0;c129051.graph_c0;c64367.graph_c0;c97655.graph_c0	K10740+K02325+K02326+K03868
Alanine, aspartate and glutamate metabolism	ko00250	4	240	477	13897	c110315.graph_c0;c120459.graph_c0;c124635.graph_c0;c130166.graph_c0	K00830+K00278+K01915+K01953
2-Oxocarboxylic acid metabolism	ko01210	4	216	477	13897	c117589.graph_c0;c123819.graph_c0;c49644.graph_c0;c93628.graph_c0	K00620+K00826+K00030+K01704
Ribosome biogenesis in eukaryotes	ko03008	4	379	477	13897	c116289.graph_c0;c122033.graph_c0;c131538.graph_c1;c134441.graph_c0	K14521+K14538+K12619+K12619
SNARE interactions	ko04130	4	99	477	13897	c109420.graph_c0;c113095.graph_c0;c122	K08486+K08506+K08486+K08492

in vesicular transport						232.graph_c1;c132440.graph_c0	
Flavonoid biosynthesis	ko00941	4	64	477	13897	c105653.graph_c0;c126380.graph_c0;c93168.graph_c0;c94554.graph_c0	K01859+K13082+K00660+K05278
Tropane, piperidine and pyridine alkaloid biosynthesis	ko00960	4	67	477	13897	c118423.graph_c0;c121536.graph_c0;c122496.graph_c0;c131042.graph_c0	K00276+K00815+K08081+K00276
Pentose phosphate pathway	ko00030	3	196	477	13897	c120124.graph_c0;c122721.graph_c0;c124834.graph_c0	K01057+K01835+K00615
Valine, leucine and isoleucine degradation	ko00280	3	223	477	13897	c118938.graph_c0;c123819.graph_c0;c128735.graph_c0	K05605+K00826+K00128
Riboflavin metabolism	ko00740	3	26	477	13897	c112874.graph_c0;c125704.graph_c0;c129304.graph_c0	K14652+K14379+K14652
Sulfur relay system	ko04122	3	37	477	13897	c117167.graph_c0;c125242.graph_c0;c127214.graph_c1	K04487+K03639+K04487
Sulfur metabolism	ko00920	3	92	477	13897	c124334.graph_c0;c132024.graph_c0;c88948.graph_c0	K01738+K01738+K13034
Tryptophan metabolism	ko00380	3	126	477	13897	c125003.graph_c0;c127788.graph_c0;c128735.graph_c0	K07408+K11816+K00128
Diterpenoid biosynthesis	ko00904	3	25	477	13897	c113336.graph_c0;c130968.graph_c0;c89332.graph_c0	K04125+K04121+K17982
Folate biosynthesis	ko00790	3	47	477	13897	c125242.graph_c0;c126629.graph_c0;c86038.graph_c0	K03639+K18482+K13941
Steroid	ko00100	3	81	477	13897	c125112.graph_c0;c130196.graph_c0;c970	K14423+K14423+K01853

biosynthesis						18.graph_c0	
Monoterpenoid biosynthesis	ko00902	2	6	477	13897	c102174.graph_c0;c122797.graph_c0	K15086+K15086
Valine, leucine and isoleucine biosynthesis	ko00290	2	58	477	13897	c123819.graph_c0;c93628.graph_c0	K00826+K01704
Ascorbate and aldarate metabolism	ko00053	2	134	477	13897	c119013.graph_c0;c128735.graph_c0	K00469+K00128
Pantothenate and CoA biosynthesis	ko00770	2	80	477	13897	c114123.graph_c0;c123819.graph_c0	K02201+K00826
Biotin metabolism	ko00780	2	39	477	13897	c100226.graph_c0;c132570.graph_c0	K09458+K00059
Inositol phosphate metabolism	ko00562	2	196	477	13897	c119013.graph_c0;c127088.graph_c0	K00469+K00889
Regulation of autophagy	ko04140	2	134	477	13897	c117024.graph_c0;c117516.graph_c0	K07198+K08269
One carbon pool by folate	ko00670	2	75	477	13897	c112704.graph_c0;c51284.graph_c0	K01934+K00600
Arachidonic acid metabolism	ko00590	2	51	477	13897	c127844.graph_c0;c98181.graph_c0	K18592+K01047
RNA polymerase	ko03020	2	150	477	13897	c124206.graph_c0;c125604.graph_c0	K03014+K03040
Sphingolipid metabolism	ko00600	2	111	477	13897	c116191.graph_c0;c133691.graph_c1	K00654+K04713
Carotenoid biosynthesis	ko00906	2	64	477	13897	c122440.graph_c0;c91224.graph_c0	K09840+K09843
Phosphatidylinosi	ko04070	2	194	477	13897	c112144.graph_c0;c127088.graph_c0	K02183+K00889

tol signaling system							
Lysine degradation	ko00310	2	136	477	13897	c109846.graph_c0;c128735.graph_c0	K14157+K00128
Proteasome	ko03050	2	264	477	13897	c121189.graph_c0;c88678.graph_c0	K03062+K02734
Taurine and hypotaurine metabolism	ko00430	1	26	477	13897	c127844.graph_c0	K18592
Non-homologous end-joining	ko03450	1	24	477	13897	c124068.graph_c0	K10885
Histidine metabolism	ko00340	1	81	477	13897	c128735.graph_c0	K00128
Degradation of aromatic compounds	ko01220	1	27	477	13897	c126578.graph_c0	K00121
Lysine biosynthesis	ko00300	1	42	477	13897	c114056.graph_c0	K10206
Glycosylphosphatidylinositol(GPI)-anchor biosynthesis	ko00563	1	58	477	13897	c117847.graph_c0	K05283
Homologous recombination	ko03440	1	157	477	13897	c118790.graph_c0	K10740
Mismatch repair	ko03430	1	132	477	13897	c118790.graph_c0	K10740
Other glycan degradation	ko00511	1	81	477	13897	c94189.graph_c0	K01191
Basal	ko03022	1	135	477	13897	c133777.graph_c0	K03131

transcription factors							
Aminoacyl-tRNA biosynthesis	ko00970	1	289	477	13897	c131414.graph_c0	K01866
Nicotinate and nicotinamide metabolism	ko00760	1	43	477	13897	c120459.graph_c0	K00278
Anthocyanin biosynthesis	ko00942	1	4	477	13897	c132750.graph_c0	K12930
C5-Branched dibasic acid metabolism	ko00660	1	28	477	13897	c93628.graph_c0	K01704
Selenocompound metabolism	ko00450	1	89	477	13897	c120855.graph_c0	K01761

Table S7. KEGG pathway classification of DEGs in PO vs RO comparison.

#Pathway	ko_ID	Unigene	gene	Unigene_all	gene_all		
#KEGG_n		KEGG_N	13897				
Starch and sucrose metabolism	ko00500	37	469	408	13897	c112594.graph_c0;c113248.graph_c0;c115504.graph_c0;c115711.graph_c0;c117587.graph_c0;c117851.graph_c0;c117966.graph_c0;c118113.graph_c0;c118816.graph_c0;c119325.graph_c0;c120249.graph_c0;c120576.graph_c0;c120604.graph_c0;c121742.graph_c0;c121995.graph_c0;c124410.graph_c0;c125113.graph_c0;c125202.graph_c0;c126509.graph_c1;c126580.graph_c0;c126605.graph_c0;c127309.graph_c0;c127792.graph_c0;c128185.graph_c0;c128547.graph_c1;c129616.graph_c0;c130060.graph_c0;c130684.graph_c0;c131799.graph_c0;c132238.graph_c0;c132359.graph_c1;c23686.graph_c0;c71123.graph_c0;c86535.graph_c0;c87790.graph_c0;c94374.graph_c0;c97687.graph_c0	K01213+K00012+K01177+K01176+K00695+K01051+K01051+K01188+K01188+K01184+K00844+K01051+K01188+K01051+K01188+K01051+K01213+K01051+K00012+K01177+K00975+K05349+K01188+K00705+K01188+K01051+K01087+K01193+K01176+K00696+K01051+K01177+K01051+K01188+K01051+K01051+K01188
Plant hormone signal transduction	ko04075	26	403	408	13897	c100991.graph_c0;c102167.graph_c0;c104874.graph_c0;c105109.graph_c0;c110148.graph_c0;c112179.graph_c0;c112371.graph_c0;c112578.graph_c0;c112898.graph_c0;c113017.graph_c0;c116665.graph_c0;c117096.graph_c0;c117362.graph_c0;c120105.graph_c1;c1	K14487+K14488+K14488+K14497+K14490+K14488+K14488+K14492+K14516+K14484+K14488+K14496+K14498+K14488+K14496+K14488+K14488+K14512+K14488+K16189+K14432+K14432+K14515+K14509+K1

						20345.graph_c0;c120546.graph_c0;c120847.graph_c0;c124826.graph_c0;c127580.graph_c0;c127688.graph_c0;c131060.graph_c0;c131060.graph_c3;c131976.graph_c0;c132990.graph_c0;c88326.graph_c0;c90004.graph_c0	4488+K14496
Phenylpropanoid biosynthesis	ko00940	25	250	408	13897	c101742.graph_c0;c101742.graph_c1;c111410.graph_c0;c111428.graph_c0;c114770.graph_c0;c117640.graph_c0;c118113.graph_c0;c118816.graph_c0;c119082.graph_c0;c120604.graph_c0;c121941.graph_c0;c121995.graph_c0;c125198.graph_c0;c125906.graph_c0;c127309.graph_c0;c127792.graph_c0;c128306.graph_c0;c128547.graph_c1;c129902.graph_c0;c133129.graph_c0;c133930.graph_c0;c50245.graph_c0;c86535.graph_c0;c89173.graph_c0;c97687.graph_c0	K00083+K00083+K13065+K00430+K13066+K12356+K01188+K01188+K01904+K01188+K00430+K01188+K00430+K00430+K05349+K01188+K00083+K01188+K13066+K10775+K00430+K00083+K01188+K09755+K01188
Pentose and glucuronate interconversions	ko00040	25	202	408	13897	c102361.graph_c0;c102552.graph_c0;c112594.graph_c0;c112781.graph_c0;c113248.graph_c0;c116799.graph_c0;c117851.graph_c0;c117966.graph_c0;c119325.graph_c0;c120576.graph_c0;c121742.graph_c0;c122856.graph_c0;c123853.graph_c0;c124325.graph_c0;c124325.graph_c1;c124410.graph_c0;c125113.graph_c0;c125202.graph_c0;c126509.graph_c1;c129616.graph_c0;c132359.graph_c1;c23628.graph_c0;c71123.graph_c0;c87790.graph_c0;c94374.graph_c0	K01728+K01728+K01213+K01728+K00012+K01728+K01051+K01051+K01184+K01051+K01051+K00011+K01728+K01728+K01728+K01051+K01213+K01051+K00012+K01051+K01051+K01728+K01051+K01051+K01051
Carbon metabolism	ko01200	24	955	408	13897	c106243.graph_c0;c112914.graph_c0;c114199.graph_c0;c118228.graph_c1;c118938.graph_c0;c119204.gra	K00134+K00134+K01100+K00121+K05605+K05298+K01057+K00844+K01895+K0087

						ph_c0;c119582.graph_c1;c120249.graph_c0;c120295.graph_c0;c123524.graph_c0;c123772.graph_c0;c124334.graph_c0;c124834.graph_c0;c129153.graph_c0;c129490.graph_c0;c130262.graph_c0;c132024.graph_c0;c133476.graph_c0;c134598.graph_c0;c134605.graph_c0;c49644.graph_c0;c50718.graph_c0;c51688.graph_c0;c62501.graph_c0	3+K00855+K01738+K00615+K01689+K00026+K13034+K01738+K01681+K01738+K01610+K00030+K01057+K01057+K01895
Plant-pathogen interaction	ko04626	18	373	408	13897	c100506.graph_c0;c105088.graph_c0;c112144.graph_c0;c112507.graph_c0;c114370.graph_c0;c118619.graph_c0;c118881.graph_c0;c122045.graph_c0;c125898.graph_c1;c128389.graph_c0;c128760.graph_c0;c131119.graph_c0;c131119.graph_c1;c132003.graph_c1;c132441.graph_c0;c132784.graph_c0;c134348.graph_c0;c91463.graph_c0	K13448+K13448+K02183+K13448+K04368+K13448+K13448+K18875+K13457+K13447+K13412+K13412+K13412+K18835+K13412+K13447+K13457+K12795
Photosynthesis	ko00195	18	144	408	13897	c101596.graph_c0;c101644.graph_c0;c102008.graph_c0;c102091.graph_c0;c102177.graph_c0;c105700.graph_c0;c107088.graph_c0;c111404.graph_c0;c111951.graph_c1;c120594.graph_c0;c122529.graph_c0;c124921.graph_c0;c131924.graph_c1;c132793.graph_c0;c50756.graph_c0;c71464.graph_c0;c95982.graph_c0;c97392.graph_c0	K02701+K02638+K02639+K02698+K02723+K08902+K03542+K14332+K02641+K02113+K02109+K02694+K02693+K02705+K02721+K08905+K02641+K02717
Biosynthesis of amino acids	ko01230	18	812	408	13897	c100821.graph_c0;c106243.graph_c0;c112408.graph_c0;c112914.graph_c0;c121031.graph_c0;c122230.graph_c0;c123524.graph_c0;c124334.graph_c0;c124834.graph_c0;c125451.graph_c1;c129153.graph_c0;c129927.graph_c0;c130262.graph_c0;c132024.graph_c0;c1	K00891+K00134+K13832+K00134+K01817+K01695+K00873+K01738+K00615+K13832+K01689+K13832+K13034+K01738+K13832+K01681+K01738+K00030

						33161.graph_c0;c133476.graph_c0;c134598.graph_c0;c49644.graph_c0	
Ribosome	ko03010	15	996	408	13897	c100604.graph_c0;c102533.graph_c0;c106099.graph_c0;c108526.graph_c1;c110436.graph_c0;c111222.graph_c0;c118085.graph_c0;c119933.graph_c0;c123322.graph_c0;c123949.graph_c0;c124097.graph_c0;c55594.graph_c0;c74451.graph_c0;c77479.graph_c0;c90571.graph_c0	K02977+K02937+K02962+K02979+K02870+K02866+K02988+K02880+K02889+K02932+K02988+K02960+K02949+K02960+K02949
Glutathione metabolism	ko00480	15	235	408	13897	c101895.graph_c0;c105192.graph_c0;c105273.graph_c0;c108187.graph_c0;c111372.graph_c0;c112220.graph_c0;c113610.graph_c0;c117370.graph_c0;c119814.graph_c0;c125111.graph_c0;c125826.graph_c0;c133973.graph_c0;c51066.graph_c0;c98149.graph_c0;c98296.graph_c0	K00799+K00799+K01919+K00432+K00799+K00799+K00799+K00799+K00799+K00799+K00799+K01919+K00799+K00799+K00799
Protein processing in endoplasmic reticulum	ko04141	15	868	408	13897	c102704.graph_c0;c108317.graph_c0;c109348.graph_c0;c110519.graph_c0;c112831.graph_c0;c115728.graph_c0;c117552.graph_c0;c121542.graph_c1;c125404.graph_c0;c130072.graph_c0;c131510.graph_c0;c131834.graph_c0;c23599.graph_c0;c52034.graph_c0;c93826.graph_c0	K03094+K03283+K09490+K03283+K09503+K03094+K03347+K13993+K10575+K03283+K03283+K03283+K03283+K13993+K13993
Glycolysis / Gluconeogenesis	ko00010	14	448	408	13897	c106243.graph_c0;c108564.graph_c0;c109130.graph_c0;c112914.graph_c0;c118104.graph_c0;c118228.graph_c1;c120249.graph_c0;c120295.graph_c0;c123524.graph_c0;c129153.graph_c0;c132004.graph_c0;c132004.graph_c1;c134605.graph_c0;c62501.graph_c0	K00134+K00001+K01792+K00134+K18857+K00121+K00844+K01895+K00873+K01689+K01785+K01785+K01610+K01895
Spliceosome	ko030	14	868	408	13897	c100425.graph_c0;c102019.graph_c0;c108164.graph_	K12874+K12882+K12874+K03283+K03283

	40					c0;c108317.graph_c0;c110519.graph_c0;c120921.graph_c0;c130072.graph_c0;c130121.graph_c0;c131510.graph_c0;c131834.graph_c0;c133691.graph_c0;c134621.graph_c0;c23599.graph_c0;c91908.graph_c0	+K12874+K03283+K12830+K03283+K03283+K12833+K12882+K03283+K12833
RNA degradation	ko03018	12	419	408	13897	c106929.graph_c0;c115207.graph_c0;c115874.graph_c0;c116900.graph_c0;c116941.graph_c0;c120023.graph_c0;c120290.graph_c0;c122821.graph_c0;c126899.graph_c0;c128434.graph_c0;c129153.graph_c0;c93753.graph_c0	K12604+K12608+K12605+K12619+K12606+K13126+K12608+K12581+K13126+K12580+K01689+K14442
Cutin, suberine and wax biosynthesis	ko00073	11	60	408	13897	c100231.graph_c0;c119134.graph_c1;c124147.graph_c0;c124424.graph_c0;c128040.graph_c0;c131711.graph_c0;c132698.graph_c0;c133529.graph_c0;c23649.graph_c0;c97209.graph_c0;c99637.graph_c1	K13356+K15400+K13356+K15404+K13356+K15404+K15398+K13407+K13356+K13356+K13356
Endocytosis	ko04144	11	472	408	13897	c108317.graph_c0;c110519.graph_c0;c112162.graph_c0;c114229.graph_c0;c121339.graph_c0;c130072.graph_c0;c131510.graph_c0;c131641.graph_c0;c131641.graph_c1;c131834.graph_c0;c23599.graph_c0	K03283+K03283+K07904+K12489+K01115+K03283+K03283+K12489+K12489+K03283+K03283
Photosynthesis - antenna proteins	ko00196	10	52	408	13897	c100527.graph_c0;c105640.graph_c0;c106848.graph_c0;c109210.graph_c0;c109381.graph_c0;c114287.graph_c0;c123544.graph_c0;c23604.graph_c0;c46372.graph_c0;c89853.graph_c0	K08910+K08917+K08909+K08912+K08916+K08908+K08912+K08913+K08913+K08908
Galactose metabolism	ko00052	10	166	408	13897	c102094.graph_c0;c120249.graph_c0;c122856.graph_c0;c123062.graph_c0;c124235.graph_c0;c127648.graph_c0;c130684.graph_c0;c132004.graph_c0;c132004.	K18819+K00844+K00011+K01784+K01784+K18819+K01193+K01785+K01785+K06617

						graph_c1;c49856.graph_c0	
Cyanoamino acid metabolism	ko00460	10	114	408	13897	c118113.graph_c0;c118816.graph_c0;c120604.graph_c0;c121995.graph_c0;c127309.graph_c0;c127792.graph_c0;c128547.graph_c1;c130262.graph_c0;c86535.graph_c0;c97687.graph_c0	K01188+K01188+K01188+K01188+K05349+K01188+K01188+K13034+K01188+K01188
RNA transport	ko03013	9	630	408	13897	c102019.graph_c0;c113016.graph_c1;c116762.graph_c0;c120023.graph_c0;c120228.graph_c0;c126899.graph_c0;c130438.graph_c0;c134621.graph_c0;c97352.graph_c0	K12882+K07936+K03257+K13126+K03113+K13126+K14293+K12882+K12160
alpha-Linolenic acid metabolism	ko00592	9	130	408	13897	c102687.graph_c0;c115680.graph_c0;c118104.graph_c0;c118459.graph_c0;c120300.graph_c0;c123502.graph_c0;c123665.graph_c0;c133558.graph_c0;c134411.graph_c0	K00454+K10525+K18857+K01723+K05894+K00232+K10525+K00232+K00454
Peroxisome	ko04146	9	287	408	13897	c100231.graph_c0;c123502.graph_c0;c124147.graph_c0;c128040.graph_c0;c133558.graph_c0;c23649.graph_c0;c71372.graph_c0;c97209.graph_c0;c99637.graph_c1	K13356+K00232+K13356+K13356+K00232+K13356+K01897+K13356+K13356
Phenylalanine metabolism	ko00360	9	171	408	13897	c111428.graph_c0;c119082.graph_c0;c121941.graph_c0;c122229.graph_c0;c125198.graph_c0;c125906.graph_c0;c131042.graph_c0;c133129.graph_c0;c133930.graph_c0	K00430+K01904+K00430+K07253+K00430+K00430+K00276+K10775+K00430
Cysteine and methionine metabolism	ko00270	9	289	408	13897	c108515.graph_c0;c112512.graph_c0;c120855.graph_c0;c124334.graph_c0;c129490.graph_c0;c130262.graph_c0;c131509.graph_c0;c132024.graph_c0;c134598.graph_c0	K05933+K08967+K01761+K01738+K00026+K13034+K01244+K01738+K01738
Carbon fixation in	ko007	8	282	408	13897	c106243.graph_c0;c112914.graph_c0;c114199.graph_	K00134+K00134+K01100+K05298+K00855

photosynthetic organisms	10					c0;c119204.graph_c0;c123772.graph_c0;c124834.graph_c0;c129490.graph_c0;c134605.graph_c0	+K00615+K00026+K01610
Fatty acid metabolism	ko01212	8	268	408	13897	c100226.graph_c0;c108824.graph_c0;c111947.graph_c0;c115084.graph_c0;c123502.graph_c0;c124335.graph_c0;c133558.graph_c0;c71372.graph_c0	K09458+K01074+K10256+K10251+K00232+K10781+K00232+K01897
Glycerophospholipid metabolism	ko00564	7	243	408	13897	c121339.graph_c0;c121925.graph_c0;c122346.graph_c0;c124631.graph_c0;c130294.graph_c0;c70983.graph_c0;c93485.graph_c0	K01115+K06130+K01114+K06129+K13508+K13508+K06130
Terpenoid backbone biosynthesis	ko00900	7	155	408	13897	c108010.graph_c1;c108150.graph_c0;c112284.graph_c0;c121595.graph_c0;c121595.graph_c1;c125400.graph_c0;c133728.graph_c1	K00787+K01662+K01641+K01662+K01662+K11778+K00021
Zeatin biosynthesis	ko00908	7	19	408	13897	c124021.graph_c0;c125250.graph_c0;c129356.graph_c0;c131011.graph_c0;c131511.graph_c0;c132025.graph_c0;c93206.graph_c0	K10760+K10760+K13495+K00279+K00791+K00279+K13495
Tyrosine metabolism	ko00350	7	121	408	13897	c108564.graph_c0;c118104.graph_c0;c118228.graph_c1;c122229.graph_c0;c123894.graph_c0;c130866.graph_c0;c131042.graph_c0	K00001+K18857+K00121+K07253+K01592+K01592+K00276
Phenylalanine, tyrosine and tryptophan biosynthesis	ko00400	7	117	408	13897	c100821.graph_c0;c112408.graph_c0;c121031.graph_c0;c122230.graph_c0;c125451.graph_c1;c129927.graph_c0;c133161.graph_c0	K00891+K13832+K01817+K01695+K13832+K13832+K13832
Oxidative phosphorylation	ko00190	7	552	408	13897	c112402.graph_c0;c120594.graph_c0;c121111.graph_c0;c122529.graph_c0;c133150.graph_c1;c50116.graph_c0;c96666.graph_c0	K01535+K02113+K03934+K02109+K03935+K01535+K01507
Ubiquitin mediated proteolysis	ko04120	7	468	408	13897	c102704.graph_c0;c105963.graph_c0;c115728.graph_c0;c117552.graph_c0;c124199.graph_c0;c125404.gra	K03094+K10144+K03094+K03347+K10143+K10575+K12456

						ph_c0;c131884.graph_c0	
mRNA surveillance pathway	ko03015	7	452	408	13897	c101917.graph_c0;c102019.graph_c0;c120023.graph_c0;c122572.graph_c0;c126899.graph_c0;c134621.graph_c0;c88662.graph_c0	K11583+K12882+K13126+K11584+K13126+K12882+K03265
Circadian rhythm - plant	ko04712	6	88	408	13897	c112186.graph_c0;c122197.graph_c0;c124199.graph_c0;c126452.graph_c1;c130482.graph_c0;c93168.graph_c0	K16166+K12127+K10143+K12130+K12118+K00660
Fructose and mannose metabolism	ko00051	6	201	408	13897	c104104.graph_c0;c114913.graph_c0;c118742.graph_c0;c120249.graph_c0;c122856.graph_c0;c130978.graph_c0	K00895+K00895+K00895+K00844+K00011+K00895
Thiamine metabolism	ko00730	6	45	408	13897	c108150.graph_c0;c112666.graph_c0;c121244.graph_c0;c121595.graph_c0;c121595.graph_c1;c127214.graph_c1	K01662+K03146+K03146+K01662+K01662+K04487
Flavonoid biosynthesis	ko00941	6	64	408	13897	c111410.graph_c0;c124882.graph_c0;c126380.graph_c0;c128275.graph_c0;c129786.graph_c1;c93168.graph_c0	K13065+K05277+K13082+K05280+K00475+K00660
Purine metabolism	ko00230	6	563	408	13897	c112310.graph_c0;c119749.graph_c0;c121893.graph_c0;c123524.graph_c0;c130274.graph_c0;c64367.graph_c0	K11808+K01466+K01081+K00873+K01427+K02326
Amino sugar and nucleotide sugar metabolism	ko00520	6	335	408	13897	c113248.graph_c0;c120249.graph_c0;c123062.graph_c0;c124235.graph_c0;c126509.graph_c1;c126605.graph_c0	K00012+K00844+K01784+K01784+K00012+K00975
Fatty acid degradation	ko00071	6	197	408	13897	c108564.graph_c0;c118104.graph_c0;c118228.graph_c1;c123502.graph_c0;c133558.graph_c0;c71372.grap	K00001+K18857+K00121+K00232+K00232+K01897

						h_c0	
Glycine, serine and threonine metabolism	ko00260	5	226	408	13897	c115387.graph_c0;c116989.graph_c0;c122230.graph_c0;c131042.graph_c0;c96010.graph_c0	K15893+K00827+K01695+K00276+K02437
Pyruvate metabolism	ko00620	5	357	408	13897	c120295.graph_c0;c123524.graph_c0;c129490.graph_c0;c134605.graph_c0;c62501.graph_c0	K01895+K00873+K00026+K01610+K01895
SNARE interactions in vesicular transport	ko04130	5	99	408	13897	c109420.graph_c0;c118823.graph_c0;c122232.graph_c1;c132440.graph_c0;c86325.graph_c0	K08486+K08503+K08486+K08492+K08507
Porphyrin and chlorophyll metabolism	ko00860	5	131	408	13897	c121882.graph_c0;c123143.graph_c1;c127890.graph_c0;c127890.graph_c1;c98055.graph_c0	K04035+K03428+K03403+K03403+K03428
Alanine, aspartate and glutamate metabolism	ko00250	5	240	408	13897	c116989.graph_c0;c119465.graph_c0;c125256.graph_c1;c127467.graph_c0;c130166.graph_c0	K00827+K00261+K01955+K01580+K01953
Ascorbate and aldarate metabolism	ko00053	5	134	408	13897	c113248.graph_c0;c119013.graph_c0;c125233.graph_c1;c126509.graph_c1;c70537.graph_c0	K00012+K00469+K00225+K00012+K00423
Pentose phosphate pathway	ko00030	4	196	408	13897	c119582.graph_c1;c124834.graph_c0;c50718.graph_c0;c51688.graph_c0	K01057+K00615+K01057+K01057
Glyoxylate and dicarboxylate metabolism	ko00630	4	252	408	13897	c115387.graph_c0;c129490.graph_c0;c133476.graph_c0;c96010.graph_c0	K15893+K00026+K01681+K02437
Steroid biosynthesis	ko00100	4	81	408	13897	c101929.graph_c0;c125112.graph_c0;c130196.graph_c0;c97018.graph_c0	K01824+K14423+K14423+K01853
Protein export	ko030	4	165	408	13897	c109348.graph_c0;c129331.graph_c0;c93255.graph_c	K09490+K12948+K09648+K03109

	60					0;c93513.graph_c0	
Sulfur metabolism	ko00920	4	92	408	13897	c124334.graph_c0;c130262.graph_c0;c132024.graph_c0;c134598.graph_c0	K01738+K13034+K01738+K01738
Citrate cycle (TCA cycle)	ko00020	4	304	408	13897	c129490.graph_c0;c133476.graph_c0;c134605.graph_c0;c49644.graph_c0	K00026+K01681+K01610+K00030
Biosynthesis of unsaturated fatty acids	ko01040	4	111	408	13897	c111947.graph_c0;c115084.graph_c0;c123502.graph_c0;c133558.graph_c0	K10256+K10251+K00232+K00232
Glycerolipid metabolism	ko00561	4	191	408	13897	c122856.graph_c0;c125607.graph_c1;c130294.graph_c0;c70983.graph_c0	K00011+K03715+K13508+K13508
Carotenoid biosynthesis	ko00906	4	64	408	13897	c116858.graph_c0;c122440.graph_c0;c128720.graph_c0;c91224.graph_c0	K02291+K09840+K09841+K09843
Fatty acid elongation	ko00062	4	86	408	13897	c101374.graph_c0;c108824.graph_c0;c115084.graph_c0;c126407.graph_c0	K15397+K01074+K10251+K15397
Inositol phosphate metabolism	ko00562	4	196	408	13897	c106470.graph_c0;c119013.graph_c0;c120471.graph_c0;c122346.graph_c0	K05857+K00469+K00915+K01114
Phosphatidylinositol signaling system	ko04070	3	194	408	13897	c106470.graph_c0;c112144.graph_c0;c120471.graph_c0	K05857+K02183+K00915
Base excision repair	ko03410	3	137	408	13897	c124043.graph_c0;c133222.graph_c0;c64367.graph_c0	K10798+K10798+K02326
Ribosome biogenesis in eukaryotes	ko03008	3	379	408	13897	c113016.graph_c1;c116900.graph_c0;c130536.graph_c0	K07936+K12619+K11131

beta-Alanine metabolism	ko00410	3	176	408	13897	c118938.graph_c0;c127467.graph_c0;c131042.graph_c0	K05605+K01580+K00276
Proteasome	ko03050	3	264	408	13897	c116158.graph_c0;c118618.graph_c0;c130122.graph_c0	K02728+K02727+K03062
Propanoate metabolism	ko00640	3	140	408	13897	c118938.graph_c0;c120295.graph_c0;c62501.graph_c0	K05605+K01895+K01895
Nitrogen metabolism	ko00910	3	100	408	13897	c119465.graph_c0;c124742.graph_c0;c125396.graph_c0	K00261+K00366+K01674
Isoquinoline alkaloid biosynthesis	ko00950	3	54	408	13897	c123894.graph_c0;c130866.graph_c0;c131042.graph_c0	K01592+K01592+K00276
Fatty acid biosynthesis	ko00061	3	136	408	13897	c100226.graph_c0;c124335.graph_c0;c71372.graph_c0	K09458+K10781+K01897
Pyrimidine metabolism	ko00240	3	456	408	13897	c121893.graph_c0;c125256.graph_c1;c64367.graph_c0	K01081+K01955+K02326
Homologous recombination	ko03440	3	157	408	13897	c123433.graph_c0;c127211.graph_c0;c129332.graph_c0	K10875+K04482+K10879
Brassinosteroid biosynthesis	ko00905	2	20	408	13897	c105790.graph_c0;c131529.graph_c0	K12637+K15639
Other glycan degradation	ko00511	2	81	408	13897	c123951.graph_c0;c128732.graph_c0	K01191+K15923
Butanoate metabolism	ko00650	2	85	408	13897	c112284.graph_c0;c127467.graph_c0	K01641+K01580
Taurine and hypotaurine metabolism	ko00430	2	26	408	13897	c121597.graph_c0;c127467.graph_c0	K10712+K01580

Biotin metabolism	ko00780	2	39	408	13897	c100226.graph_c0;c124338.graph_c0	K09458+K00652
Nicotinate and nicotinamide metabolism	ko00760	2	43	408	13897	c121893.graph_c0;c122062.graph_c0	K01081+K03517
Ether lipid metabolism	ko00565	2	87	408	13897	c121339.graph_c0;c122346.graph_c0	K01115+K01114
Arginine and proline metabolism	ko00330	2	287	408	13897	c119465.graph_c0;c130274.graph_c0	K00261+K01427
DNA replication	ko03030	2	216	408	13897	c121683.graph_c0;c64367.graph_c0	K10745+K02326
Regulation of autophagy	ko04140	2	134	408	13897	c120184.graph_c0;c128260.graph_c0	K07198+K07198
Valine, leucine and isoleucine degradation	ko00280	2	223	408	13897	c112284.graph_c0;c118938.graph_c0	K01641+K05605
Basal transcription factors	ko03022	2	135	408	13897	c114404.graph_c0;c124056.graph_c0	K03139+K03120
2-Oxocarboxylic acid metabolism	ko01210	2	216	408	13897	c133476.graph_c0;c49644.graph_c0	K01681+K00030
Monoterpenoid biosynthesis	ko00902	2	6	408	13897	c102174.graph_c0;c129369.graph_c0	K15086+K15095
Sphingolipid metabolism	ko00600	2	111	408	13897	c132695.graph_c0;c133691.graph_c1	K00654+K04713

Sulfur relay system	ko04122	2	37	408	13897	c127214.graph_c1;c127644.graph_c0	K04487+K03639
Linoleic acid metabolism	ko00591	2	39	408	13897	c102687.graph_c0;c134411.graph_c0	K00454+K00454
Degradation of aromatic compounds	ko01220	2	27	408	13897	c108564.graph_c0;c118228.graph_c1	K00001+K00121
Diterpenoid biosynthesis	ko00904	1	25	408	13897	c89332.graph_c0	K17982
Pantothenate and CoA biosynthesis	ko00770	1	80	408	13897	c114123.graph_c0	K02201
Folate biosynthesis	ko00790	1	47	408	13897	c127644.graph_c0	K03639
Nucleotide excision repair	ko03420	1	225	408	13897	c64367.graph_c0	K02326
Phagosome	ko04145	1	348	408	13897	c132440.graph_c0	K08492
Flavone and flavonol biosynthesis	ko00944	1	6	408	13897	c128275.graph_c0	K05280
Selenocompound metabolism	ko00450	1	89	408	13897	c120855.graph_c0	K01761
Vitamin B6 metabolism	ko00750	1	38	408	13897	c90381.graph_c0	K13248
N-Glycan biosynthesis	ko00510	1	156	408	13897	c117730.graph_c0	K00737
Ubiquinone and	ko001	1	74	408	13897	c119082.graph_c0	K01904

other terpenoid-quinone biosynthesis	30						
Vancomycin resistance	ko01502	1	2	408	13897	c108176.graph_c0	K02563
Riboflavin metabolism	ko00740	1	26	408	13897	c112285.graph_c0	K14652
Stilbenoid, diarylheptanoid and gingerol biosynthesis	ko00945	1	34	408	13897	c111410.graph_c0	K13065
Sesquiterpenoid and triterpenoid biosynthesis	ko00909	1	22	408	13897	c124467.graph_c0	K15803
Tropane, piperidine and pyridine alkaloid biosynthesis	ko00960	1	67	408	13897	c131042.graph_c0	K00276
Arachidonic acid metabolism	ko00590	1	51	408	13897	c108187.graph_c0	K00432
Non-homologous end-joining	ko03450	1	24	408	13897	c128093.graph_c0	K10886
ABC transporters	ko02010	1	155	408	13897	c134352.graph_c0	K05658
Anthocyanin	ko009	1	4	408	13897	c117163.graph_c0	K12338

biosynthesis	42						
Synthesis and degradation of ketone bodies	ko000 72	1	27	408	13897	c112284.graph_c0	K01641
Aminoacyl-tRNA biosynthesis	ko009 70	1	289	408	13897	c78250.graph_c0	K01893

Table S8. Expression profiles of differentially expressed genes involved in petal variegation of *P.rockii.*

Genes	Unigene ID	FRKM		
		**Po**	**Pr**	**RO**
PAL	c133129.graph_c0	45	15.71	9.94
CHS	c102425.graph_c0	6680.96	2525.95	4231.85
CHI	c105653.graph_c0	5.57	71.34	9.4
DFR	c126380.graph_c0	26.18	49.13	153.27
FLS	c102035.graph_c0	3.65	7.3	3.87
F3'H	c128275.graph_c0	5.73	19.83	20.54
F3H	c129786.graph_c1	2.47	0	0.34
ANS	c124882.graph_c0	65.93	293.27	153.75
AOMT	c114770.graph_c0	6883.707434	1307.0698	99.2578947
	c92897.graph_c0	22.26	9.44	17
3GT	c132750.graph_c0	231.07	385.93	182.14
	c117163.graph_c0	27.36718756	3.8774987	3.262678295
5GT	c132577.graph_c0	17.47	11.14	11.18
	c116462.graph_c0	23.86	61.85	33.73
	c166326.graph_c0	0.22	0.27	0.12
	c122681.graph_c0	61.64	55.66	116
	c112965.graph_c0	5.96	2.35	6.24
	c105277.graph_c0	5.55	2.62	4.52
	c115951.graph_c0	78.64	600.85	275.64
	c71042.graph_c0	42.01	5.7	53.3
GST	c117370.graph_c0	1.76	0.61	0.6
	c98149.graph_c0	11.43	63.24	349.05
	c125111.graph_c0	266.23	1919.92	914.62
	c101895.graph_c0	3.64	8.03	14.51
	c113610.graph_c0	6.36	2.41	1.04
	c105192.graph_c0	0.71	12.93	6.79
	c96247.graph_c0	123.23	7.99	128.79
	c98296.graph_c0	102.28	60.62	47.71

Table S9. Differentially expressed three transcription factors families between different colored petals.

Gene name	Unigene ID	FRKM		
		PO	PR	RO
R2R3-MYB transcription factor	c158927.graph_c0	0.00	0.52	0.00
	c84113.graph_c0	0.32	0.10	0.60
	c119563.graph_c0	0.93	0.25	0.16
	c127075.graph_c0	2.22	0.50	1.12
	c131300.graph_c0	0.00	0.28	0.71
	c119993.graph_c0	2.76	1.56	0.52
	c115179.graph_c0	36.78	11.72	20.96
	c131300.graph_c1	0.00	0.28	0.71
	c124756.graph_c0	5.13	5.95	1.38
	c112186.graph_c0	11.97	0.10	25.30
	c128847.graph_c0	21.56	11.81	32.35
	c133735.graph_c0	0.34	2.74	1.37
	c117848.graph_c0	0.02	5.48	0.33
	c115438.graph_c0	0.24	0.92	0.29
bHLH transcription factor	c114227.graph_c0	10.53	4.43	6.97
	c102655.graph_c0	27.40	38.01	5.79
	c124854.graph_c0	12.09	23.44	22.01
	c130824.graph_c0	1.32	0.37	0.68

Table S10. Bioanalyzer results of all RNA samples.

Samples	No.of biological replicates	RIN value	rRNA ratio (28S/18S)	Base line	OD 260/280 value	OD 260/230 value	RNA concentration (ng/μl)	Volume (μl)
PO	1	10	2	Normal	2.22	2.26	820.2	25
	2	10	1.9	Normal	2.21	2.47	1056.9	25
	3	9.9	2	Normal	2.37	2.37	1067.8	25
PR	1	9.9	2.1	Normal	2.21	2.2	1386.6	25
	2	10	2	Normal	2.19	2.27	1571.7	25
	3	10	1.8	Normal	2.2	2.21	912.3	25
RO	1	8.9	2.3	Normal	2.2	2.27	436	25
	2	9.8	1.9	Normal	2.23	2.2	674.1	25
	3	9.9	1.8	Normal	2.19	1.97	455.2	25

Table S11. The gene names, sequences and all primers used for qRT-PCR analysis.

Gene	Unigene ID	Forward primer(5'-3')	Reverse primer (5'-3')
PAL-1	c133129.graph_c0	AAGTGATCCGTTCGTCAACC	TTGGGGTACCTTGGAAGTTG
CHS-1	c102425.graph_c0	TATTGTCGGTGCTGATCCTG	TCACGCAAGTGTCCATCAAT
CHI-1	c105653.graph_c0	ATGGTGACGGGAAAGTGAAG	CTGCTAAGCTCTGCCTTGCT
F3H-1	c129786.graph_c1	AGGCTTTATGGTGCCCTGTA	GCGAGGGAGATATTGGGATT
FLS -1	c102035.graph_c0	CCTATAATCGGCCATCTCCA	ACAGCACAAGGGATTGAACC
DFR-1	c126380.graph_c0	TTCCAAGGACCCTGAGAATG	GTTCCTGCAGATGACGTGAA
5GT-1	c132577.graph_c0	TGCCAAGCTGATTGAAGATG	CTCTCCACCTCCCATAACCA
ANS-1	c124882.graph_c0	AGAAATCGATTCGGAGGACA	CCCTTTCCAAAAGCTCATCA
AOMT-1	c92897.graph_c0	AAGTGAAGCCCGTTTGTTCA	TCATGCTCCCTTGGGTAGAC
GST-1	c105192.graph_c0	TCAGAGGGAATGGATGAAGC	CCATTACGCAACTGACGATG
MYB-1	c119993.graph_c0	CGGAGAAATCGAAGAACTCG	TCCATCCCCAAGAAGAAGTG
bHLH-1	c114227.graph_c0	GAGCTCACCAACCTCGAAAC	TCGGTCTTCGCAACATAGTG
TUB	c106431.graph_c0	AGGTAAGATGAGCACCAAAG	GGAGGAATGTCACAAACACT
